# Access to Unsupported Iminobismuthane and Tetraazabismole Species Enabled by a Rigid and Bulky Organobismuthinidene

**DOI:** 10.1002/anie.2998568

**Published:** 2026-09-18

**Authors:** Marcel Potocnik, Kristian L. Mears, Davide Spinnato, Gabriele Deplano, Nils Nöthling, Markus Leutzsch, Maurice van Gastel, Khai‐Nghi Truong, Serena DeBeer, Josep Cornella

**Affiliations:** ^1^ Max‐Planck‐Institut für Kohlenforschung Mülheim an der Ruhr Germany; ^2^ Max‐Planck‐Institute For Chemical Energy Conversion Mülheim an der Ruhr Germany; ^3^ Rigaku Europe SE Neu‐Isenburg Germany

**Keywords:** azides, bismuth, catalysis, heterocycles, iminobismuthanes

## Abstract

Herein, we report the reactivity of a monocoordinate bismuthinidene (**1**) with various organic azides. The reaction of **1** with *p*‐toluenesulfonyl azide resulted in the formation of an unprecedented, unsupported iminobismuthane (**2**), which features the shortest reported Bi–N distance of 2.041(3) Å in an iminobismuthane. Conversely, the reaction of **1** with 4‐azidobiphenyl resulted in the first example of a species containing a tetraazabismole (BiN_4_) heterocycle (**3**). We also highlight that **1** demonstrates catalytic activity, enabling the reduction of a selection of organic azides.

## Graphical abstract

By employing a sterically bulky monocoordinate bismuthinidene, the synthesis and characterization of the first examples of an unsupported iminobismuthane and tetraazabismole was achieved via reactions with organic azides. The corresponding amines could be accessed by reduction, leading to the regeneration of the Bi(I) compound. Hence, we also highlight the first example of a catalytic Bi(I)/Bi(III) redox cycle of a monocoordinated bismuthinidene.

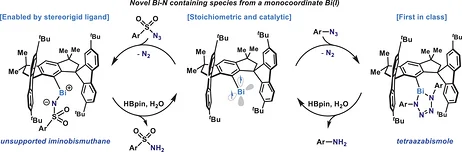

## 1 Introduction

The reactivity of transition metals (TM) with organic azides has cultivated a rich area of research [1, 2, 3, 4], both in terms of accessing new coordination complexes, as well as developing N‐atom transfer pathways for catalysis. The former has afforded access to TM azido‐ [5, 6, 7, 8, 9, 10, 11], imido‐ [11, 12, 13, 14], and tetraazido‐species [13, 15, 16, 17], while the latter has enabled the construction of N‐containing organic molecules [18, 19]. In more recent decades, these coordination and reactivity studies have crossed over to the main group, with metals from groups 13–15 also unlocking imido‐based reactivity that both mimics and diverges from that of previous TM reports [20, 21, 22, 23, 24, 25, 26, 27, 28, 29, 30, 31, 32, 33, 34]. Despite these precedents in the main group, examples of iminobismuthanes still remain scarce. Since the initial report of the isolation of an iminobismuthane from Wittig and Hellwinkel in 1964 [35], studies on iminobismuthanes have been mostly limited to high‐valent Bi(V) iminobismuthane compounds (of the general formula Ar_3_Bi═NR) [36, 37, 38]. In 1998, the first structural characterization of such a compound was reported by Suzuki and Ikegami (Figure 1a) [38], with single‐crystal X‐ray diffraction (SC‐XRD) analysis suggesting that this compound's imide bond is best described as a polarized Bi^+^─N^−^ single bond, with a bond length of 2.13(1) Å. Further reports followed [36], which varied aryl ligands and the nature of the imide ‐N*R* group, but all were still limited to Bi(V).

**FIGURE 1:**
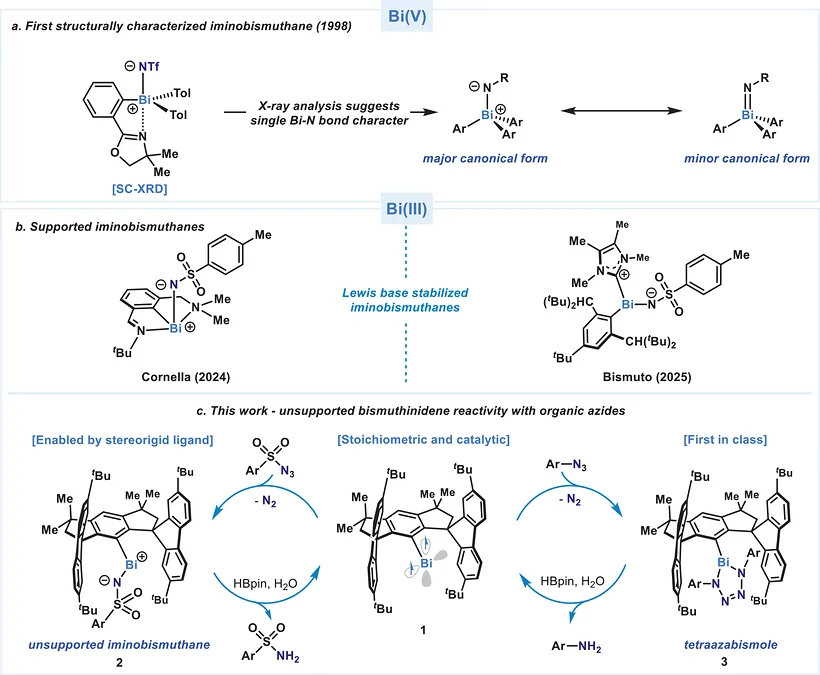
Illustrations of (a) ligand design strategy employed to isolate the first crystalline example of an iminobismuthane, and the canonical forms that explain the observed Bi─N bond lengths; (b) Previous examples of bismuthinidene reactivity with organic azides using an *N,C,N*‐pincer ligated Bi complex; (c) This work: access to unsupported iminobismuthane and tetraazabismole species.

Our group has been investigating bismuth's versatility in redox transformations, including polar [Bi(III)/Bi(V), Bi(I)/Bi(III)] and radical [Bi(I)/Bi(II)/Bi(III)] catalytic cycles [39, 40, 41, 42, 43, 44, 45, 46, 47, 48, 49], as well as new fundamental Bi species that exhibit unique coordination chemistry and electronic properties [50, 51]. In this vein, we have demonstrated that *N,C,N*‐pincer bismuthinidenes react with organic azides to afford monomeric iminobismuthanes (Figure 1b) [52], where the first example of a Bi(III)‐imido bond could be successfully characterized by SC‐XRD. All these derivatives are highly reactive and challenging to handle. Yet, they all feature a supporting *N,C,N*‐pincer ligand, in which the lone pairs of the N atoms donate into the empty 6p orbitals of the Bi(III), thus providing additional stabilization to the Bi atom. Likewise, Bismuto and coworkers have recently reported another iminobismuthane example, where the Bi center is stabilized via coordination of an NHC ligand (Figure 1b) [53]. These stabilized iminobismuthane precedents certify that the synthesis and isolation of an unsupported analogue, deprived of additional coordinating features, would be significantly more challenging to access. However, we have recently reported that a *^t^* BuM^s^Fluind ligand scaffold can be used to generate a monocoordinate bismuthinidene (**1**, Figure 1c) [49]. We speculated that the rigidity of this particular ligand platform would be ideal to synthesize and isolate a monocoordinate, unsupported iminobismuthane. Here, we report that **1** rapidly reacts with tosylazide, leading to an unsupported (donor‐free) iminobismuthane featuring a short, formal Bi═N double bond. Additionally, we show that the rigid cavity around the Bi center also permits the kinetic stabilization required for the synthesis, isolation and characterization of an elusive tetraazabismole (*i.e*., BiN_4_) heterocycle (Figure 1c), a feature beyond the possibilities of other low‐valent Bi(I) compounds.

## 2 Results and Discussion

Previously, we showed that pincer‐supported bismuthinidines rapidly react with organic azides to form monomeric iminobismuthanes, with the Bi center coordinated to Lewis‐basic nitrogen atoms from the supporting imine/amine arms [52]. Similarly, **1** also reacts smoothly with *p*‐toluenesulfonyl azide (Figure 2, top), yielding a dark red solution and visible N_2_(g) formation. Storing the solution at 25°C afforded product **2** as a crystalline solid that could be analyzed using SC‐XRD (Figure 2, bottom). Structural analysis revealed that **2** is an unsupported iminobismuthane, featuring an extremely short Bi─N bond (2.041(3) Å). This bond length suggests partial double‐bond character between Bi and N, as the non‐stabilized and electron‐deficient Bi(III) center likely enhances orbital overlap, contracting the Bi─N bond (*vide infra*). The C─Bi bond in **2** is slightly out of the plane of the phenyl ring to accommodate the imido group. **2** was fully characterized by NMR spectroscopy, HRMS, UV‐Vis and FTIR spectroscopy, thus providing additional characterization insights into its structure (Figures S1–S10).

**FIGURE 2:**
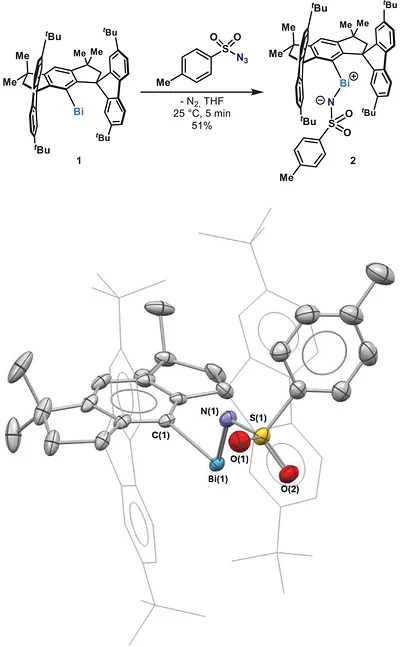
Top: synthetic route to compound **2**. Bottom: molecular structure of **2**. Thermal ellipsoids are shown at 50% probability. Side‐arms of the *^t^* BuM^s^Fluind ligand are shown in wireframe format, hydrogen atoms, a co‐crystallized THF molecule and atom sites with disordered parts are not shown for clarity.

With the validation of **1** as an active reductant for organic azides, we explored the breadth of this reactivity with other synthetically interesting aromatic azides (Ar–N_3_), such as 1‐azido‐4‐(trifluoromethyl)benzene and 4‐azidobiphenyl. The absence of a sulfonyl group on the azide introduces two potential challenges: (i) these azides are less electrophilic, thus slowing down their activation by the Bi(I), and (ii) the potential Bi═N product cannot delocalize the negative charge onto the sulfonyl group, reducing thermodynamic stability. The reaction of **1** with 4‐azidobiphenyl (Figure 3, top) produced a light purple solution, contrasting with the dark red solution observed for **2**. Precipitation with pentane yielded a microcrystalline powder, stable in air as a solid but decomposing rapidly in solution at room temperature.

**FIGURE 3:**
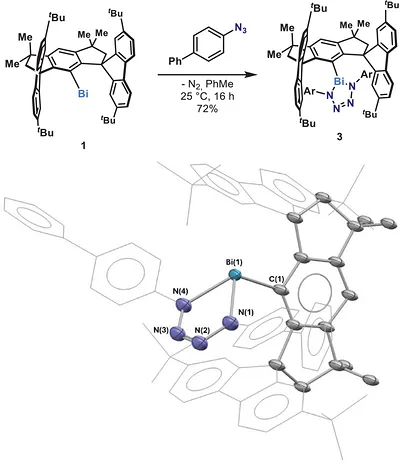
Top: synthetic route to compound **3**. Bottom: molecular structure of **3**, [Bi(N_4_{1,1’‐Ph_2_}_2_)(*^t^* BuM^s^Fluind)]. Thermal ellipsoids are shown at 50% probability. The ligand is shown in wireframe format and hydrogen atoms are not shown for clarity.

^1^H NMR spectroscopic analysis under inert conditions revealed a 1:2 ratio between the protons of the *^t^* BuM^s^Fluind ligand and the aromatic protons originating from the organic azide. To aid in product characterization, diffraction techniques including SC‐XRD were attempted, but efforts to obtain suitable quality crystals were unsuccessful, though microcrystalline powders were reproducibly obtained. In order to gain further insight into the connectivity of this elusive molecule, the microcrystalline solid was analyzed by continuous rotation 3D electron diffraction (3DED) [54, 55]. Similar techniques based on electron‐diffraction (MicroED) have previously been successfully employed for analyzing inorganic compounds [56, 57], as exemplified by Stoltz, Rodriguez and Nelson, who were able to structurally characterize Schwartz's reagent or analogous palladium compounds. We were able to obtain diffraction data using this technique that allows us to solve and refine the structure of **3**, which corresponded to the only example of a tetraazabismole, featuring a 5‐membered ring BiN_4_ heterocycle, flanked by the *^t^* BuM^s^Fluind ligand (Figure 3, bottom).

The reaction between **1** and 1‐azido‐4‐(trifluoromethyl)benzene yielded a species that we tentatively assigned as the corresponding tetraazabismole (compound **4**, see Suppporting Information) though, this could only be identified and characterized by NMR analysis, again due to the difficulty in obtaining pure, solid material after workup. NMR spectroscopic evidence for the formation of compound **4** is provided in (Figures S25–S33).

In addition, the reactivity of **1** with *ortho*‐substituted aryl azides was also explored. However, for these organic azides, formation of iminobismuthane or tetraazabismole‐like species was not observed, likely due to the incompatibility between the bulky aryl azides and the *^t^* BuM^s^Fluind ligand. Similarly, no reactivity was observed between **1** and primary or secondary alkyl azides, on account of their more electron‐rich nature.

We investigated the reactivity of compound **2**, specifically in comparison to our previously reported work on the reduction of organic azides via bismuth redox catalysis [52]. For a direct comparison, we attempted the reduction of iminobismuthane **2** to the bismuthinidene **1** using pinacolborane (HBpin) as a reductant. Treatment of **2** with 1.0 equiv. of HBpin and H_2_O resulted in rapid formation of **1** and corresponding sulfonamide in quantitative yields at 25°C (Scheme 1a). Likewise, the successful reduction of **3** to 4‐aminobiphenyl in quantitative yields was also achieved, although an excess of reductant was necessary (4 equiv. of HBpin and H_2_O) with only partial recovery of **1** (Scheme 1b). Based on this stoichiometric reactivity, catalytic conditions were examined. Using 5 mol% of **1** with HBpin, H_2_O, and *p*‐toluenesulfonyl azide (1 equiv.) in THF‐*d_8_* resulted in quantitative (>99%) formation of the sulfonamide in 5 min at 25°C (Scheme 1c). Repetition of the reaction without water led to the quantitative formation of the *N*‐borylated sulfonamide (Figure S45), underlining the role of HBpin as reducant, with H_2_O serving to cleave the resulting B─N bond to afford the NH_2_ functionality, in line with similar reports [58]. Impressively, when 4‐acetamidobenzenesulfonyl azide was used as substrate, not only was complete reduction of the azide observed, but also total regeneration of catalyst **1**. We then assessed different aryl azides at 25 °C over 16 h, including 4‐azidobiphenyl, 1‐azido‐4‐(trifluoromethyl)benzene and 1‐azido‐4‐methoxybenzene, which resulted in the formation of the corresponding aryl amines in 15%, 75% and 47% yield, respectively (Scheme 1d). Finally, for substrates that demonstrated no stoichiometric reactivity with **1**, all attempts at catalysis proved unsuccessful, highlighting the necessity of a Bi─N containing intermediate for this transformation. We emphasize here that this work represents the only reported example of a monocoordinated bismuthinidene displaying catalytic reactivity in a redox process. The redox catalysis of **1** is currently being explored further in our group.

**SCHEME 1:**
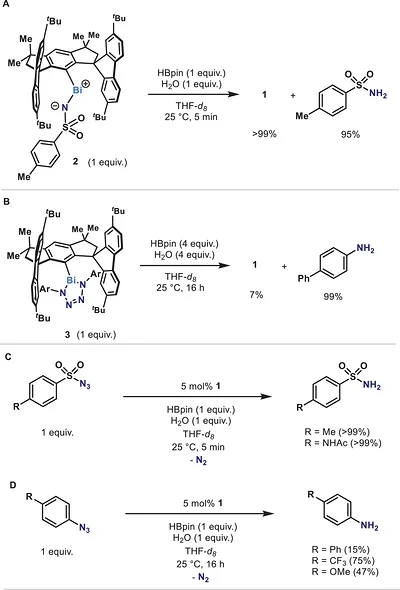
1A: Reduction of iminobismuthane **2** with pinacolborane (HBpin). 1B: Reduction of tetraazabismole **3** with pinacolborane. 1C: Catalytic reduction of sulfonyl azides to the corresponding sulfonamides. 1D: Catalytic reduction of aryl azides to the corresponding aryl amines. Yields were determined by ^1^H NMR spectroscopy using 1,3,5‐trimethoxybenzene as an internal standard.

Compound **3** crystallizes in the space group *P*2_1_/*n* with eight molecules per unit cell and two independent molecules in the asymmetric unit. The observed average Bi─C distance is 2.320(20) Å, which is slightly longer than the distance for the uncoordinated **1** (2.2783(10) Å) [51]. The average N─N distances (N6─N7 and N2─N3) of 1.29(4) Å could not be determined with high accuracy from the 3DED data, but are consistent with the bond lengths observed for other main group tetraazene and iminobismuthane systems, indicating double‐bond character [21, 22, 28, 34, 36, 37, 52, 53]. The central Bi atom also deviates slightly from the ring plane of the ligand backbone aryl ring, and exhibits a slight distortion out of the Bi─C_ipso_ axis. In order to accommodate the N_4_ group inside the pocket, the *^t^* BuM^s^Fluind bends, resulting in a highly distorted backbone. We further analyzed **3** using spectroscopic and electrochemical techniques. UV‐Vis spectroscopy was employed to gain deeper insight into the electronic structure of these compounds. Experimental and simulated UV‐Vis spectra of **2** and **3** in solution are reported in Figure 4a,b, respectively.

**FIGURE 4:**
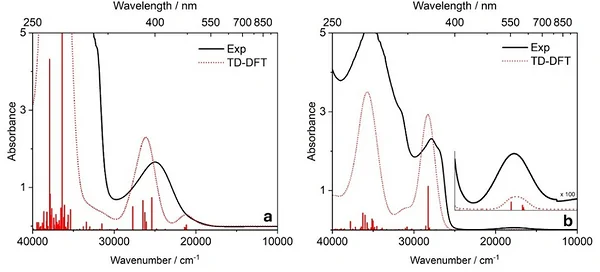
UV‐Vis spectra of **2** (panel a) and **3** (panel b) in DCM solution. Full and dotted lines represent experimental and calculated (TD‐DFT) spectra, respectively. The sticks under the dotted curves indicate the calculated transitions before broadening (2000 cm^−1^ Gaussian bandwidth). Calculated spectra have been shifted by ‐3000 cm^−1^ to match experimental features.

The calculated spectra reproduce well the experimental features in both position and relative intensity. Together with a low average root mean square deviation between the geometry obtained from diffraction experiments and DFT optimization (<0.4 Å in both cases; 0.231 and 0.302 Å for **2** and **3**, respectively), it suggests that the TD‐DFT simulated spectra approximate the electronic structure of both molecules. **2** is characterized by a broad asymmetrical band at 25 000 cm^−1^ (ε = 831 M^−1^ cm^−1^), which is assigned to a ligand to metal charge transfer (LMCT) based on orbital analysis; the low‐energy asymmetry of the band is caused by the strong Bi spin‐orbit coupling (SOC), which splits the absorption band into low energy singlet‐triplet and high energy singlet‐singlet transitions for the same orbitals (21 000 and 26 000 cm^−1^ in the calculated spectra, respectively).

The intense band above 30 000 cm^−1^ is caused by a more diffuse set of aromatic π to LUMO transitions. In contrast, **3** features a low‐energy (ε = 237 M^−1^ cm^−1^) LMCT band at 17 000 cm^−1^ that corresponds to the HOMO‐LUMO transition, which is also significantly broadened by SOC. The bands at higher energies mainly emerge from the ligand system, with π–π* transitions that involve either the tetraazido (27 900 cm^−1^) or the *^t^* BuM^s^Fluind (35 300 cm^−1^) moieties; a more detailed analysis of the UV‐Vis transitions can be found in the SI (Figures S50 and S51). In order to probe the Bi‐ligand bonding of the two compounds, we analyzed the calculated wavefunction using the Pipek‐Mezey formalism [59], which describes the occupied valence space in terms of recognizable σ and π orbitals; the results are reported in Figure 5.

**FIGURE 5:**
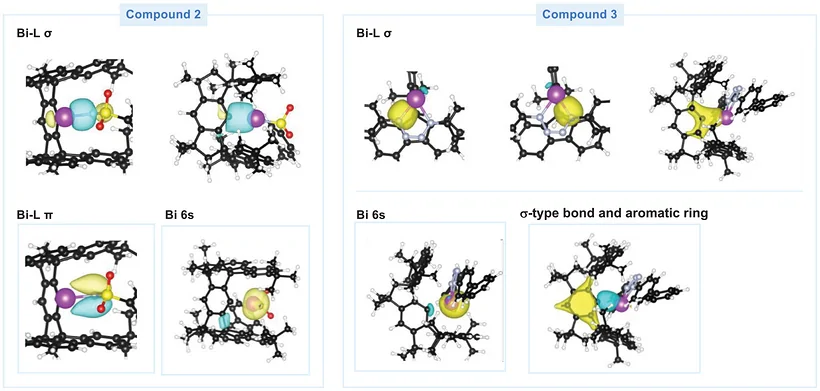
Pipek‐Mezey orbitals for **2** (left) and **3** (right). The orbitals labelled as “6s” are localized almost exclusively on Bi, while all other orbitals present a significant contribution from other atoms in the molecule. Color code: pink, Bi; yellow, S; red, O; grey, N; black, C; white, H. Iso‐surface value: 0.05 a.u.

Both compounds preserve an isolated 6s valence shell, which is mostly of atomic character and is not significantly involved in bonding; meanwhile, all other valence orbitals reported in Figure 5 and discussed hereafter are of molecular (i.e., not strictly atomically localized) nature, with significant electron delocalization on the ligands. **2** exhibits one σ bond with each ligand (through the C and N atoms), plus one additional π bond with the imido group, supporting the hypothesis of Bi═N double bond character. Natural Orbitals for Chemical Valence (NOCV) analysis further confirmed the assignment of the valence and bond orders for Bi as reported in the SI (Figure S52).

Compound **3** bonds to its ligands through three σ interactions, but it presents an additional occupied orbital in its valence space. This additional orbital also features a σ‐like shape, but with electron density delocalized over the 6‐membered aryl backbone of the *^t^* BuM^s^Fluind ligand. This would suggest a more electron‐rich Bi center, consistent with the relatively high‐energy edge position in the XAS spectrum (*vide infra*). A possible reason for this is the presence of aromatic character in the 5‐membered ring formed by BiN_4_ unit. Nucleus‐independent chemical shielding (NICS) analysis [60, 61] on the calculated structure for **3** (specifically on the geometrical center of the BiN_4_ ring structure) finds a value of 9.8, which is intermediate between that calculated for benzene (7.8) and phenylpentazole (13.0), suggesting a relatively strong ring current at the center of the BiN_4_ ring. Cyclic voltammetry (CV) of **3** displays two irreversible reduction events (Figure S22) and one oxidation event (Figure S23), consistent with an assignment of a Bi(III) species (see Supporting Information). X‐ray absorption spectroscopy (XAS) was also employed to more accurately probe the valence state of **3** (Figure 6).

**FIGURE 6:**
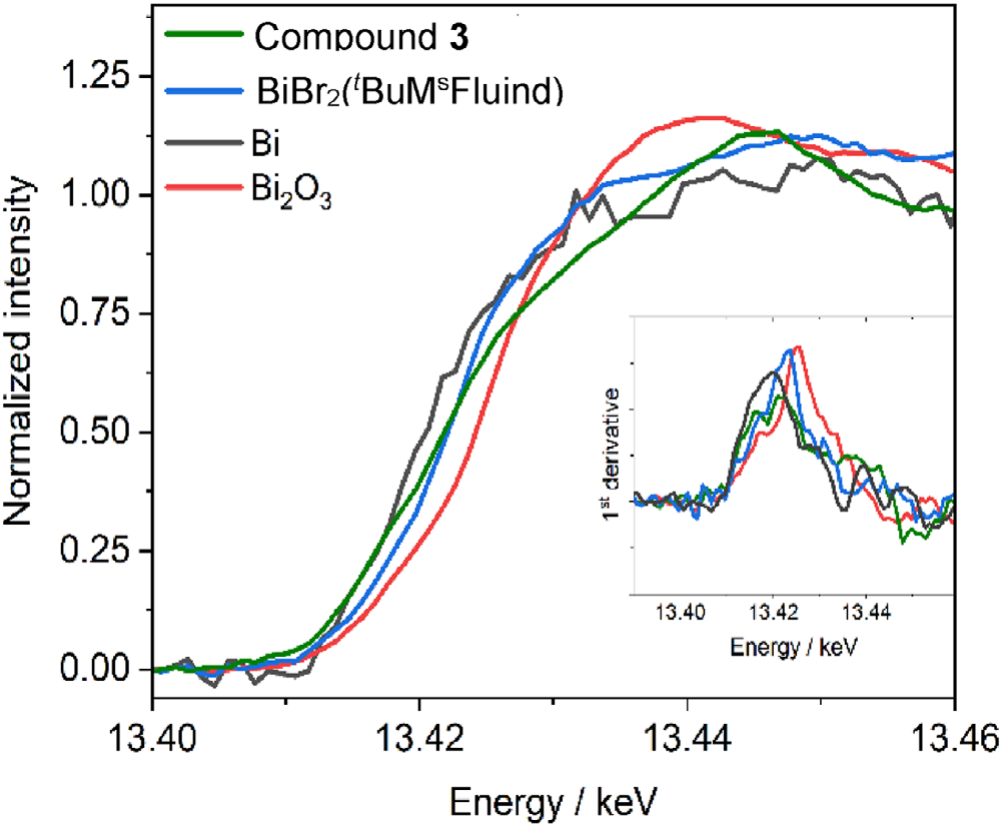
X‐ray absorption spectrum of compound **3** and reference compounds with inset of first derivative.

Although a decisive assignment is challenging from the XAS data, it is possible to conclude that the edge position shifts monotonically in the series that comprises of metallic Bi, tetraazabismole (**3**), [BiBr_2_(*^t^* BuM^s^Fluind)] and Bi_2_O_3_ (13419.0 eV, 13421.4 eV, 13423.0 eV and 13425.4 eV, respectively). The edge energy in the XAS spectra correlates with oxidation states in a series of similar compounds for a given element. Compound **3** lies between metallic Bi and Bi_2_O_3_, and above [BiBr_2_(*^t^* BuM^s^Fluind)]. Since NMR spectroscopy discarded the presence of radicals, the XAS data in combination with the CV data point toward the assignment of an electron‐rich Bi(III) metal center.

## 3 Conclusion

In conclusion, we have synthesized an unsupported iminobismuthane (**2**) and the only example of a tetraazabismole compound (**3**) from a monocoordinate bismuthinidene (**1**). Compound **2** was fully characterized [55], including structurally by SC‐XRD [60], which revealed the shortest Bi─N bond of an iminobismuthane, suggesting at least partial double‐bond character. Compound **3** was accessed by modifying the electronic properties of the azide partner, highlighting bismuth's ability to form structures analogous to those of early transition metals and its lighter pnictogen congeners. By isolating and characterizing tetraazabismole **3**, we have completed the set of tetraaza‐pnictogen compounds. We reason that this species may *only* be accessible due to the unique electronic and steric properties of our monocoordinate bismuthindene (**1**), given that multiple reported attempts at accessing tetraazabismole species have all proven unsuccessful, despite being effective for the other group 15 members [62]. This once again reinforces the unique position that bismuth occupies among the main group, offering distinct reactivity compared to its neighbors, and stressing the often overlooked role of bismuth within the chemist's toolkit. The connectivity of **3** was supported by NMR and 3DED studies, the latter of which has been instrumental in validating the hypothesis for having 4 contiguous N atoms in its structure. Compound **3** is another example of an organometallic compound structurally characterized by 3DED. This complementary technique opens up new possibilities for structural elucidation of reactive microcrystalline compounds, thanks to recent developments in instrumentation. We believe the power of 3DED is yet to be fully unlocked for organic and organometallic structure characterization and will certainly accelerate the discovery of more unusual molecular structures in the future. Electrochemical, spectroscopic and computational analysis of compound **3** suggests that **3** contains a Bi(III) metal atom, and that there is significant ring current within the BiN_4_ moiety. Finally, this report further develops the unique reactivity of the monocoordinate bismuthinidine (**1**) arising from its low‐valency. We highlight the redox‐active catalysis of **1**, in the context of reduction of either the formed iminobismuthane or tetraazabismole to afford the corresponding sulfonamide or aryl amine, as an initial example of catalytic activity of a monocoordinate Bi(I) species. Further exploitation of this platform for accessing new chemical transformations and species is ongoing.

## Author Contributions

**Marcel Potocnik**: data curation, formal analysis, investigation, methodology, validation, writing – review and editing. **Kristian L. Mears**: data curation, formal analysis, writing – original draft, writing – review and editing, supervision, investigation, validation. **Davide Spinnato**: conceptualization, data curation, formal analysis, methodology, investigation, writing – review and editing. **Gabriele Deplano**: data curation, formal analysis, writing – review and editing, methodology, validation, visualization. **Nils Nöthling**: data curation, formal analysis, writing – review and editing, investigation, methodology, validation, visualization. **Markus Leutzsch**: data curation, formal analysis, writing – review and editing. **Maurice van Gastel**: writing – review and editing, data curation, formal analysis. **Khai‐Nghi Truong**: software, validation, formal analysis, data curation, visualization. **Serena Debeer**: data curation, formal analysis, resources, funding acquisition, supervision, writing – review and editing. **Josep Cornella**: writing – original draft, writing – review and editing, conceptualization, funding acquisition, resources, supervision, project administration.

## Conflicts of Interest

The authors declare no conflicts of interest.

## Supporting information



The data that supports the findings of this study are available in the supplementary material of this article. **Supporting File**: anie74710‐sup‐0001‐SuppMat.pdf.

## Data Availability

The data that supports the findings of this study are available in the supplementary material of this article.
