# Supplementary material for: Access to Unsupported Iminobismuthane and Tetraazabismole Species Enabled by a Rigid and Bulky Organobismuthinidene
Source: Angew Chem Int Ed Engl. 2026 Sep 18;65(45):e2998568. doi: 10.1002/anie.2998568 (PMC13725612; doi:10.1002/anie.2998568)
Supplement: Supplementary file 1 [file ANIE-65-e2998568-s001.pdf]

Access to Unsupported Iminobismuthane and Tetraazabismole Species enabled by a Rigid and Bulky Organobismuthinidene

Supporting information

Marcel Potocnik,†[a] Kristian L. Mears,†[a] Davide Spinnato,[a] Gabriele Deplano,[b] Nils Nöthling,[a] Markus Leutzsch,[a] Maurice van Gastel,[a] Khai-Nghi Truong,[c] Serena DeBeer[b] and Josep Cornella*[a]

[a]Max-Planck-Institut für Kohlenforschung, Kaiser-Wilhelm-Platz 1, 45470, Mülheim an der Ruhr, Germany. [b]Max-Planck-Institute for Chemical Energy Conversion, Stiftstraße 34, 45470, Mülheim an der Ruhr, Germany. [c]Rigaku Europe SE, Hugenottenallee 167, 63263 Neu-Isenburg, Germany

cornella@kofo.mpg.de

serena.debeer@cec.mpg.de

1. General considerations **3**

2. Synthesis and Characterization of 2 - 4 .... **4**

2.1. Synthesis and characterization of 2 4
2.2. Synthesis and characterization of 3 11
2.3. Synthesis and characterization of 4 20

3. X-ray and Electron Diffraction Crystal Structure Analyses **26**

3.1. Single crystal structure analysis of 2 · THF solvate 26
3.2. Crystal structure analysis of 3 39

4. Reactivity and Catalysis **59**

4.1. Reduction of 2 with pinacolborane 59
4.2. Reduction of 3 with pinacolborane 60
4.3. Catalytic reduction of *p*-toluenesulfonyl azide 61
4.4. Catalytic reduction of 4-acetamidobenzenesulfonyl azide 62
4.5. Catalytic reduction of 4-azidobiphenyl 63
4.6. Catalytic reduction of 1-azido-4-(trifluoromethyl)benzene 64
4.7. Catalytic reduction of 1-azido-4-methoxybenzene 65
4.8. Catalytic reductive borylation of *p*-toluenesulfonyl azide 66
4.9. Unsuccessful substrates 67

5. X-ray absorption spectroscopy (XAS) **68**

6. Molecular modeling **69**

6.1 Computational details 69
6.2 IR spectroscopy 70
6.3 UV-Vis spectroscopy and natural transition orbitals (NTOs) 73
6.4 ETS-NOCV Analysis 75
6.5 Cartesian coordinates for optimized structures 77

7. References **84**

1. General considerations

All experiments were conducted in flame-dried glassware under argon atmosphere by using standard Schlenk techniques or a MBraun glovebox and freshly dried and degassed solvents. (THF, THF-d_8, toluene and *n*-pentane were distilled from potassium and stored over 4 Å molecular sieves (activated at 200 °C under a high vacuum for three days) under argon prior to use, whereas CH_2Cl_2 and CD_2Cl_2 were dried over CaH_2 and stored over 4 Å molecular sieves. Anhydrous $BiBr_3$ (97%) was purchased from Strem Chemicals, 4-acetamidobenzenesulfonyl azide and 4,4,5,5-tetramethyl-1,3,2-dioxaborolane were purchased from TCI, 1,3,5-trimethoxybenzene was purchased from BLD Pharmatech, *p*-toluenesulfonyl azide and cobaltocene (min. 98%) were purchased from Sigma Aldrich and were stored in the freezer of the glove box prior to use. ^{t}BuM^{s}Fluind-Bi(I) (**1**) and aryl azide substrates (4-azidobiphenyl, 1-azido-4-(trifluoromethyl)benzene, 1-azido-4-methoxybenzene, 2-azido-1,3,5-trimethylbenzene, 2-azido-1,3-diisopropylbenzene) were synthesized according to previously reported procedure.[1-4] All other reagents were obtained from commercial suppliers and used without further purification.

NMR data were recorded using a Bruker AVIII HD 300 MHz, a Bruker AVIII 500 MHz or a Bruker AVNeo 600 MHz NMR spectrometer. ^{1}H and ^{13}C chemical shifts are reported relative to the solvent residual peaks as an internal reference, and ^{15}N chemical shifts are reported relative to $MeNO_2$. For ^{1}H NMR the following residual proton peaks of the deuterated solvents were used: CD_2Cl_2, δ_H($CHDCl_2$) 5.32 ppm; THF-d_8, δ_H[$(CD_2)_3CHDO$] 3.580 ppm. For ^{13}C NMR: CD_2Cl_2, δ = 53.84 ppm. Unless mentioned otherwise ^{13}C spectra were generally acquired with broadband proton decoupling. ESI-MS: ESQ 3000 (Bruker). Melting points were measured with a Buchi Melting Point M-560 using flame-sealed argon-filled capillaries. UV-Vis-NIR measurements were performed at room temperature under argon using a Cary6000i spectrophotometer equipped with an InGaAs detector. IR spectra were measured using a Bruker ALPHA II compact FT-IR spectrometer in a glovebox. Electrochemical experiments were conducted using Gamry Interface 1010E potentiostat. DSC analysis was performed in a 10 μL aluminum crucible using 5 mg of sample. The details of single crystal X-ray diffraction analysis, and computational studies are described in the following sections.

For stoichiometric and catalytic experiments, yields were determined by relative integration between 1,3,5-trimethoxybenzene (6.06 ppm, 3H) and the product.

2. Synthesis and Characterization of 2 - 4

2.1. Synthesis and characterization of 2

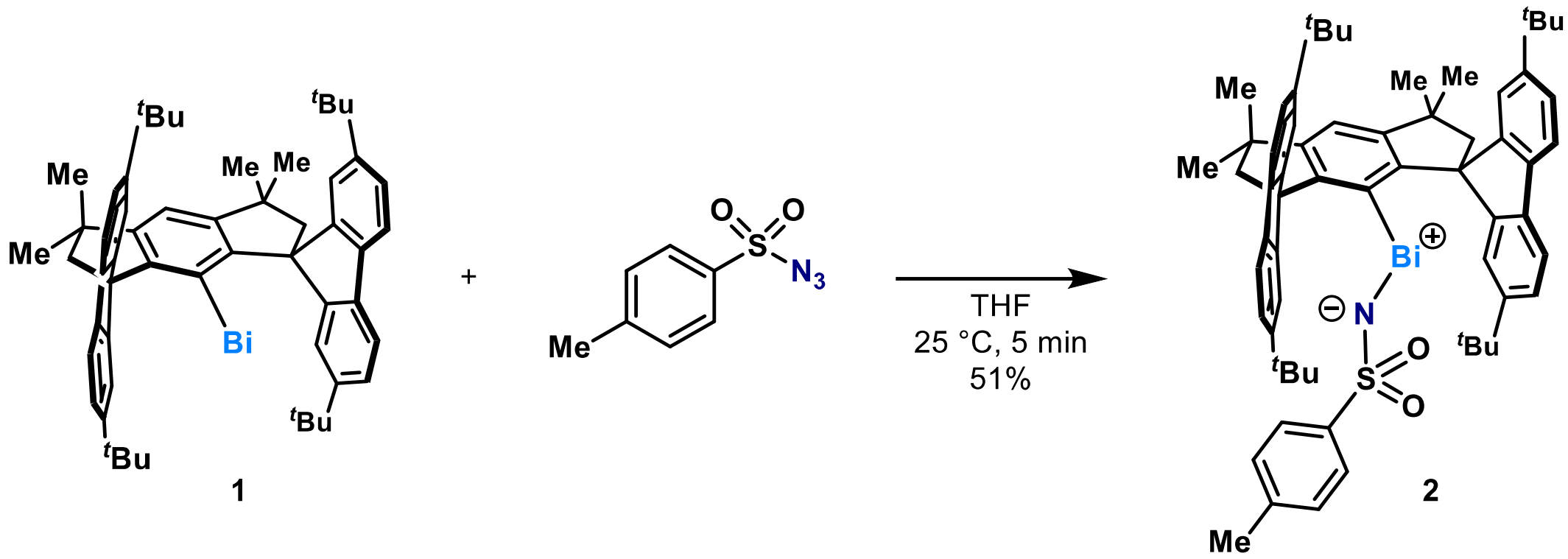

Procedure: In an argon-filled glove box, **1** (10 mg, 0.011 mmol, 1.0 equiv.) and *p*-toluenesulfonyl azide (2.1 mg, 0.011 mmol, 1.0 equiv.) were weighed in an oven-dried 12 mL vial, followed by the addition of 0.15 mL of tetrahydrofuran. Upon stirring at 25 °C, a dark red solution is formed. After 5 min, the solution is filtered and the filtrate is layered with pentane and left standing inside of the glove box. After 18 h, red crystals formed, which were washed with toluene (2 mL) and pentane (4 mL) to obtain the desired product in 51% yield (6.0 mg, 0.0054 mmol).

Characterization data of **2**:

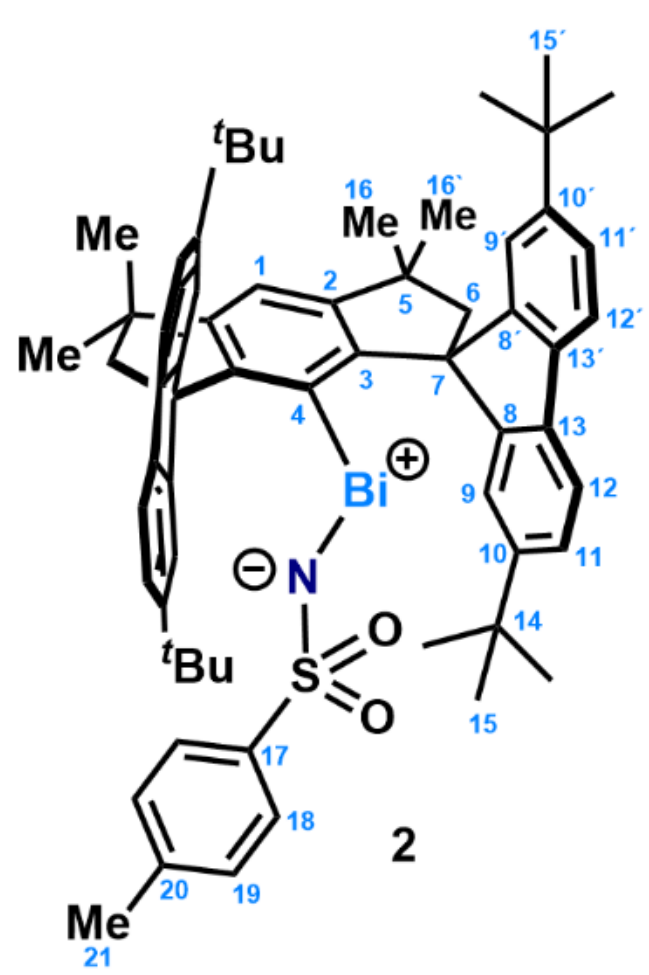

^{1}H NMR (600 MHz, CD_2Cl_2, 243 K)*: δ 7.62 (d, *J* = 8.1 Hz, 2H, H-12'), 7.59 (s, 1H, H-1), 7.38 (dd, *J* = 8.1, 1.8 Hz, 2H, H-11'), 7.27 – 7.22 (m, 2H , H-18), 7.23 (d, *J* = 1.7 Hz, 2H, H-9), 7.18 (d, *J* = 1.8 Hz, 2H, H-9'), 7.17 – 7.12 (m, 2H, H-19), 6.92 (d, *J* = 8.0 Hz, 2H, H-12), 6.86 (dd, *J* = 8.0, 1.7 Hz, 2H, H-11), 2.55 (d, *J* = 14.3 Hz, 2H, H-6'), 2.43 (s, 3H, H-21), 2.39 (d, *J* = 14.3 Hz, 2H, H-6''), 1.71 (s, 6H, H-16), 1.60 (s, 6H, H-16'), 1.21 (s, 18H, H-15'), 1.16 (s, 18H, H-15).

^{13}C NMR (151 MHz, CD_2Cl_2, 243 K)*: δ 245.4 (C-4), 159.1 (C-2), 156.2 (C-3), 153.3 (C-8), 153.1 (C-10'), 151.2 (C-10), 151.0 (C-8'), 148.3 (C-17), 139.7 (C-20), 138.4 (C-13'), 133.6 (C-13), 128.4 (C-19), 127.9 (C-11'), 126.7 (C-18), 124.3 (C-11), 122.6 (C-12'), 122.25 (C-12), 122.23 (C-9'), 120.7 (C-9), 116.7 (C-1), 64.0 (C-7), 53.1 (C-6), 45.0 (C-5), 35.2 (C-14'), 34.8 (C-14), 33.7 (C-16'), 32.3 (C-16), 31.3 (C-15), 31.2 (C-15'), 21.2 (C-21).

* NMR data are reported at 243 K due to the fluxional nature of the species resulting in broad signals at room temperature on the NMR timescale, making accurate assignment and characterization impossible.

FTIR (ATR, 25 °C, 32 scans, cm^{-1}): 2948 (m), 2899 (m), 2869 (m), 2859 (m), 1615 (m), 1474 (m), 1458 (m), 1408 (m), 1359 (m), 1252 (m), 1233 (s), 1128 (s), 1070 (s), 899 (s, br), 878 (s), 826 (s), 814 (s), 741 (s), 707 (s), 665 (s), 637 (s), 562 (s), 537 (s), 512 (s).

HRMS (ESIpos): calc'd for $C_{63}H_{73}Bi_1N_1O_2S_1^+$ [M]$^+$ 1116.51566; found 1116.51605.

M.p.: 291.7-292.7 °C.

Anal. calc'd for $C_{63}H_{72}BiNO_2S$: C, 67.78; H, 6.50; N, 1.25; Bi, 18.72; O, 2.87; S, 2.87; found C, 67.46; H, 6.41; N, 1.21; Bi, 18.59; O, -*; S, 2.84. *Oxygen determination was not possible due to chemical interference.

Stability: air-sensitive.

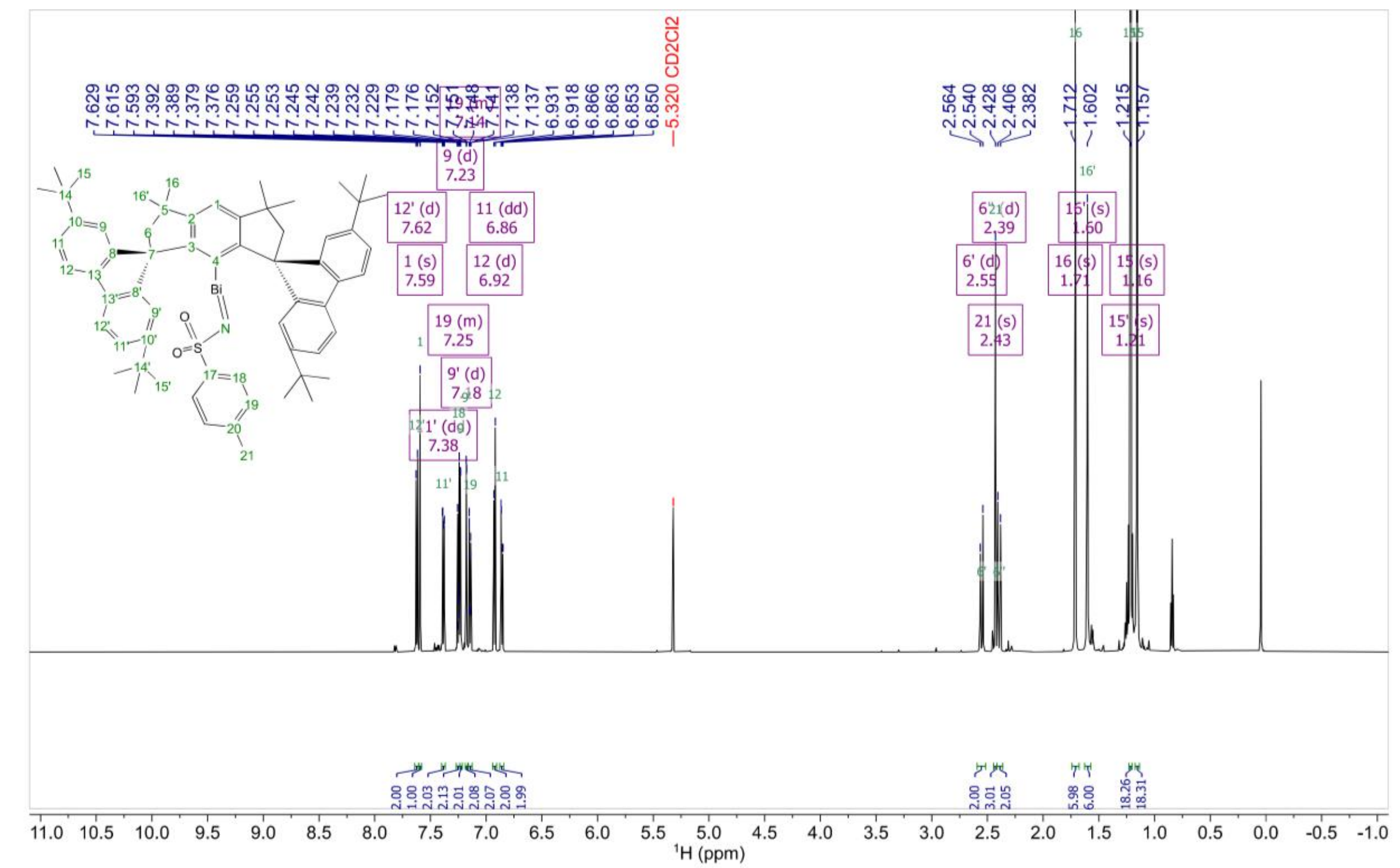

Figure S1. ^{1}H NMR spectrum of **2** (600 MHz, CD_2Cl_2, 243 K).

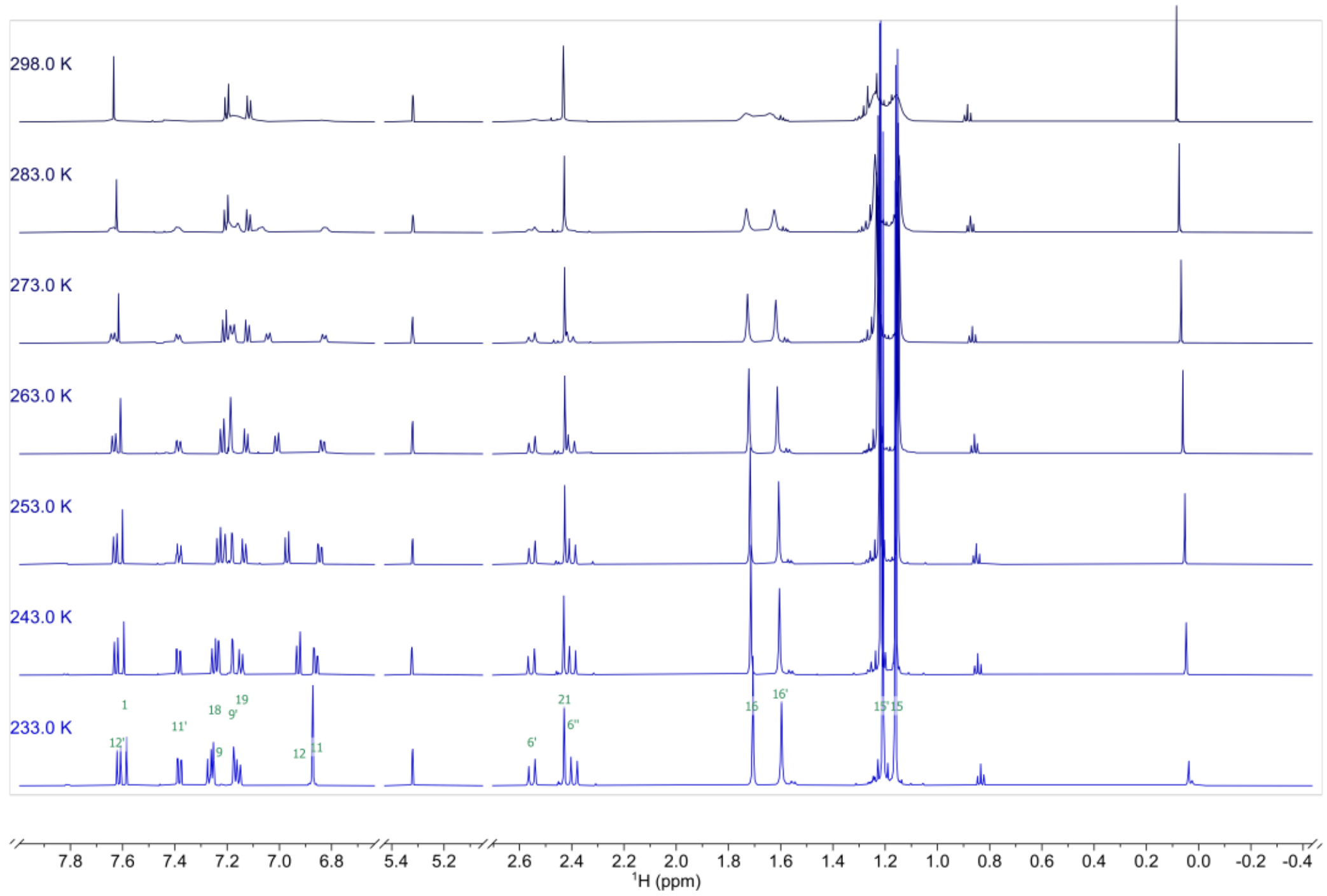

Figure S2. Stacked Variable Temperature (VT) ^{1}H NMR spectra of **2** (600 MHz, CD_2Cl_2).

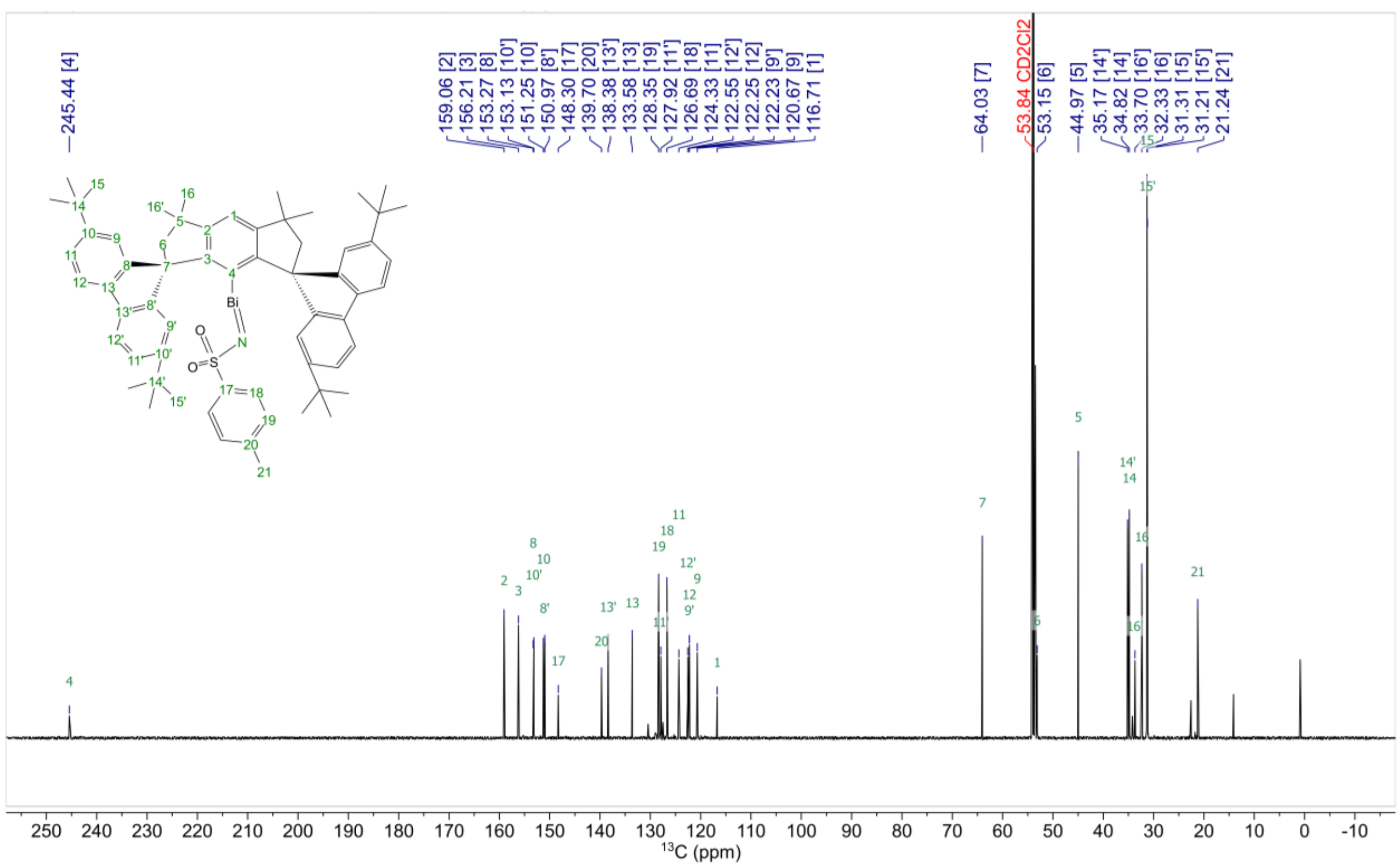

Figure S3. ^{13}C NMR spectrum of **2** (600 MHz, CD_2Cl_2, 243 K).

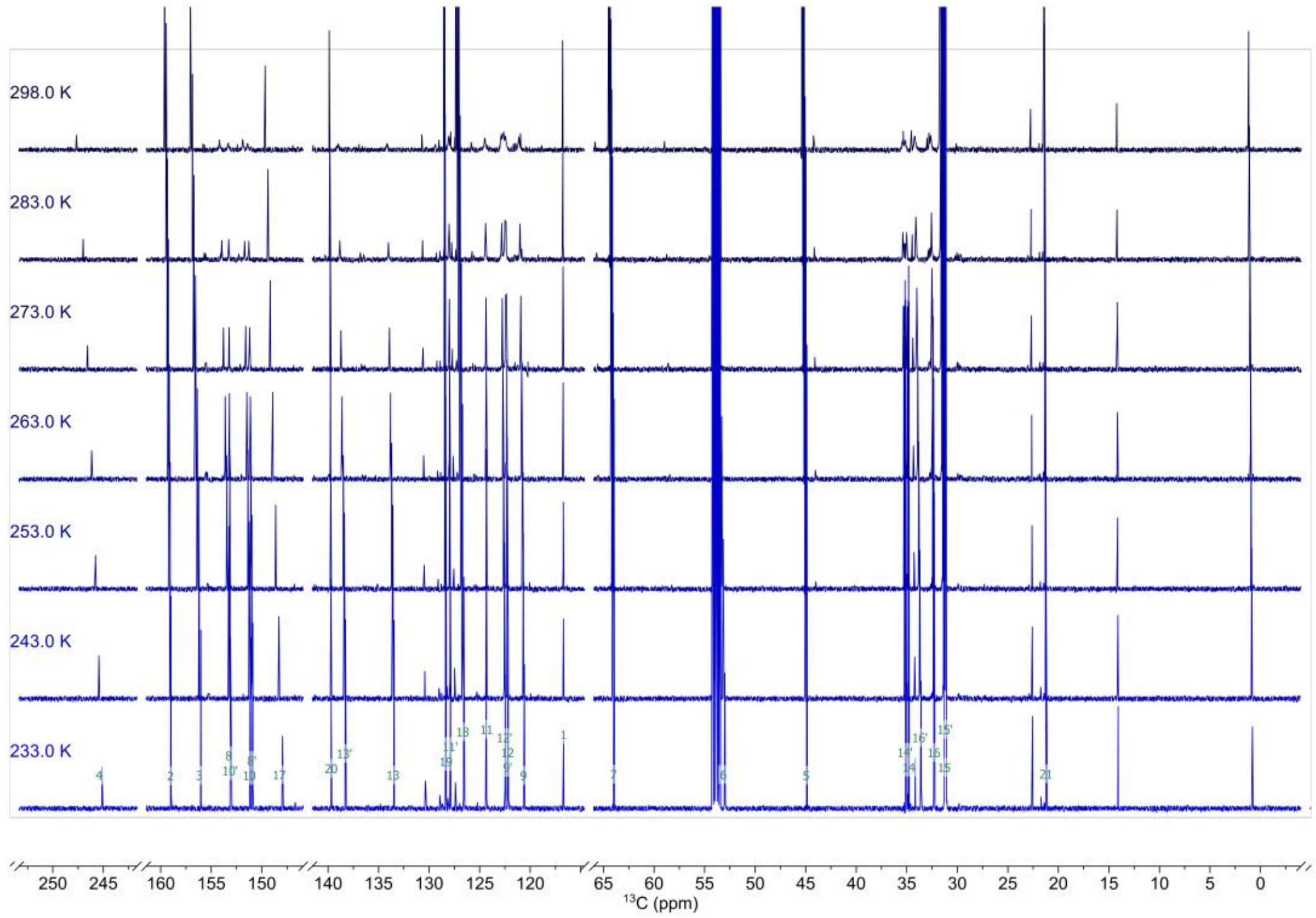

Figure S4. Stacked Variable Temperature (VT) ^{13}C NMR spectra of **2** (600 MHz, CD_2Cl_2).

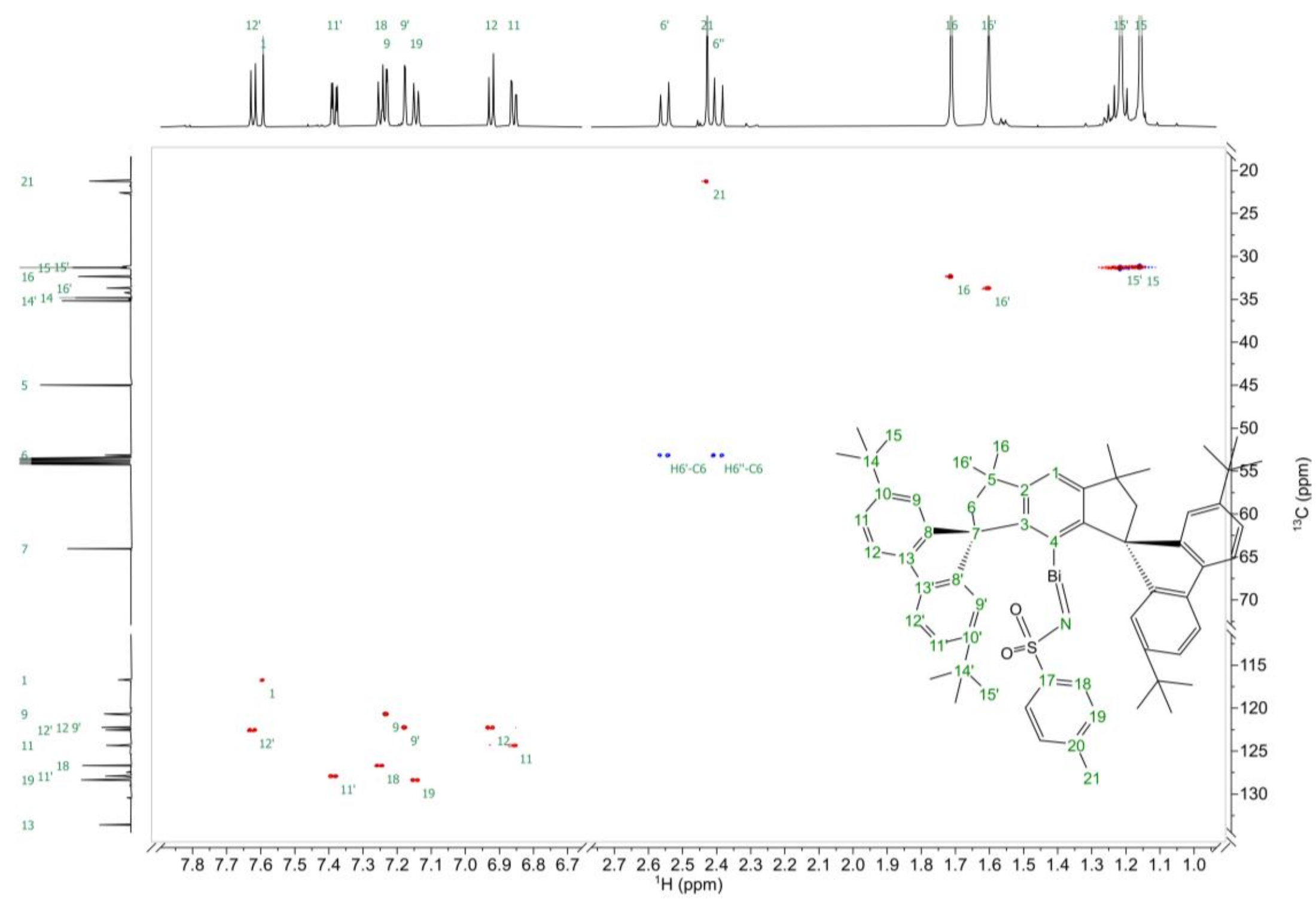

Figure S5. ^{1}H-^{13}C edited HSQC spectrum of **2** (600 MHz, CD_2Cl_2, 243 K).

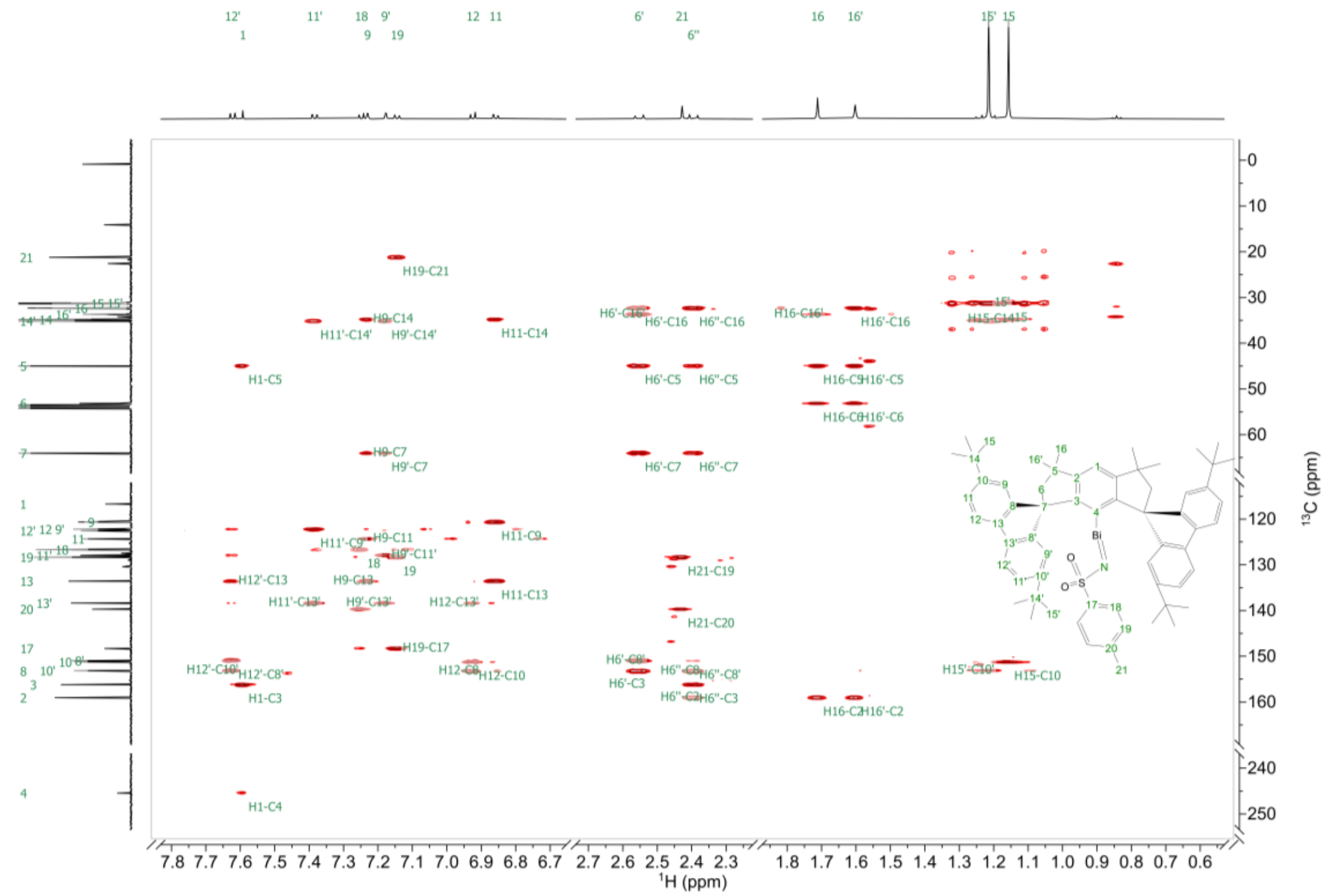

Figure S6. ^{1}H-^{13}C HMBC spectrum of **2** (600 MHz, CD_2Cl_2, 243 K).

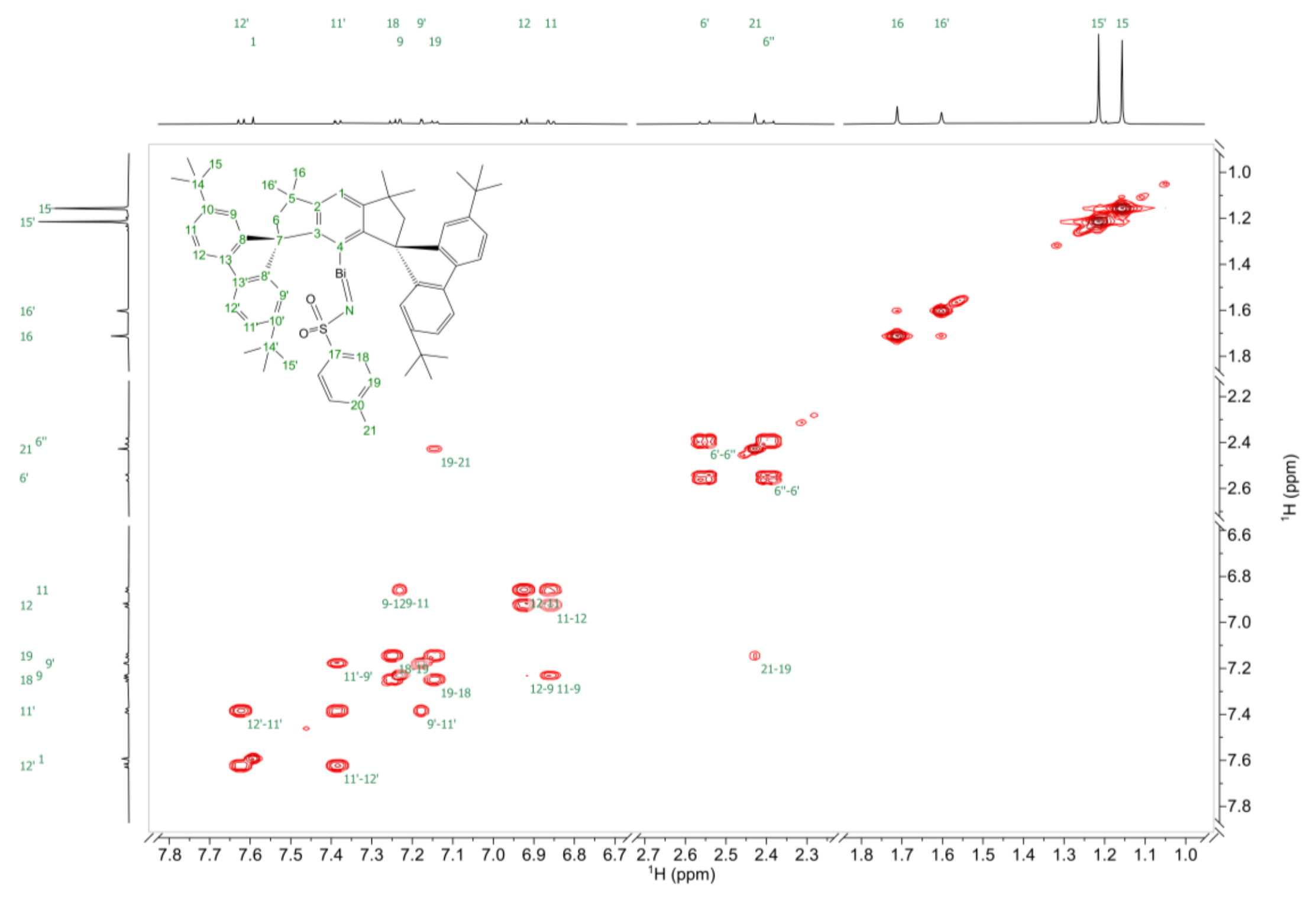

Figure S7. ^{1}H-^{1}H COSY spectrum of **2** (600 MHz, CD_2Cl_2, 243 K).

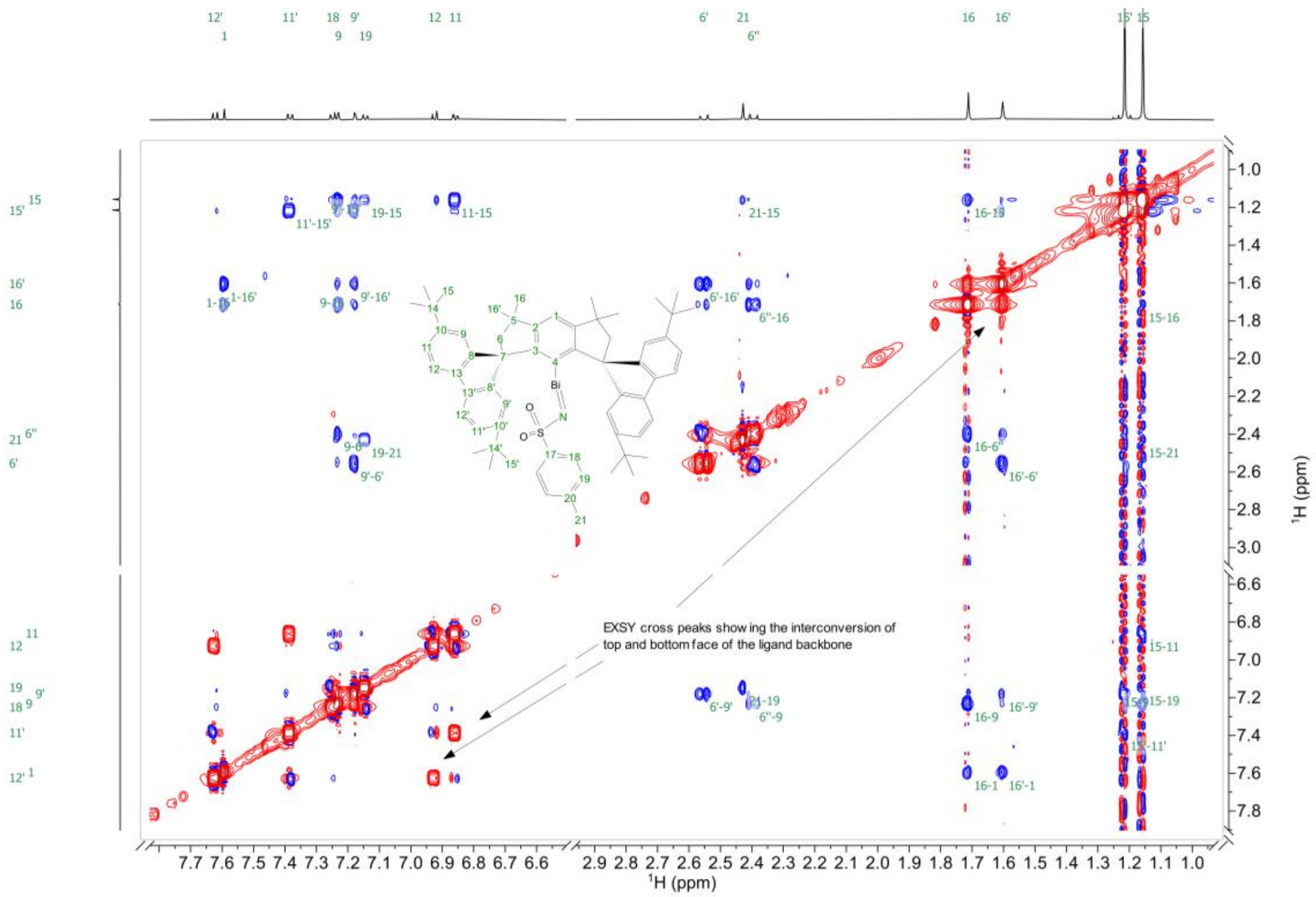

Figure S8. ^{1}H-^{1}H ROESY spectrum of **2** (600 MHz, CD_2Cl_2, 243 K).

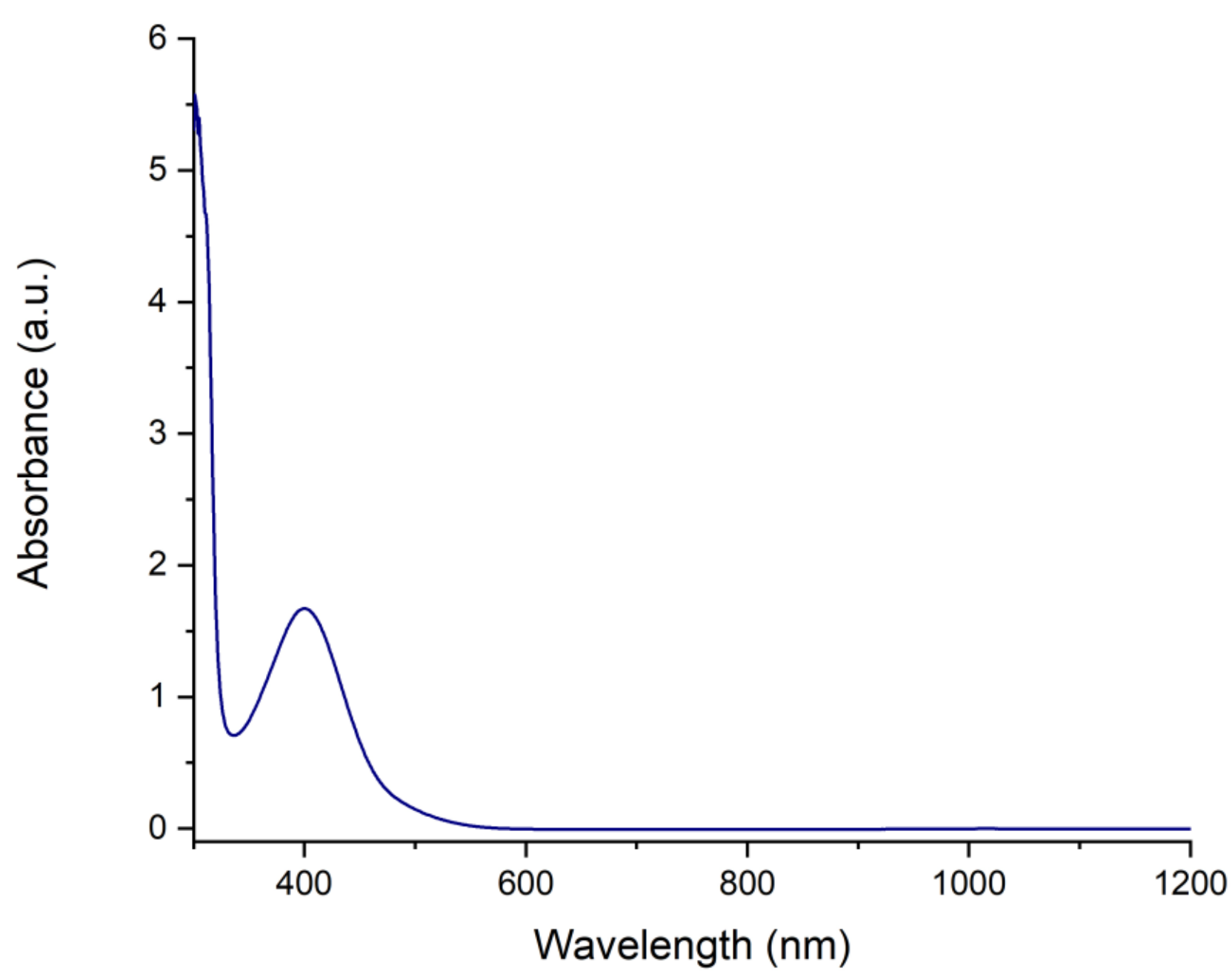

Figure S9. UV-Vis spectrum of **2** (2.0 mM) in CH_2Cl_2.

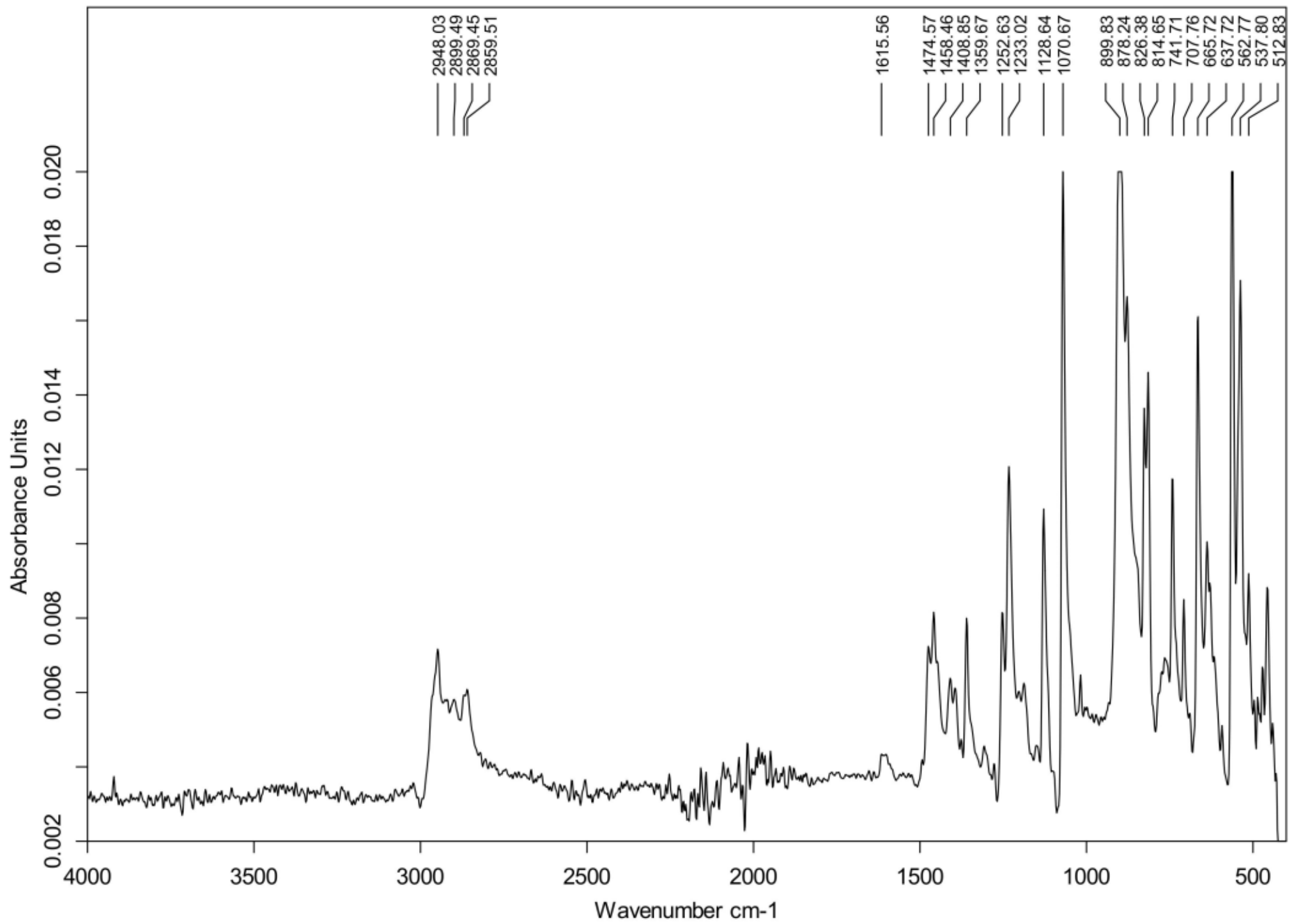

Figure S10. FTIR spectrum of **2**.

2.2. Synthesis and characterization of 3

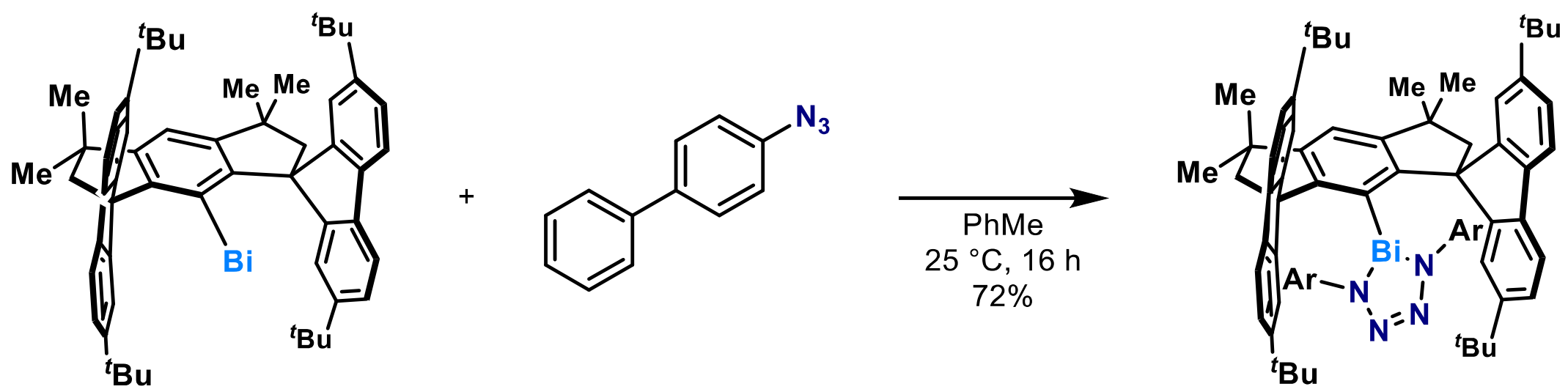

1 3

Procedure: In an argon-filled glove box, **1** (150 mg, 0.158 mmol, 1.00 equiv.) and 4-azidobiphenyl (61.8 mg, 0.317 mmol, 2.00 equiv.) were weighed in an oven-dried vial, followed by the addition of toluene (3.50 mL). After stirring at 25 °C for 16 h, the solvent was removed *in vacuo*, and pentane (5 mL) was added. The heterogeneous mixture was transferred to a glass-fritted filter (porosity IV), washed with pentane (15 mL), and the solid was dried *in vacuo* to obtain **3** as a light purple microcrystalline powder (150 mg, 72%).

Characterization data of **3**:

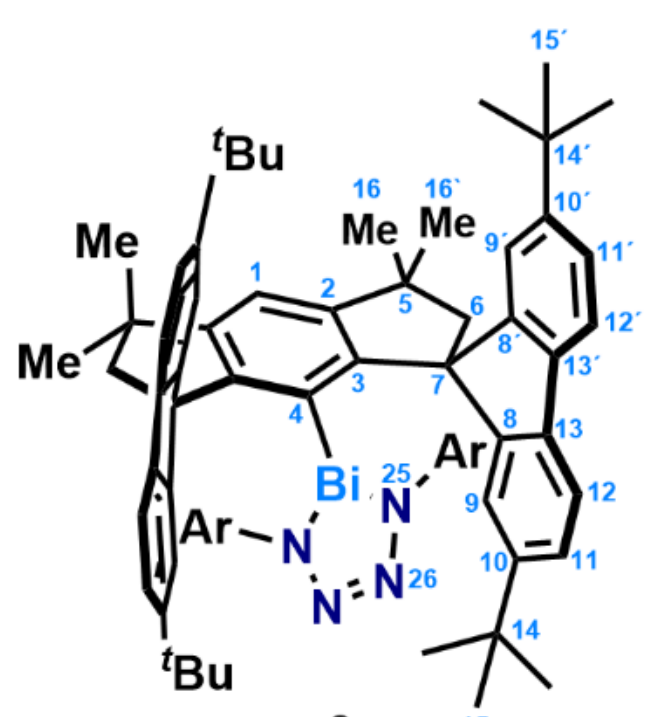

3

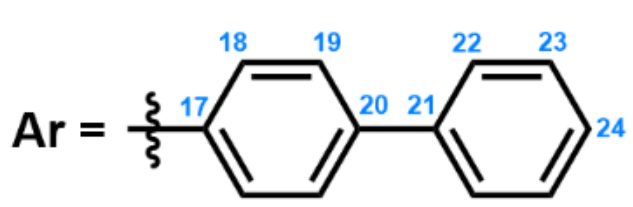

^{1}H NMR (600 MHz, CD_2Cl_2, 253 K)*: δ 7.58 (s, 1H, H-1), 7.53 (d, J = 8.0 Hz, 2H, H-12), 7.47 (d, J = 1.7 Hz, 2H, H-9), 7.44 (m, 4H, H-22), 7.43 (dd, J = 7.9, 7.9 Hz, 2H, H-12'), 7.40 (m, 4H, H-23), 7.39 (dd, J = 8.0, 1.7 Hz, 2H, H-11), 7.26 (m, 2H, H-24), 7.17 (d, J = 7.9 Hz, 4H, H-19), 6.85 (dd, J = 7.9, 1.7 Hz, 2H, H-11'), 6.78 (dd, J = 7.9, 1.7 Hz, 2H, H-9'), 6.00 (d, J = 7.9 Hz, 4H, H-18), 2.50 (s, 2H, H-6'), 2.02 (s, 2H, H-6''), 1.72 (s, 6H, H-16), 1.49 (s, 6H, H-16'), 1.25 (s, 18H, H-15), 0.81 (s, 18H, H-15').

^{13}C NMR (151 MHz, CD_2Cl_2, 253 K)*: δ 208.3 (C-4), 161.5 (C-2'), 158.0 (C-2), 156.0 (C-8), 154.0 (C-8'), 154.0 (C-3), 152.6 (C-3'), 152.5 (C-10), 149.5 (C-10'), 149.4 (C-17), 141.0 (C-21), 137.1 (C-13'), 136.1 (C-13), 132.5 (C-20), 128.8 (C-23), 127.8 (C-19), 126.5 (C-11), 126.4 (C-22), 126.3 (C-24), 123.9 (C-11'), 121.9 (C-9), 120.9 (C-12), 119.8 (C-1), 119.4 (C-9'), 119.3 (C-12'), 118.4 (C-18), 66.7 (C-7'), 66.0 (C-7), 62.2 (C-6'), 58.7 (C-6), 44.3 (C-5), 42.6 (C-5'), 35.2 (C-14), 34.3 (C-14'), 33.1 (C-16), 32.8 (C-16'), 31.3 (C-15), 31.1 (C-15').

^{15}N NMR (61 MHz, CD_2Cl_2, 298 K):** δ -151.7 (N-25).

* ^{1}H and ^{13}C NMR data are reported at 253 K due to the extremely fluxional nature of the species resulting in broad signals at room temperature on the NMR timescale, which made accurate assignment and characterization impossible.

** ^{1}H-^{15}N HMBC NMR data was initially measured at 253 K, but no cross peaks were observed. Remeasurement at 298 K allowed for observation of a single cross peak, correpsonding to the ^{3}J coupling of H-18 and N-25. However, although a series of different spectra optimized to different $^{n}J_{N\text{-}H}$ values (1, 3, and 5 Hz), was acquired, the other N correlation (N-26) was not observed.

FTIR (ATR, 25 °C, 32 scans, cm^{-1}): 3059 (w), 3029 (m), 2295 (m), 2901 (m), 2866 (m), 1597 (s), 1511 (m), 1480 (s), 1411 (m), 1393 (m), 1281 (s), 1251 (s), 1178 (m), 1154 (s), 1123 (m), 980 (m), 932 (s), 887 (m), 819 (s), 761 (s), 698 (s), 640 (m), 611 (m), 543 (m), 494 (s), 466 (m).

HRMS (ESIpos): Compound decomposed.

M.p.: 179.1-185.7 °C (decomposition).

Anal. calc'd for $C_{80}H_{83}BiN_4$: C, 73.37; H, 6.39; N, 4.28; Bi, 15.96; found C, 73.14; H, 6.41; N, 4.26; Bi, 15.91.

Stability: air-stable, light-sensitive in solid state. Highly oxygen sensitive when in solution.

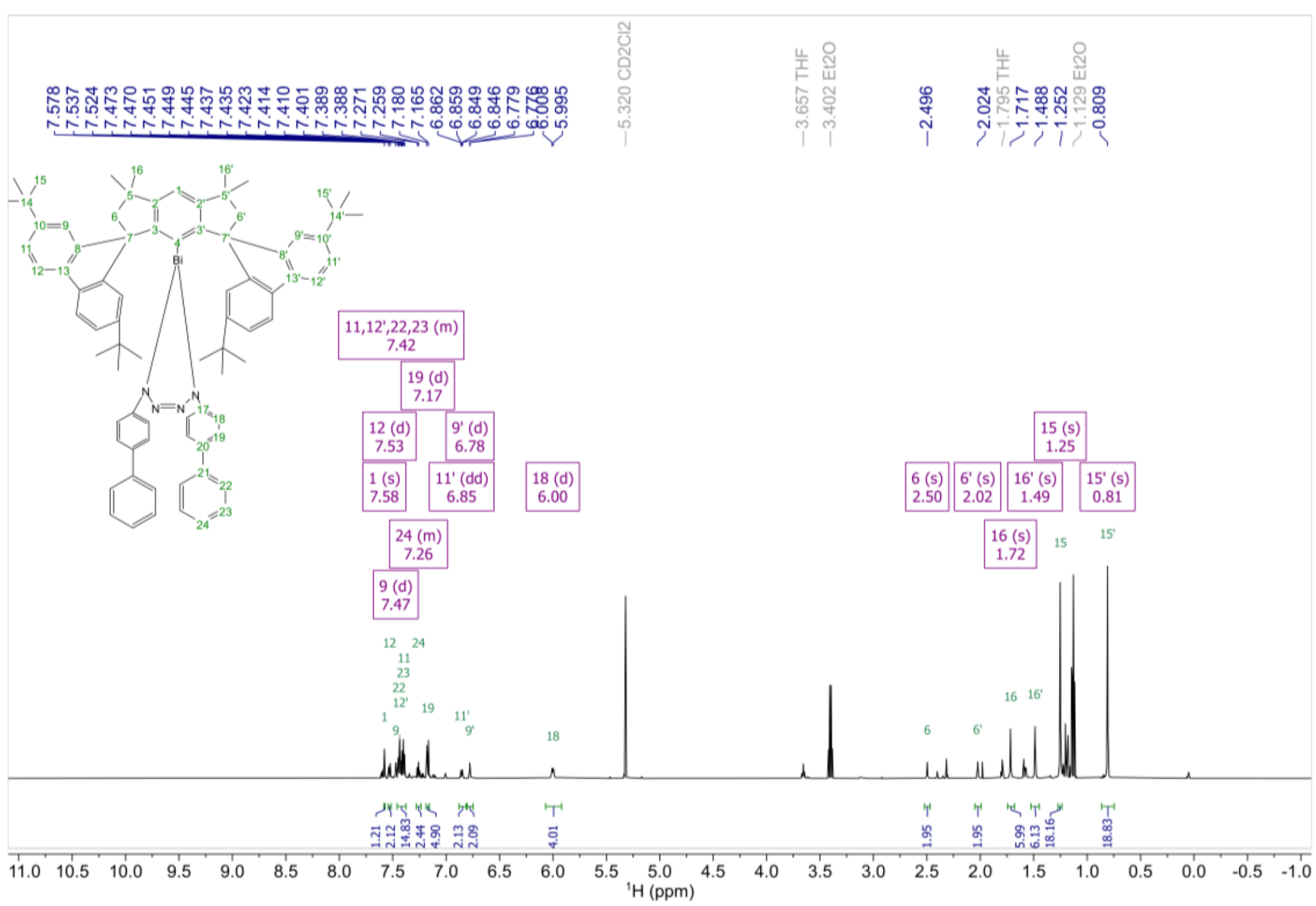

Figure S11. ^{1}H NMR spectrum of **3** (600 MHz, CD_2Cl_2, 253 K).

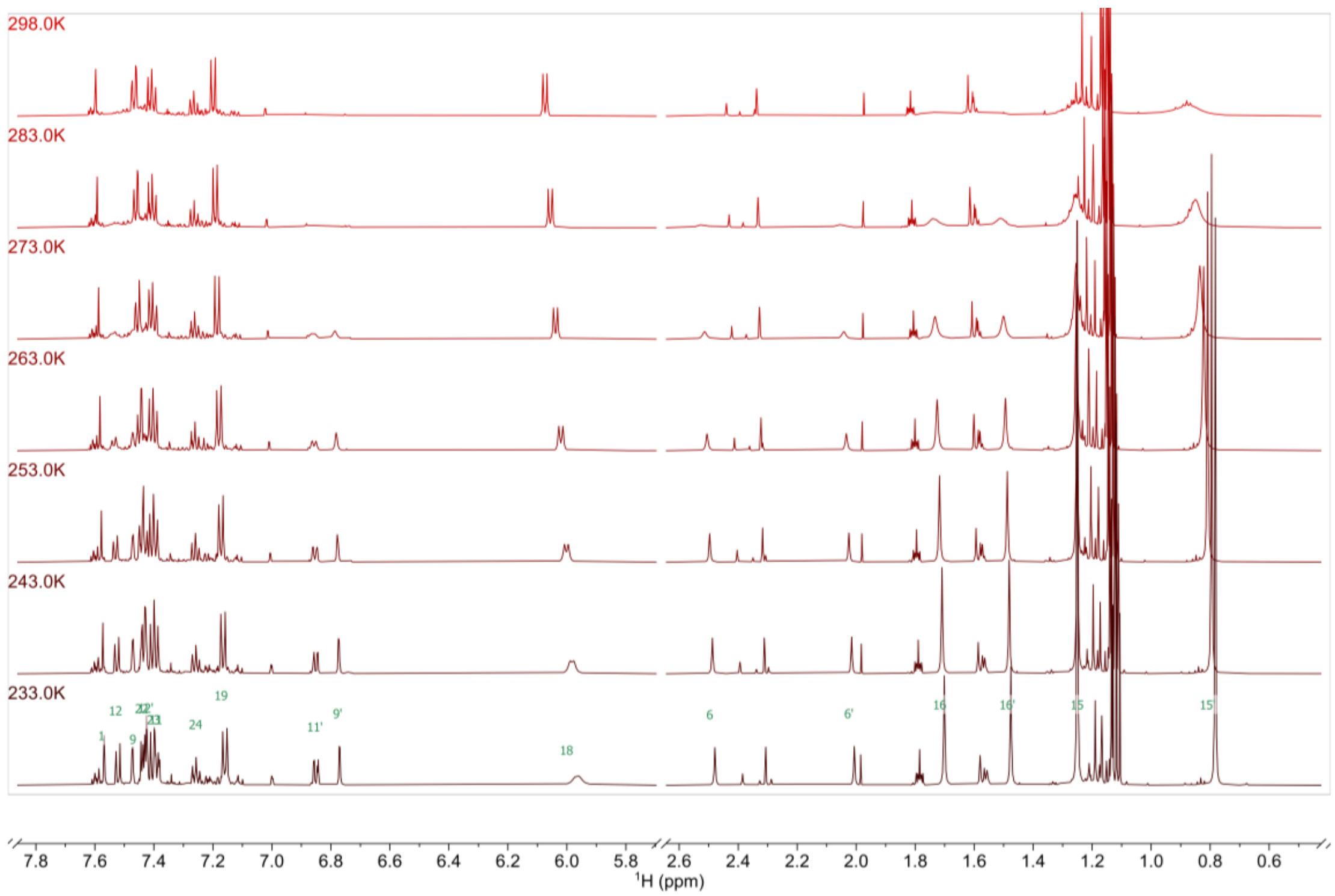

Figure S12. Stacked Variable Temperature (VT) ^{1}H NMR spectra of **3** (600 MHz, CD_2Cl_2).

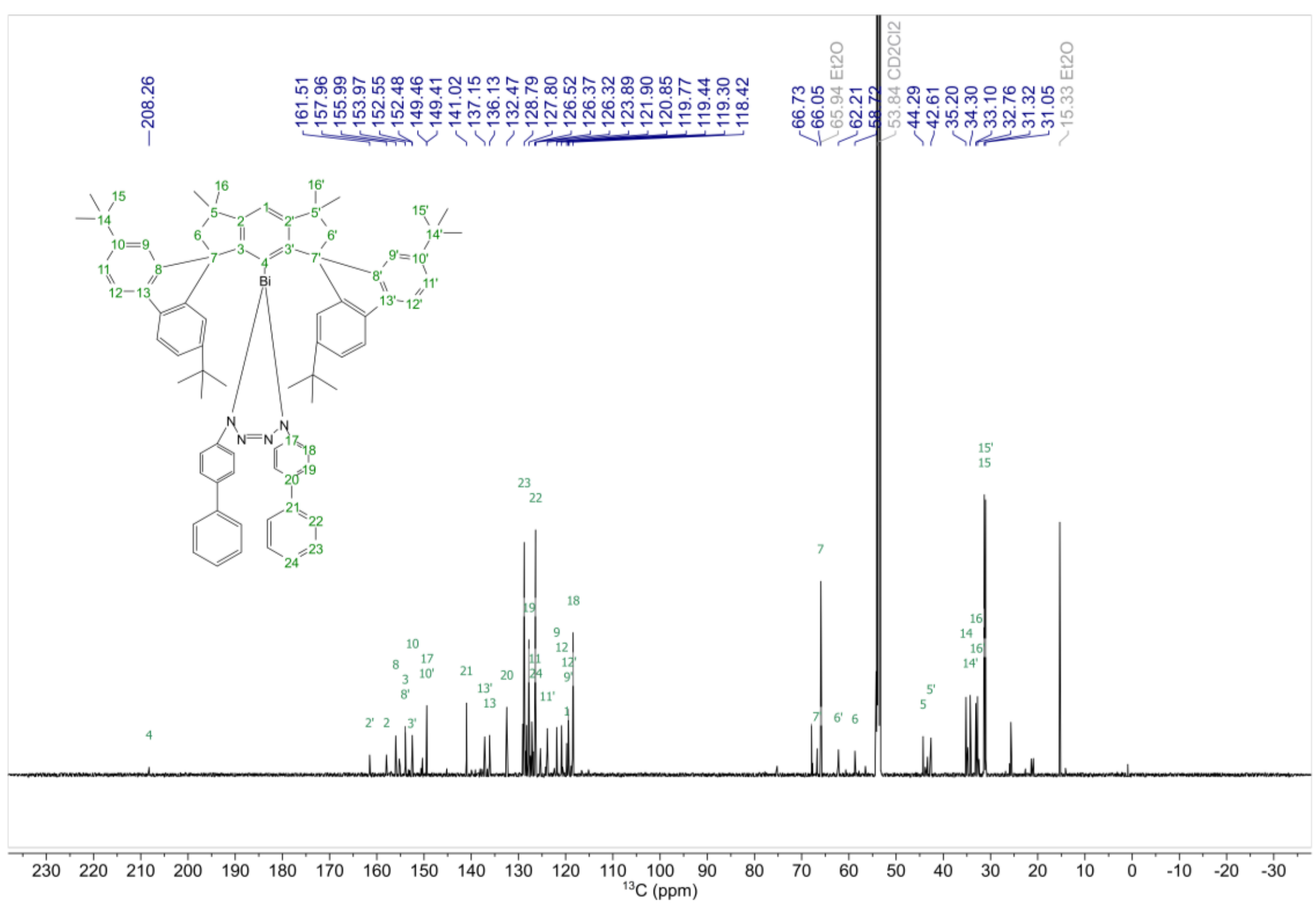

Figure S13. ^{13}C NMR spectrum of **3** (150 MHz, CD_2Cl_2, 253 K).

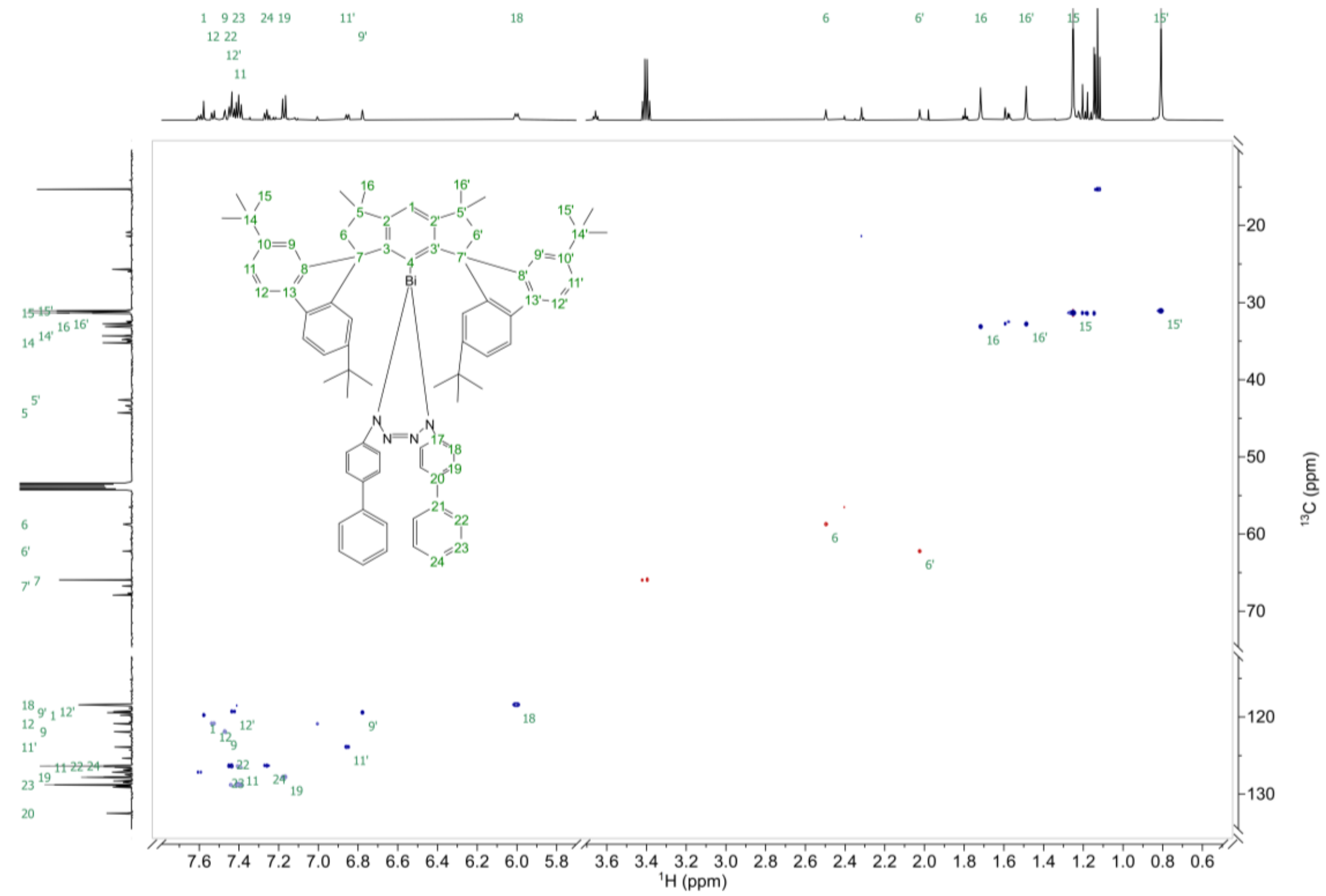

Figure S14. ^1H-^{13}C edited HSQC spectrum of **3** (600 MHz, CD_2Cl_2, 253 K).

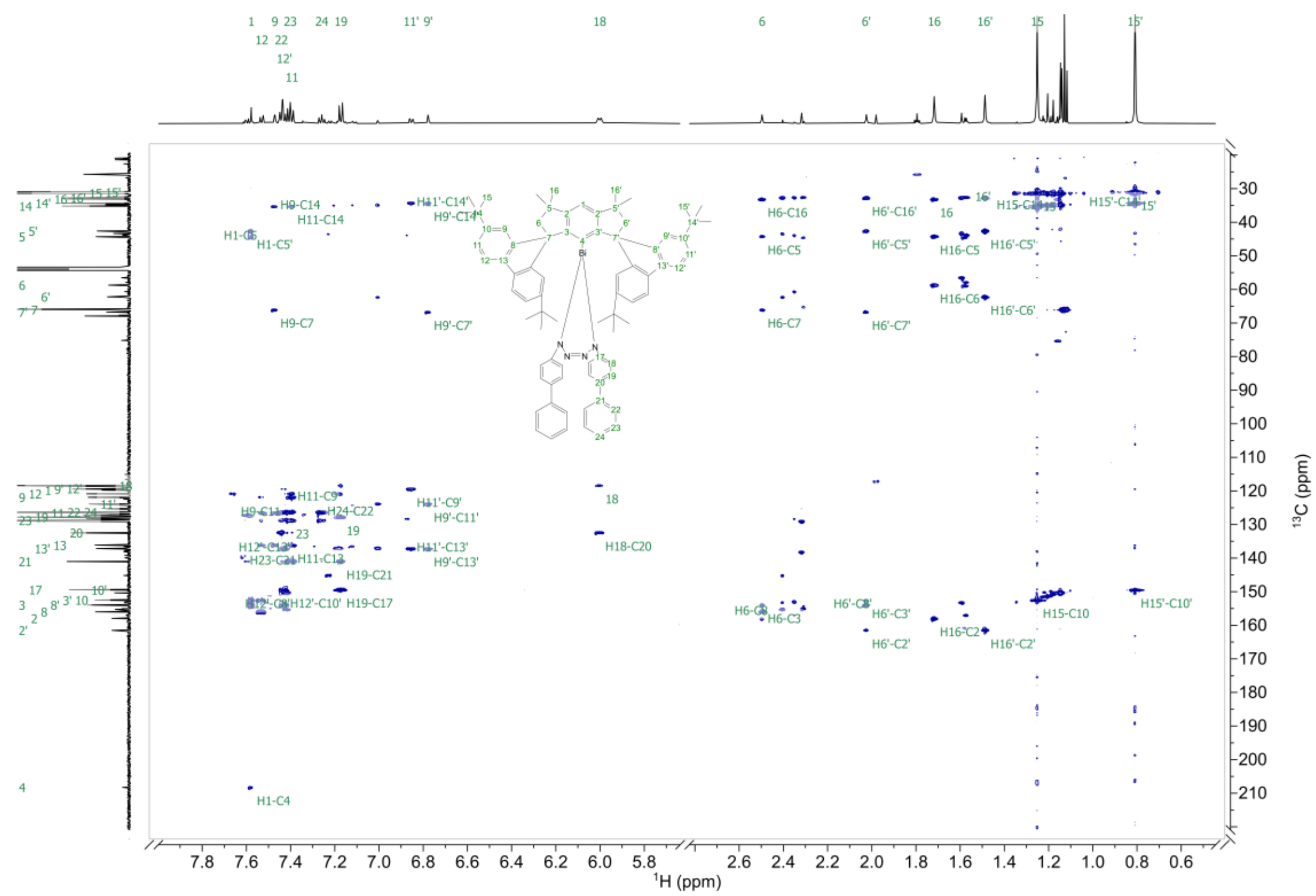

Figure S15. ^1H-^{13}C HMBC spectrum of **3** (600 MHz, CD_2Cl_2, 253 K).

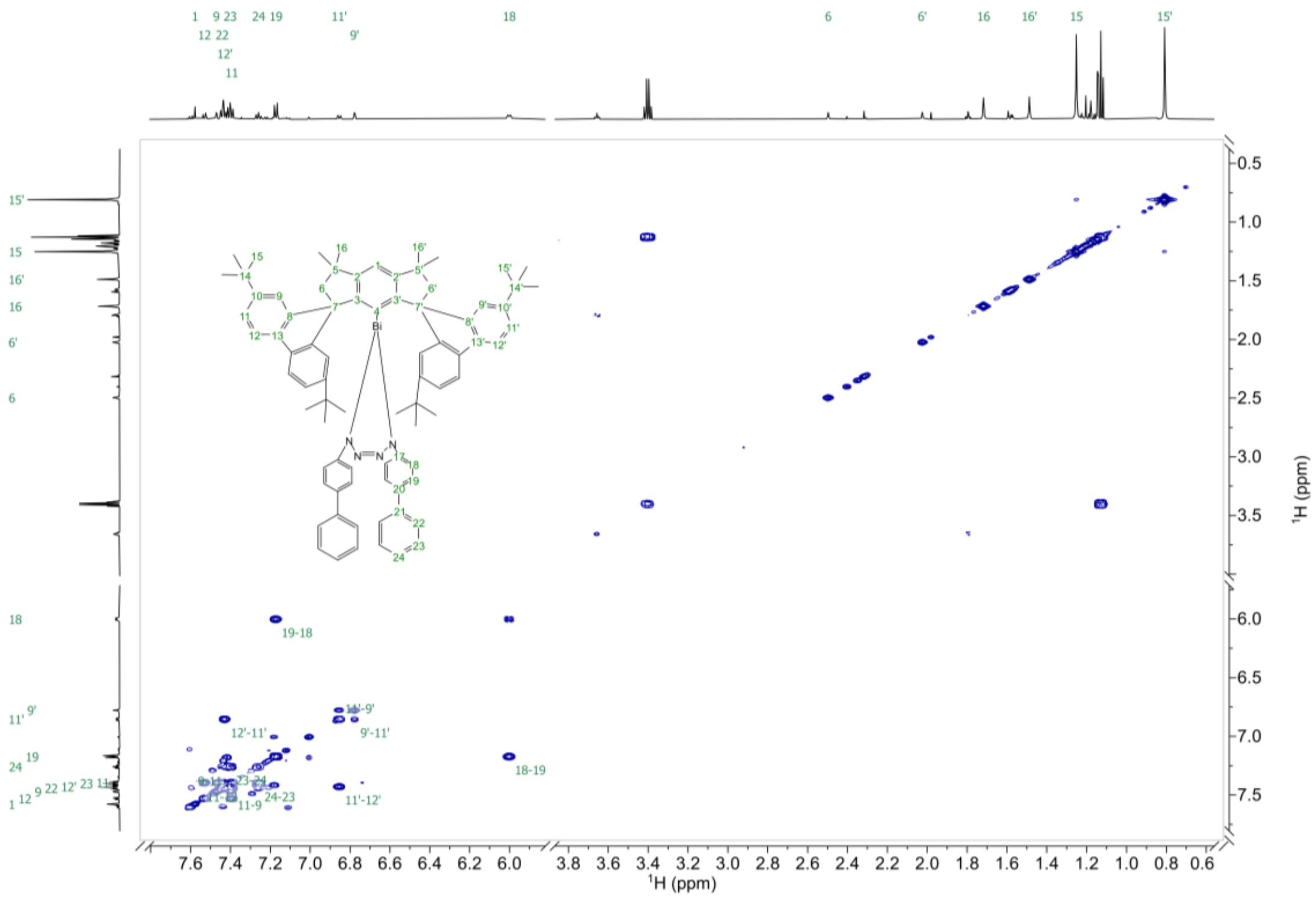

Figure S16. ^{1}H-^{1}H COSY spectrum of **3** (600 MHz, CD_2Cl_2, 253 K).

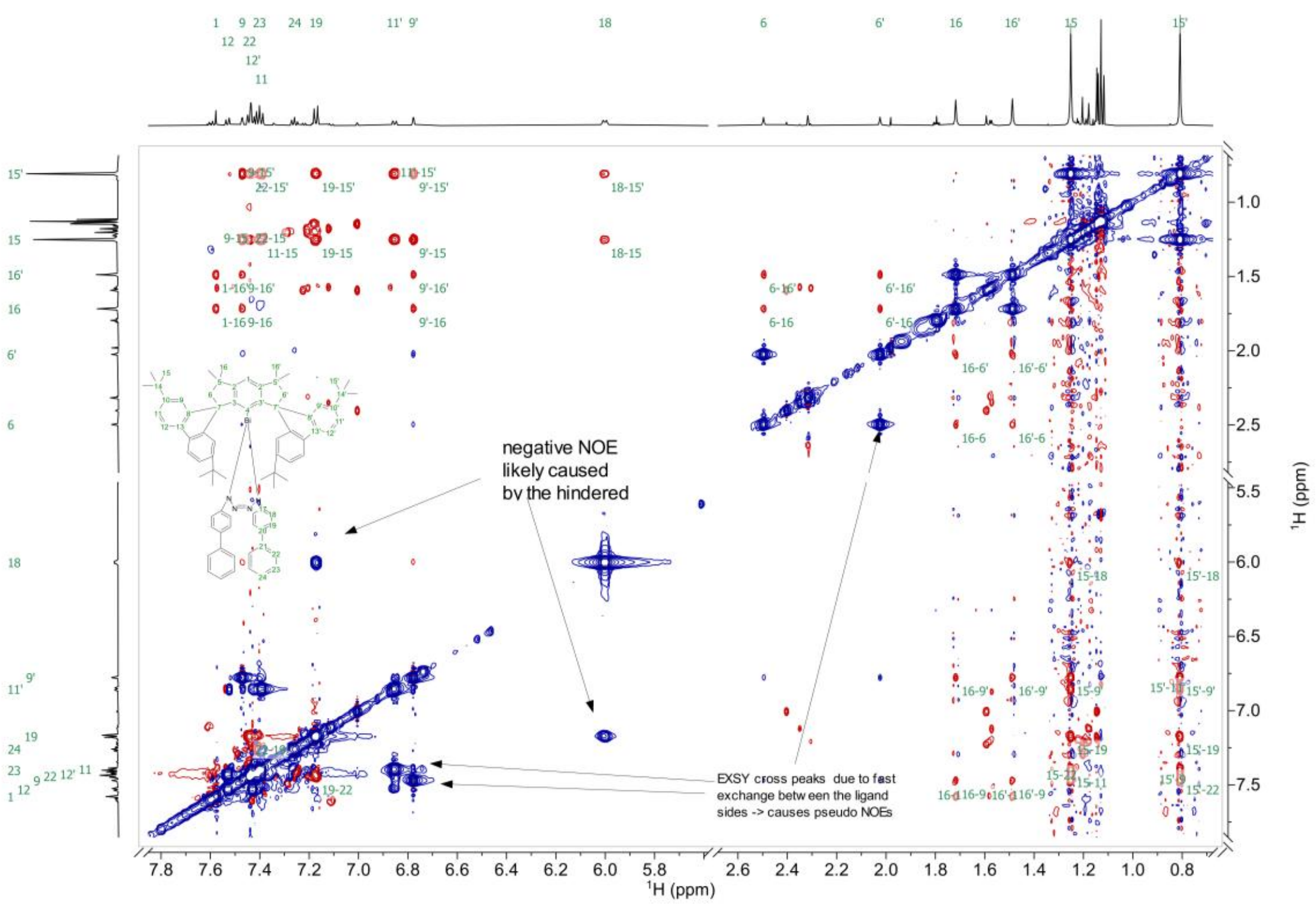

Figure S17. ^{1}H-^{1}H NOESY spectrum of **3** (600 MHz, CD_2Cl_2, 253 K).

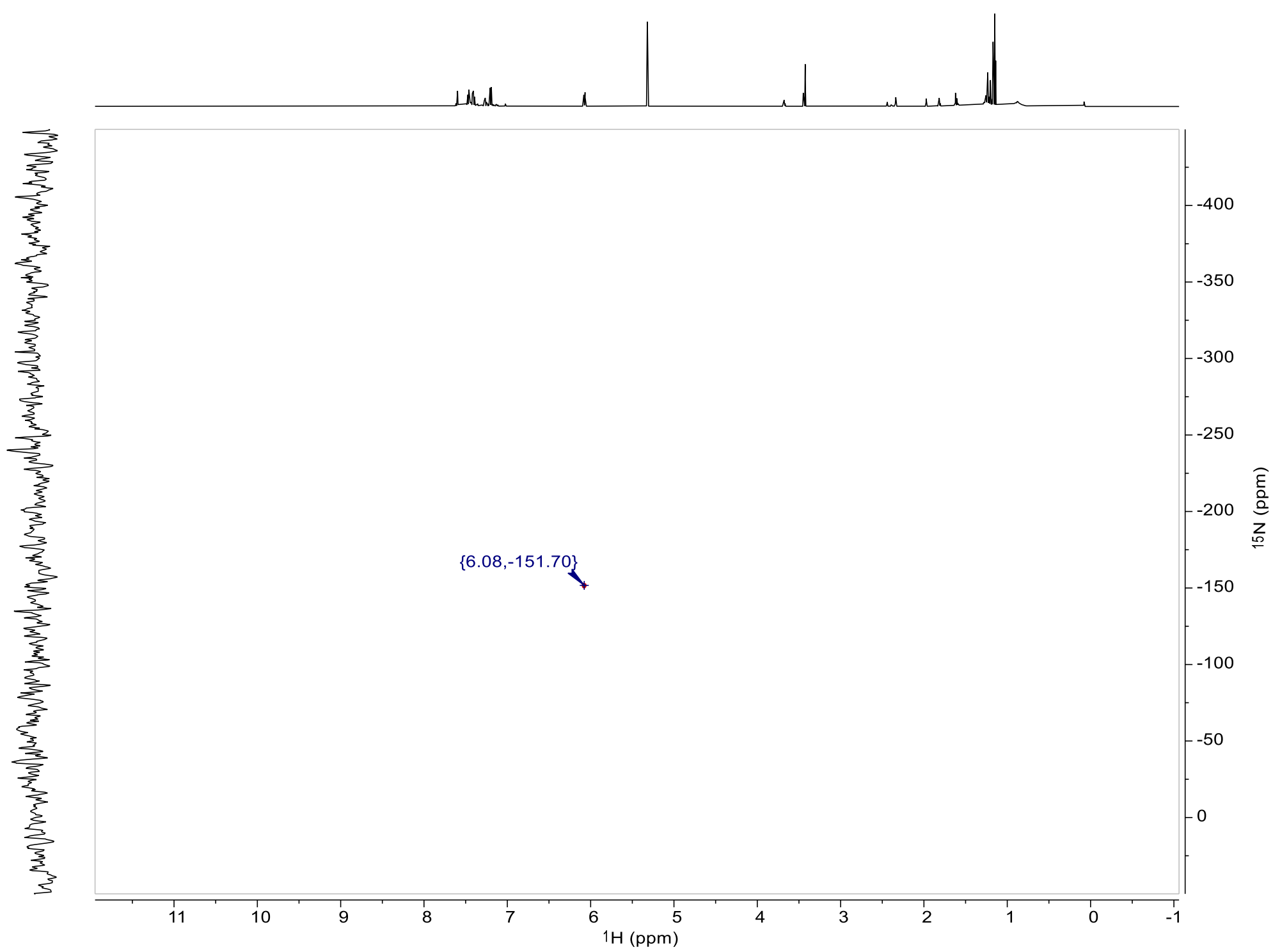

Figure S18. ^{1}H-^{15}N HMBC spectrum of **3** (61 MHz, CD_2Cl_2, 298 K).

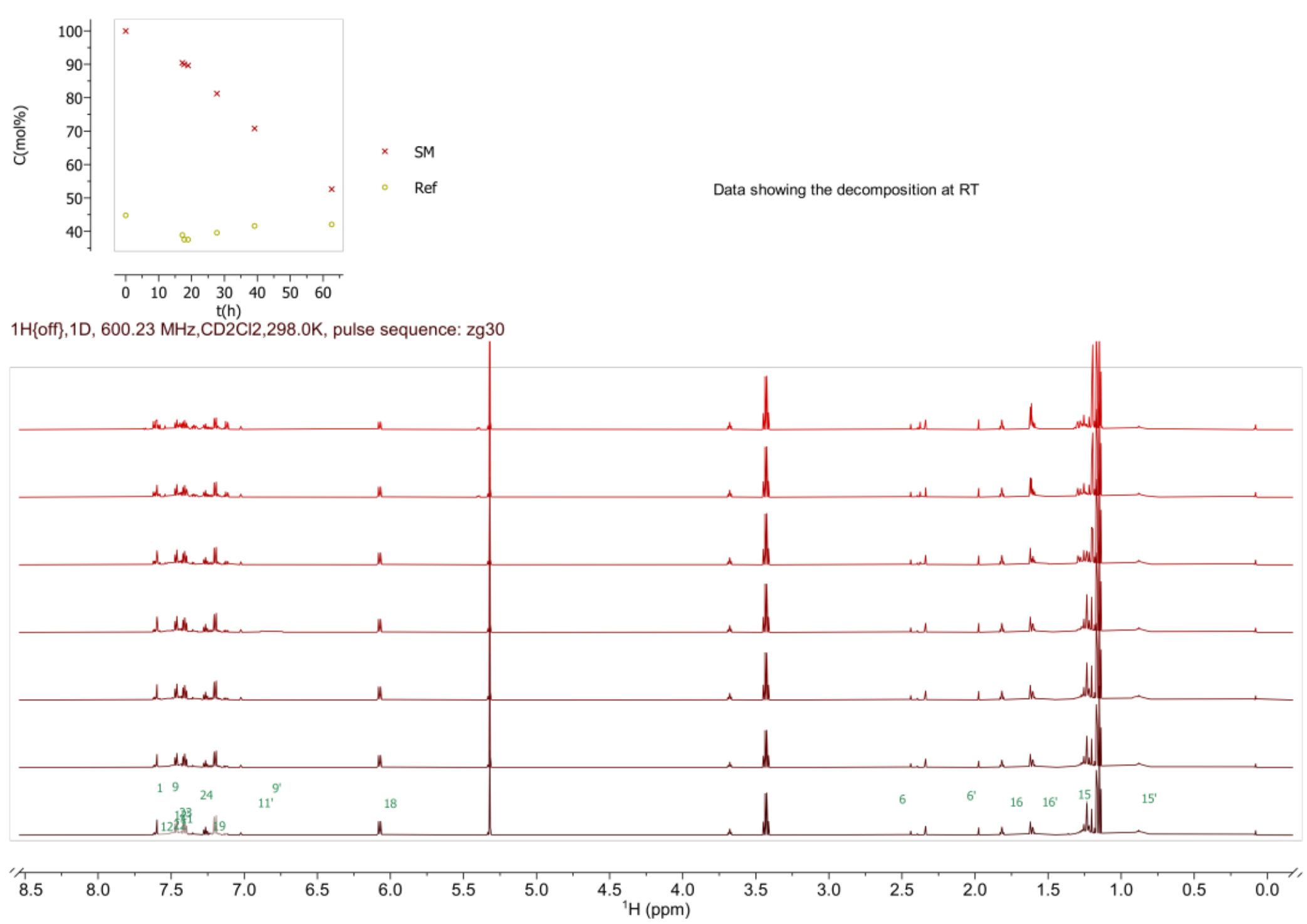

Figure S19. Observed decomposition of **3** in solution (600 MHz, CD_2Cl_2, 298 K).

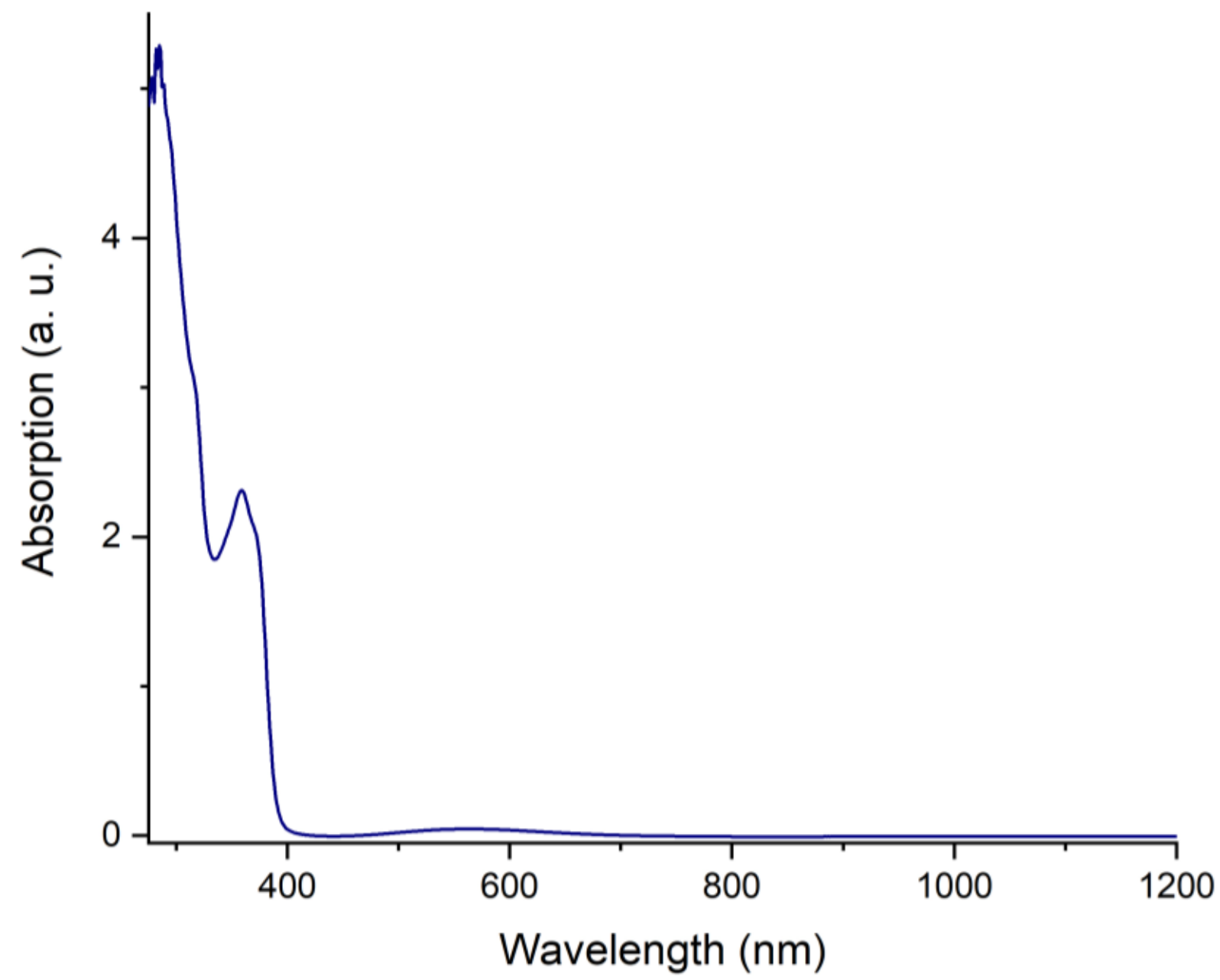

Figure S20. UV-Vis spectrum of **3** (0.24 mM) in CH_2Cl_2.

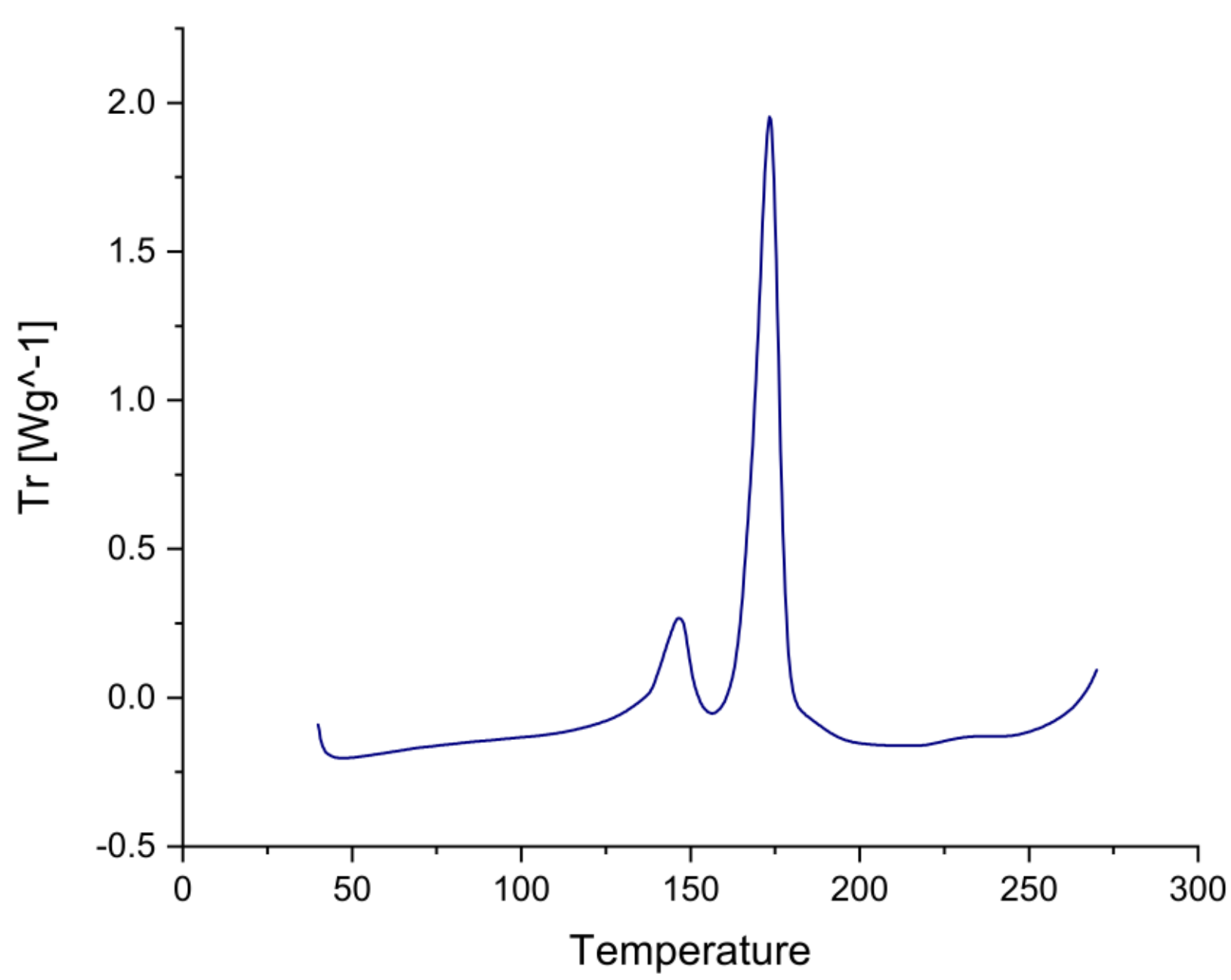

Figure S21. DSC thermogram of **3**.

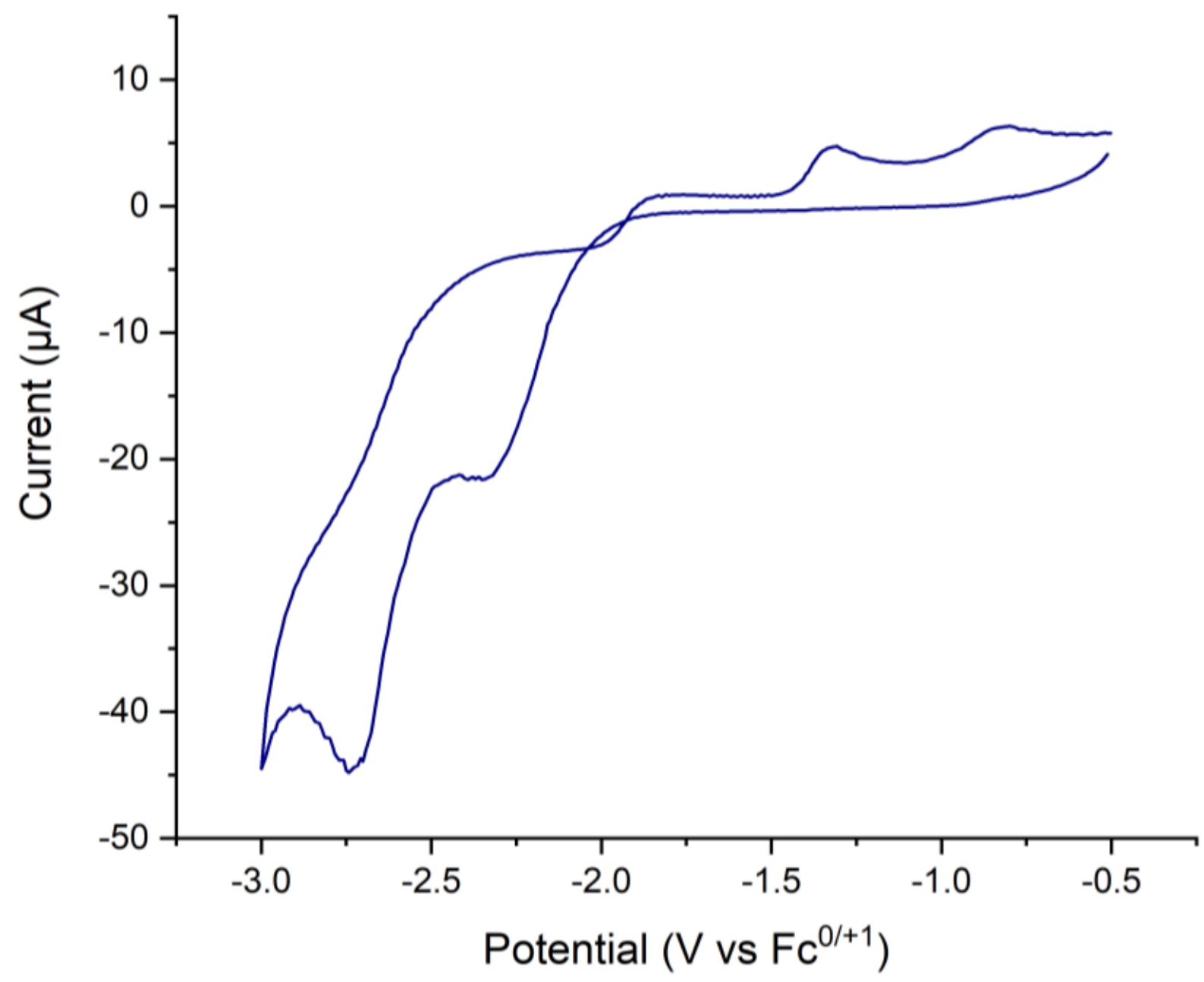

Figure S22. Cyclic voltammogram of **3** (cathodic scan) in THF using 0.1 M [nBu_4N][BArF] as supporting electrolyte at ambient temperature; scan rate: 100 mV/s, referenced to $Fc^{0/+}$.

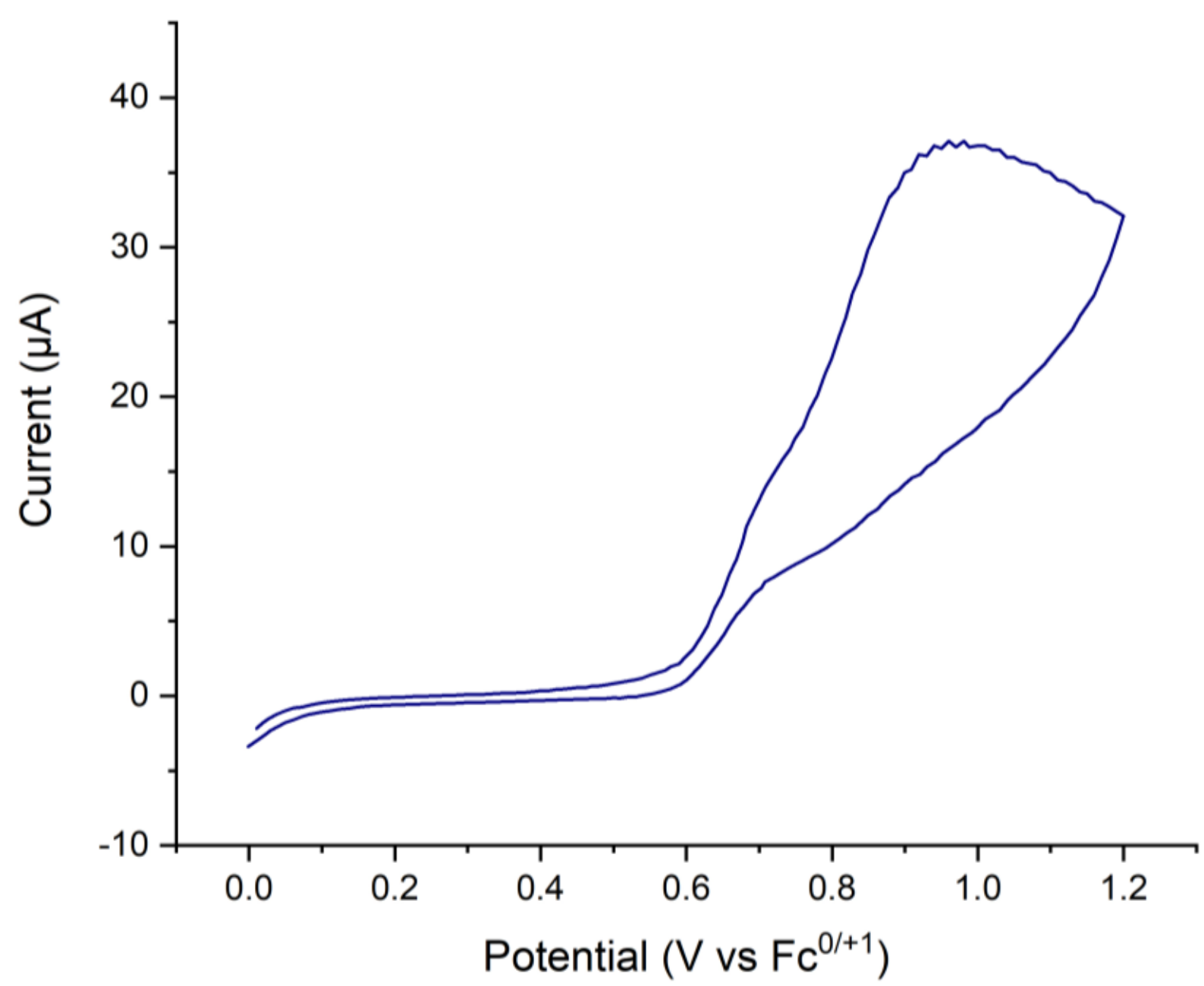

Figure S23. Cyclic voltammogram of **3** (anodic scan) in THF using 0.1 M [nBu_4N][BArF] as supporting electrolyte at ambient temperature; scan rate: 100 mV/s, referenced to $Fc^{0/+}$.

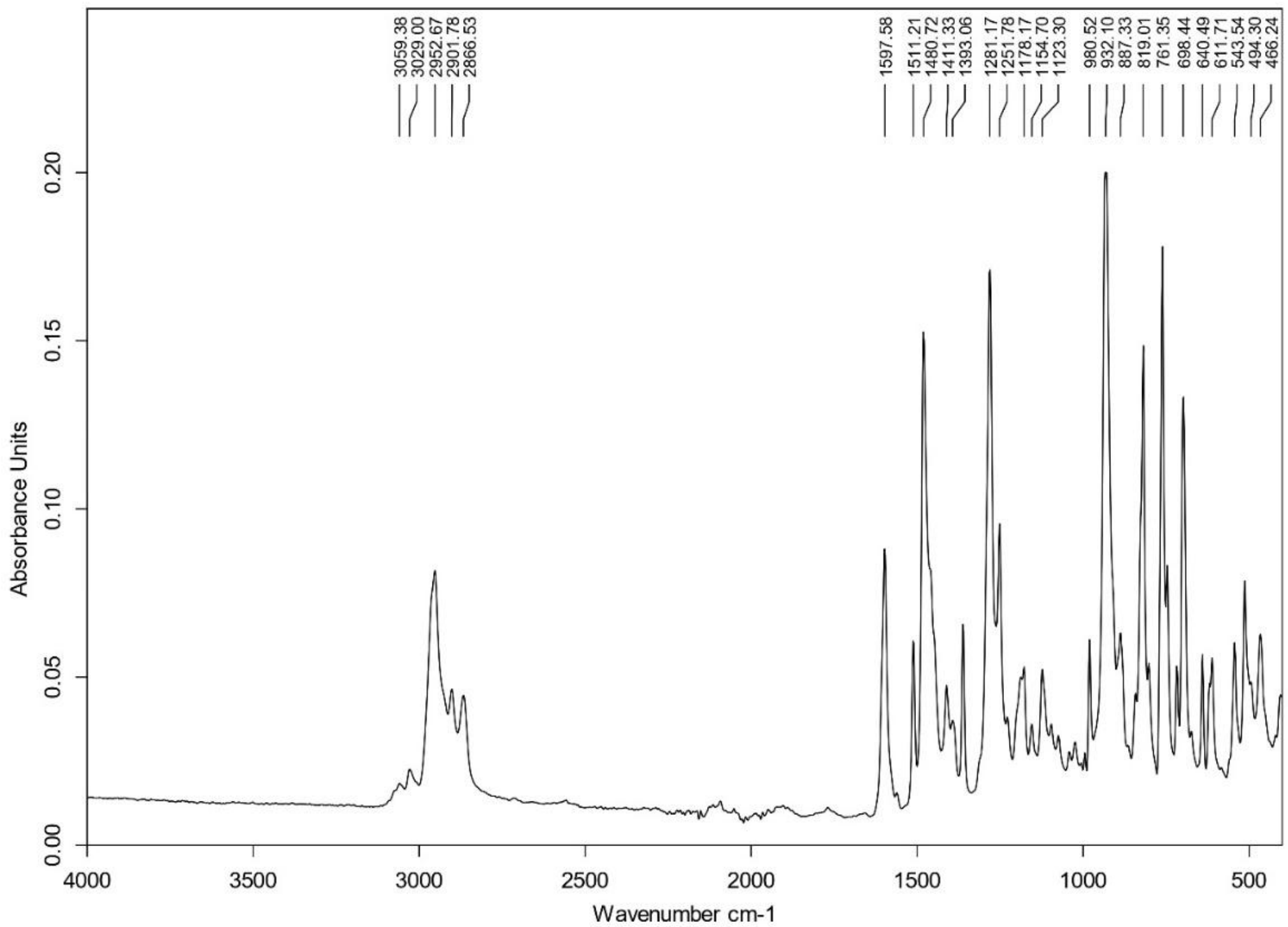

Figure S24. FTIR spectrum of **3**.

2.3. Synthesis and characterization of 4

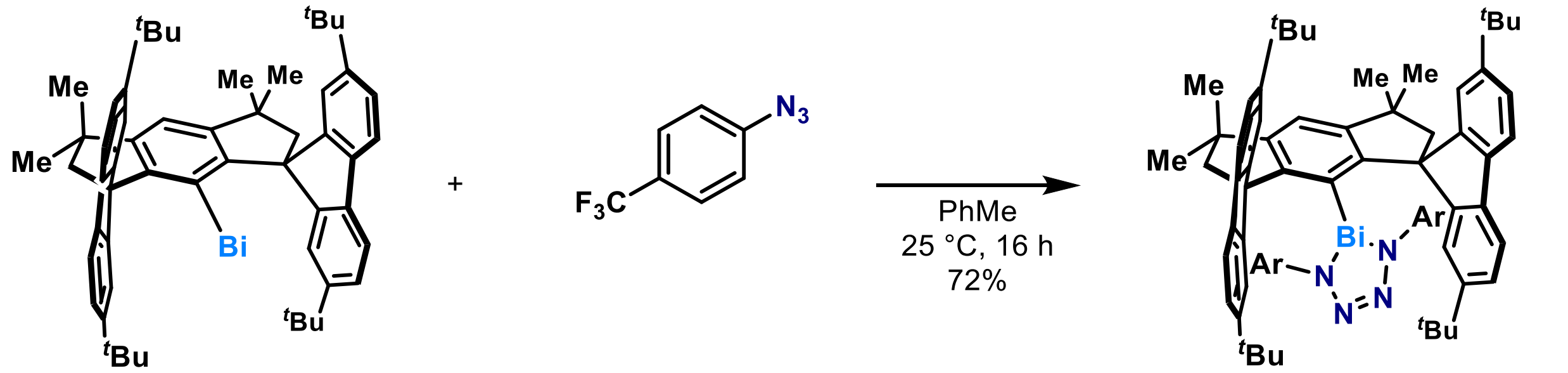

1 4

Procedure: In an argon-filled glove box, **1** (75.0 mg, 0.0792 mmol, 1.00 equiv.) and 1-azido-4-(trifluoromethyl)benzene (37.0 mg, 0.198 mmol, 2.50 equiv.) were weighed in an oven-dried vial, followed by the addition of toluene (5 mL). After stirring at 25 °C for 16 h, the solvent was removed *in vacuo*, and pentane (5 mL) was added. The heterogeneous mixture was transferred to a glass-fritted filter (porosity IV), washed with pentane (15 mL), and the solid was dried *in vacuo* to obtain **3** as a light purple microcrystalline powder (80.0 mg, 78%).

Characterization data of 4:

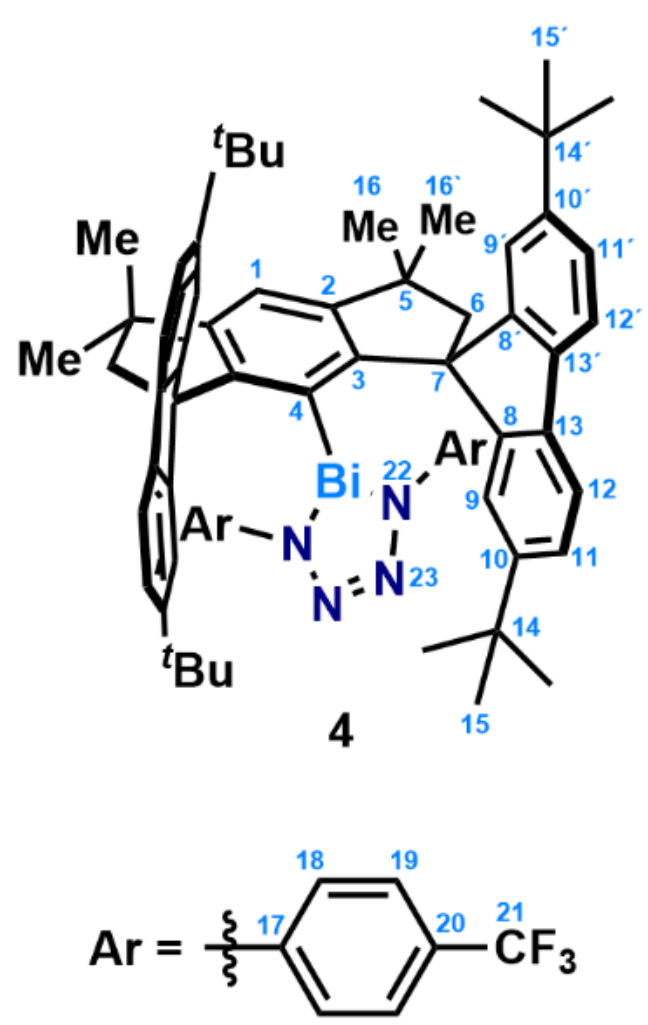

^{1}H NMR (600 MHz, CD_2Cl_2, 253 K): δ(ppm): 7.60 (s, 1H, H-1), 7.53 (d, *J* = 8.0 Hz, 2H, H-12), 7.44 (s, 2H, H-9), 7.39 (m, 4H, H-12`,11), 7.16 (d, *J* = 7.9 Hz, 4H, H-19), 6.84 (d, *J* = 8.0 Hz, 2H, H-11`), 6.75 (s, 2H, H-9`), 5.98 (bd, *J* = 7.9 Hz, 4H, H-18), 2.48 (s, 2H, H-6'), 2.02 (s, 2H, H-6''), 1.71 (s, 6H, H-16), 1.49 (s, 6H, H-16'), 1.21 (s, 18H, H-15), 0.82 (s, 18H, H-15').

^{13}C NMR (151 MHz, CD_2Cl_2, 253 K): δ(ppm): 209.8 (C-4), 161.9 (C-2'), 158.3 (C-2), 155.9 (C-8), 154.3 (C-3), 153.9 (C-8'), 153.1 (C-3'), 152.8 (C-10), 152.8 (C-17), 149.6 (C-10'), 137.1 (C-13'), 136.0 (C-13), 126.8 (C-11), 126.6 (C-19), 124.7 (C-21), 123.9 (C-11'), 121.9 (C-9), 120.9 (C-12), 120.9 (C-20), 120.3 (C-1), 119.5 (C-9'), 119.4 (C-12'), 117.6 (C-18), 66.7 (C-7'), 65.8 (C-7), 62.1 (C-6'), 58.7 (C-6), 44.5 (C-5), 42.7 (C-5'), 35.2 (C-14), 34.3 (C-14'), 33.1 (C-16), 32.7 (C-16'), 31.2 (C-15), 31.0 (C-15').

^{19}F NMR (565 MHz, CD_2Cl_2, 253 K): δ(ppm): -61.4 (CF_3).

^{15}N NMR (61 MHz, CD_2Cl_2, 298 K):** δ -151.2 (N-22).

* ^{1}H and ^{13}C NMR data are reported at 253 K due to the extremely fluxional nature of the species resulting in broad signals at room temperature on the NMR timescale, which made accurate assignment and characterization impossible.

** ^{1}H-^{15}N HMBC NMR data was initially measured at 253 K, but no cross peaks were observed. Remeasurement at 298 K allowed for observation of a single cross peak, correpsonding to the ^3J coupling of H-18 and N-22. However, although a series of different spectra optimized to different $^nJ_{N\text{-}H}$ values (1, 3, and 5 Hz), was acquired, the other N correlation (N-23) was not observed.

M.p.: 180.0-186.6 °C (decomposition).

Anal. calc'd for $C_{70}H_{73}BiN_4F_6$: C, 65.01; H, 5.69; N, 4.33; Bi, 16.16; F, 8.81; found C, 64.87; H, 5.69; N, 4.31; Bi, 16.06; F, 8.72.

Stability: air-stable, light-sensitive in solid state. Highly oxygen sensitive when in solution.

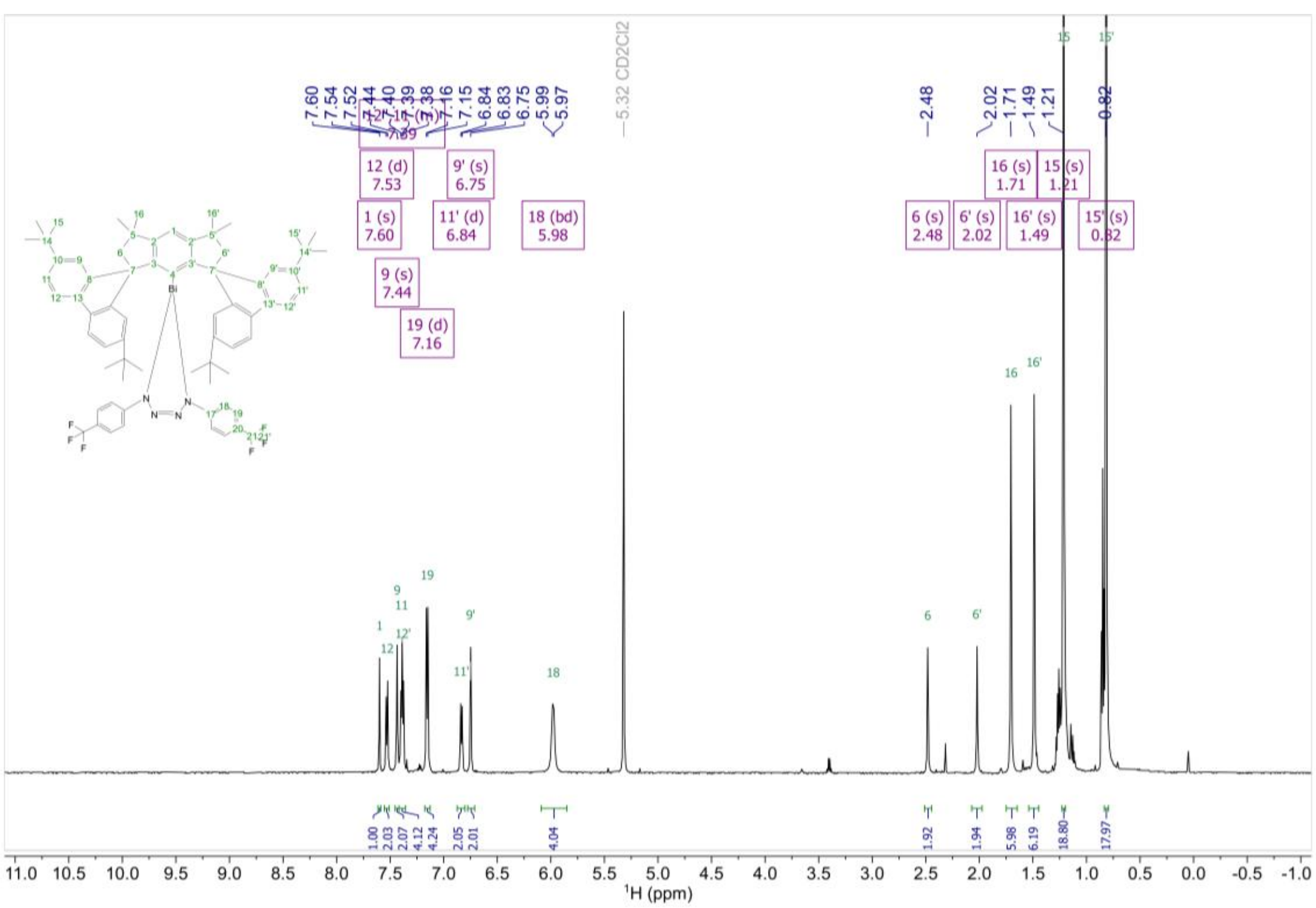

Figure S25. ^{1}H NMR spectrum of **4** (600 MHz, CD_2Cl_2, 253 K).

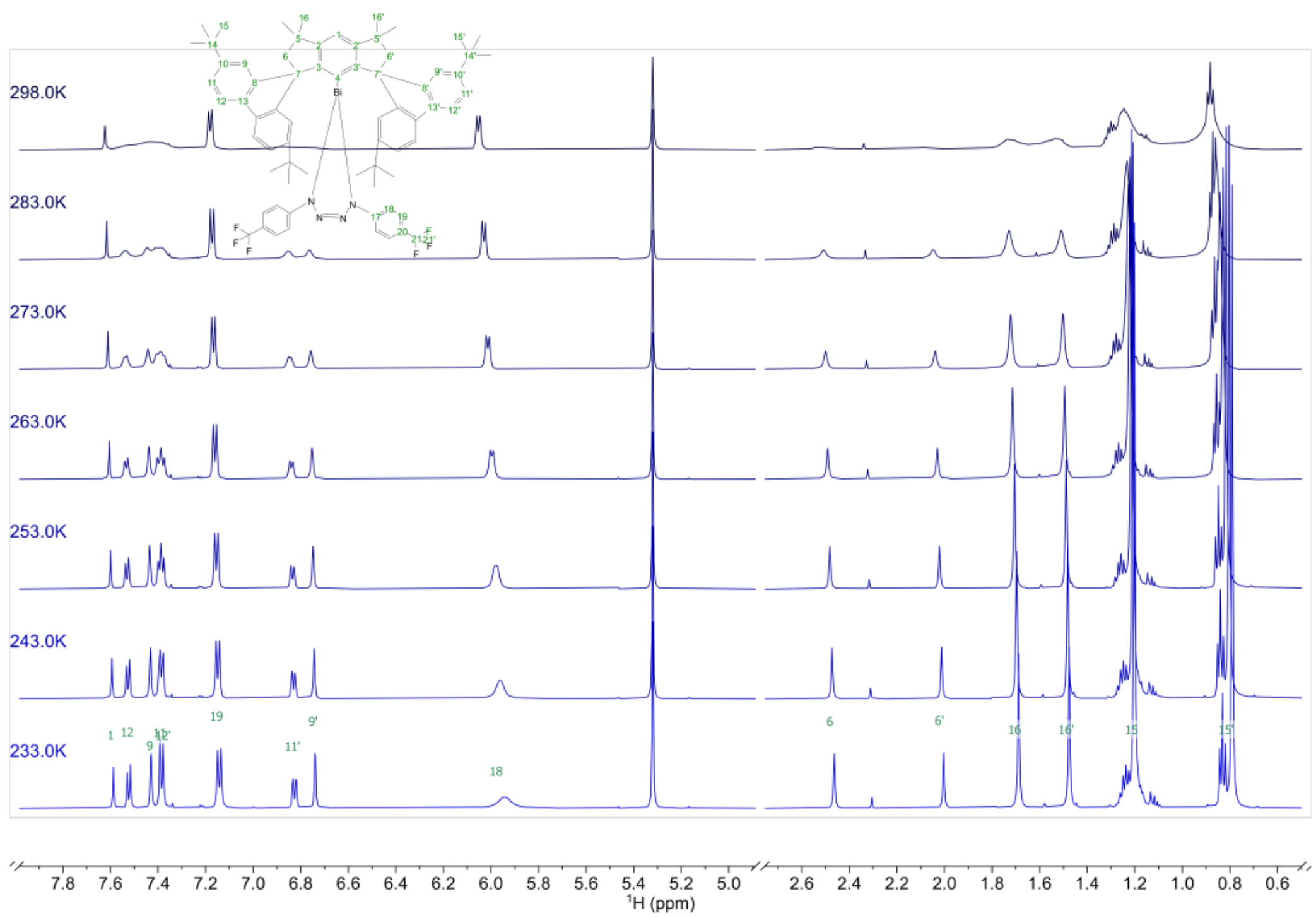

Figure S26. Stacked Variable Temperature (VT) ^{1}H NMR spectra of **4** (600 MHz, CD_2Cl_2).

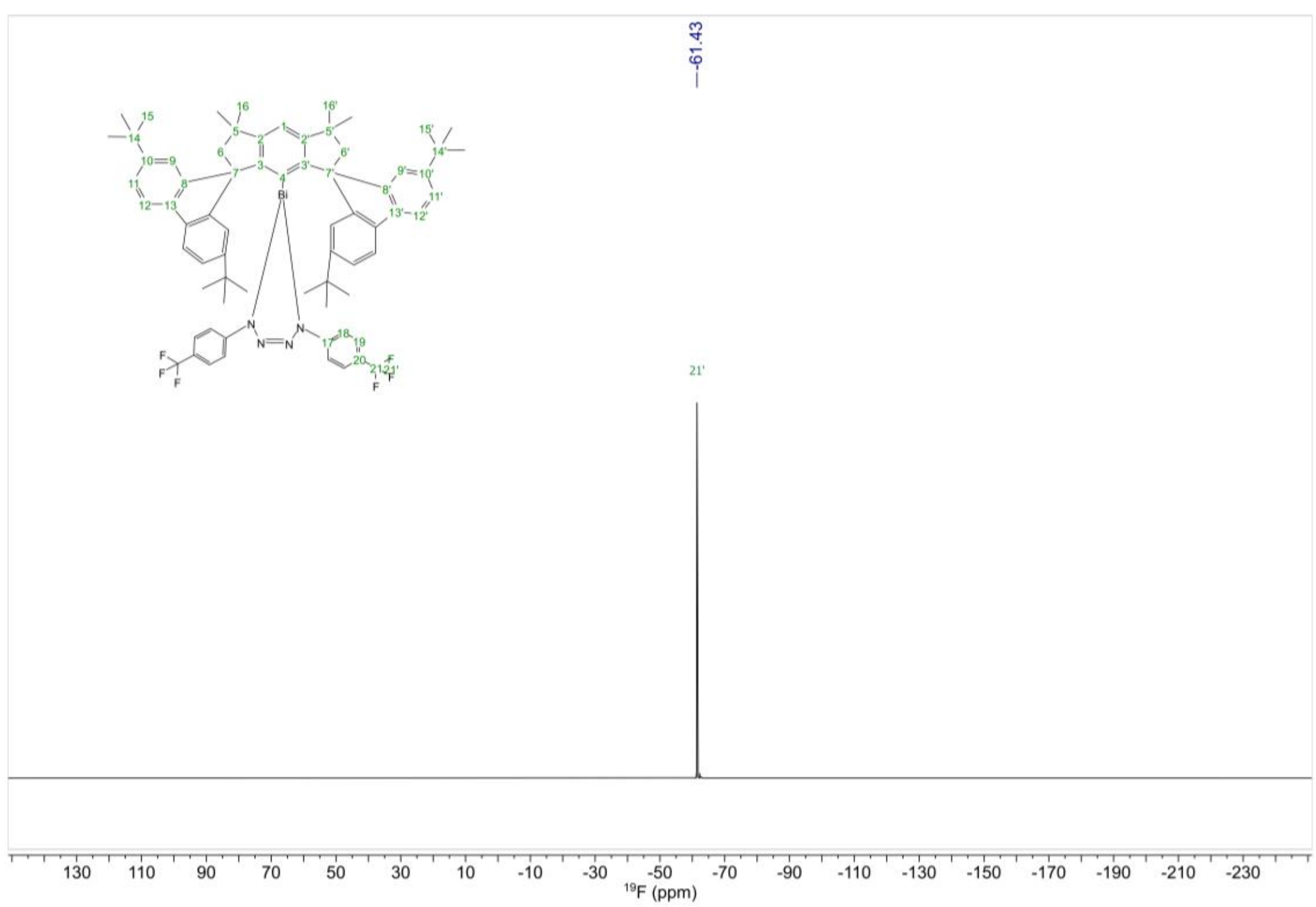

Figure S27. ^{19}F NMR spectrum of **4** (549 MHz, CD_2Cl_2, 253 K).

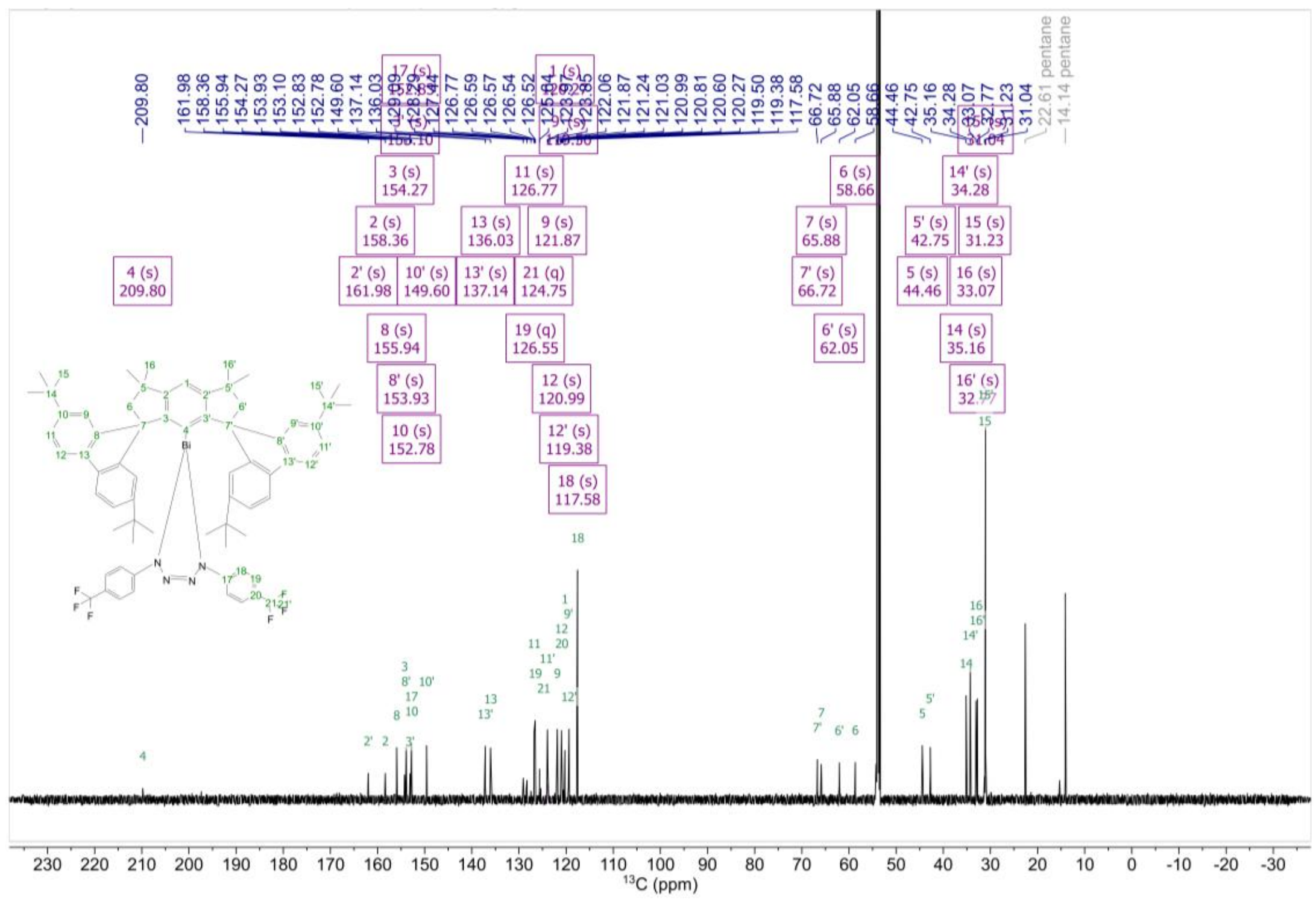

Figure S28. ^{13}C NMR spectrum of **4** (125 MHz, CD_2Cl_2, 253 K).

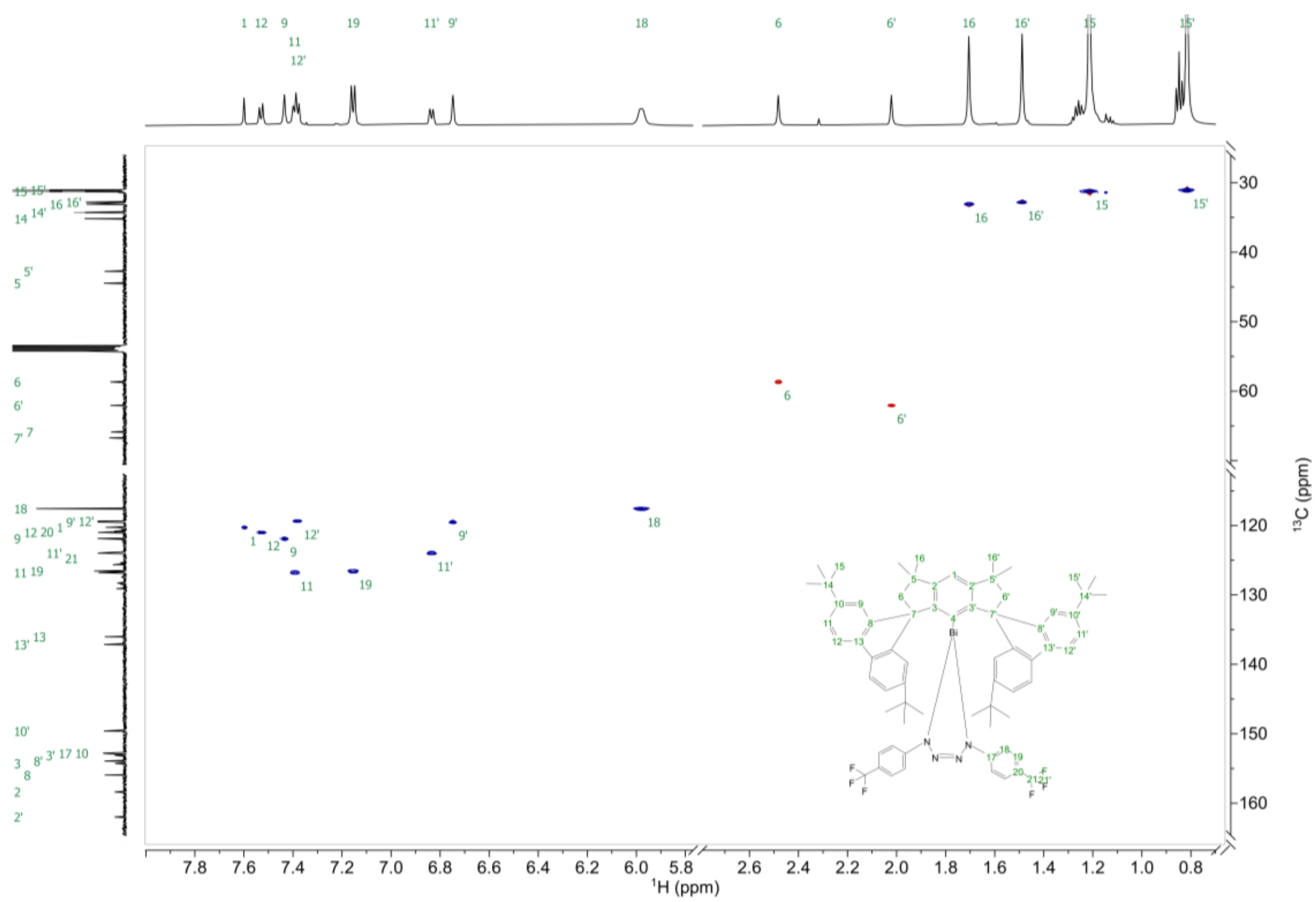

Figure S29. ^{1}H-^{13}C edited HSQC spectrum of **4** (600 MHz, CD_2Cl_2, 253 K).

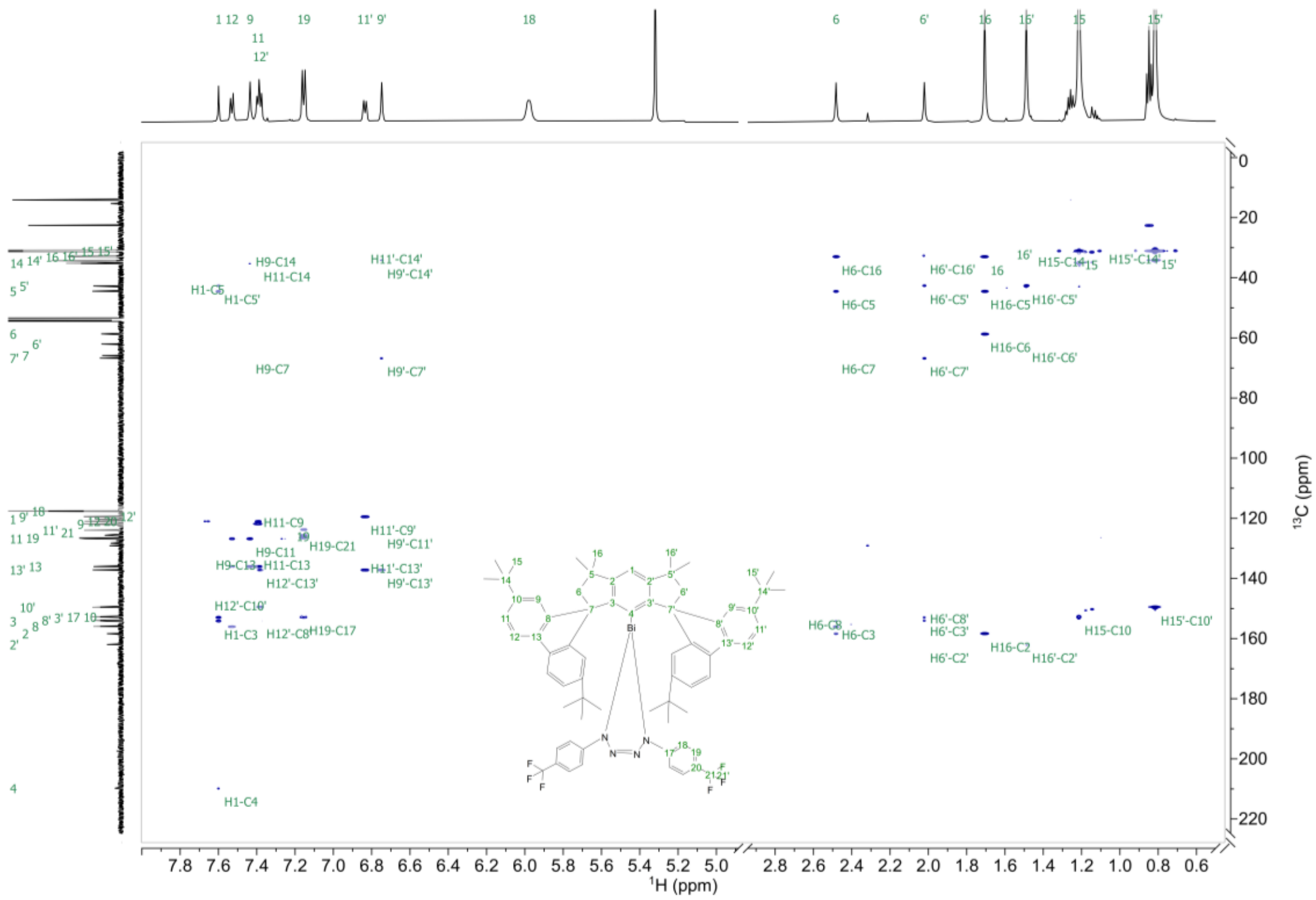

Figure S30. ^{1}H-^{13}C HMBC spectrum of **4** (600 MHz, CD_2Cl_2, 253 K).

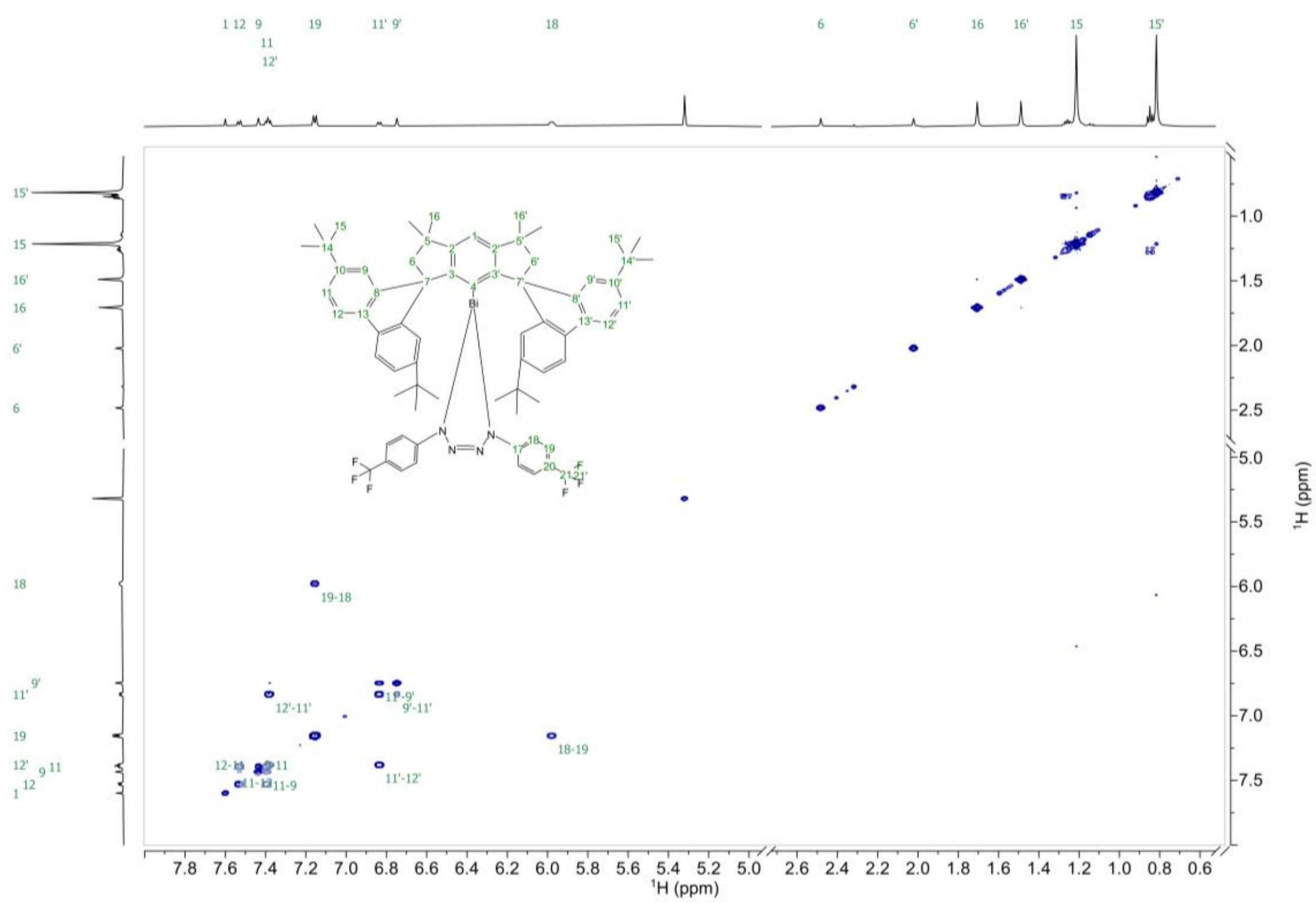

Figure S31. ^{1}H-^{1}H COSY spectrum of **4** (600 MHz, CD_2Cl_2, 253 K).

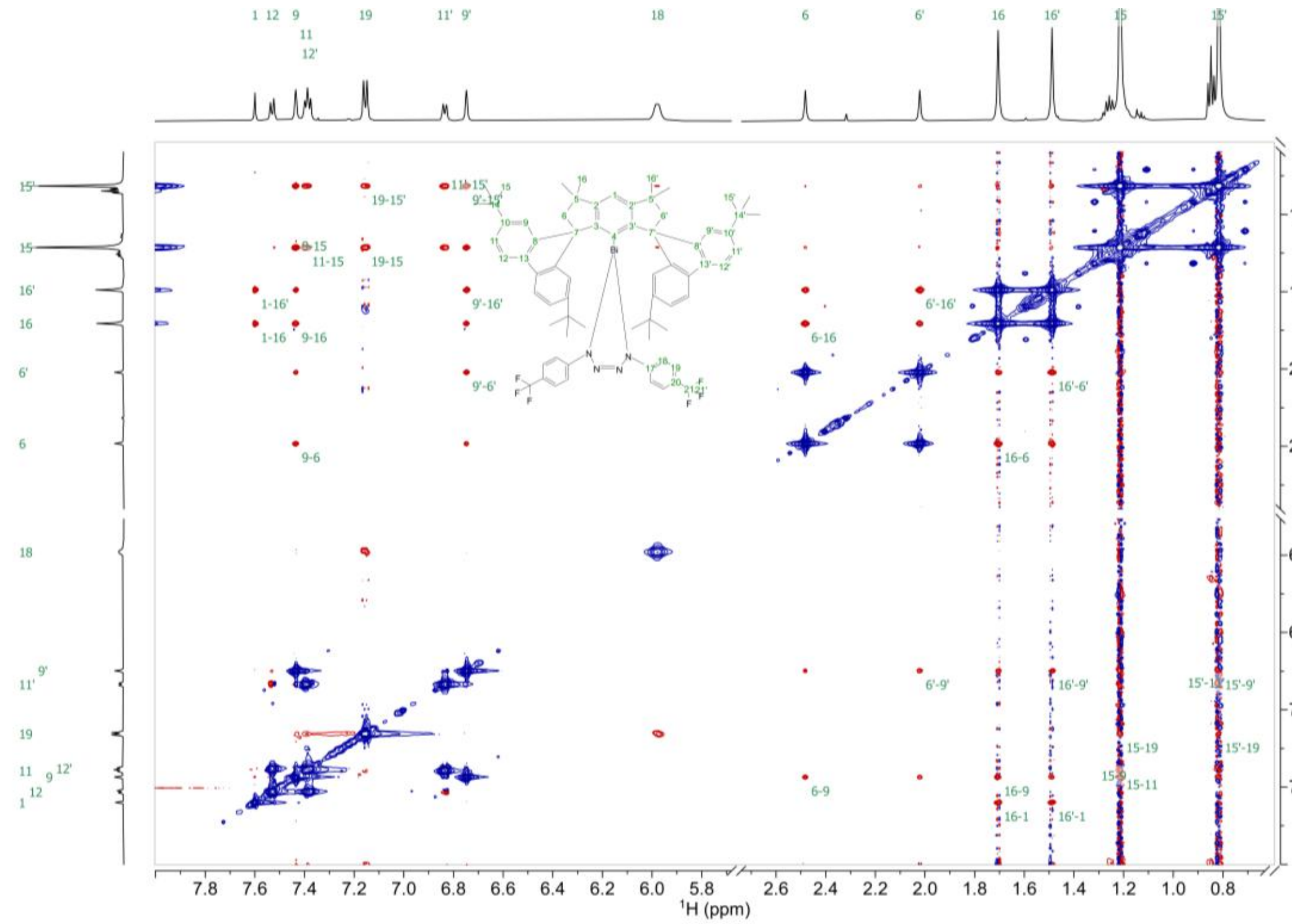

Figure S32. ^1H-^1H ROESY spectrum of **4** (600 MHz, CD_2Cl_2, 253 K).

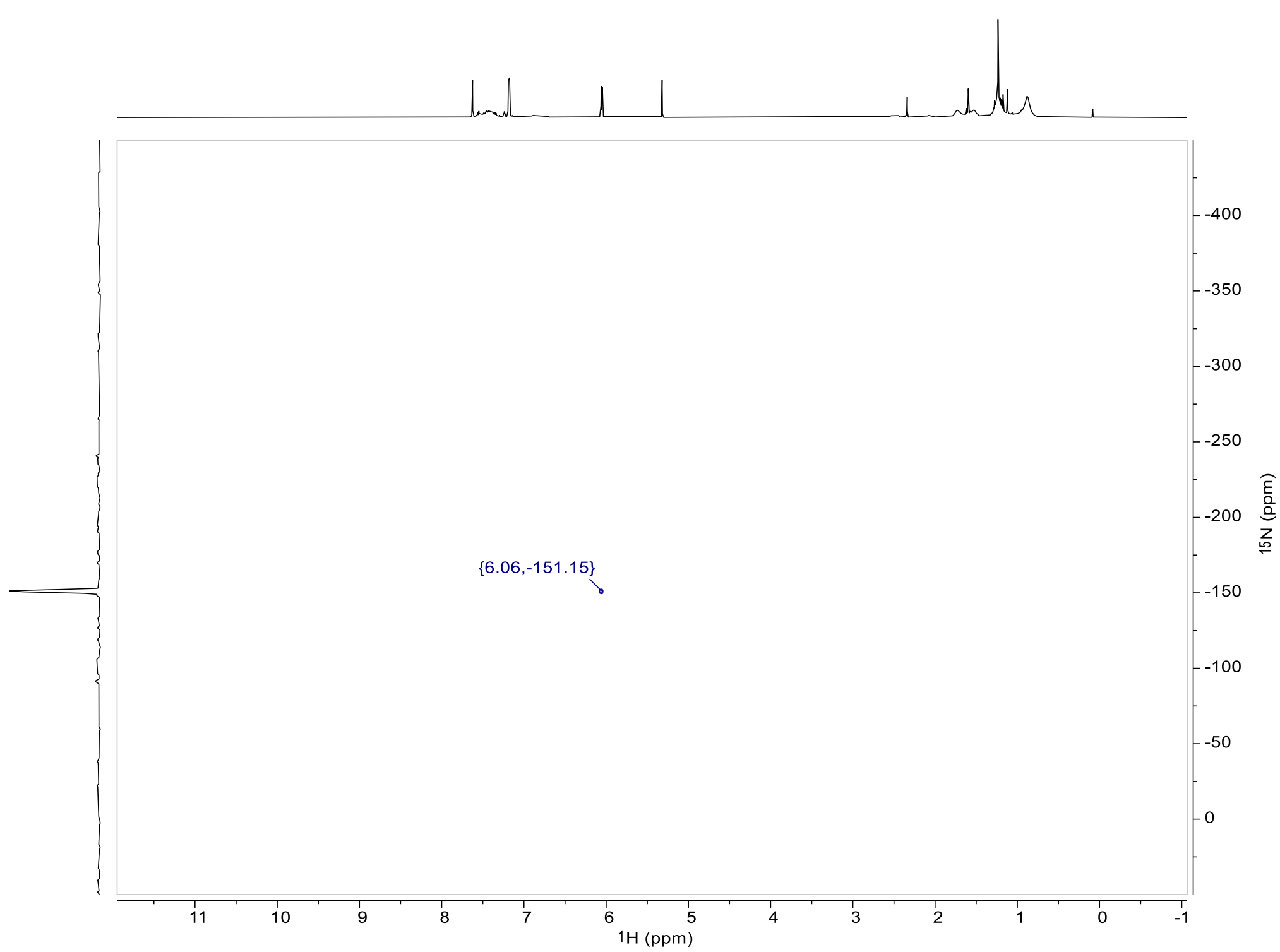

Figure S33. ^1H-^{15}N HMBC spectrum of **4** (61 MHz, CD_2Cl_2, 298 K).

3. X-ray and Electron Diffraction Crystal Structure Analyses

3.1. Single crystal structure analysis of 2 · THF solvate

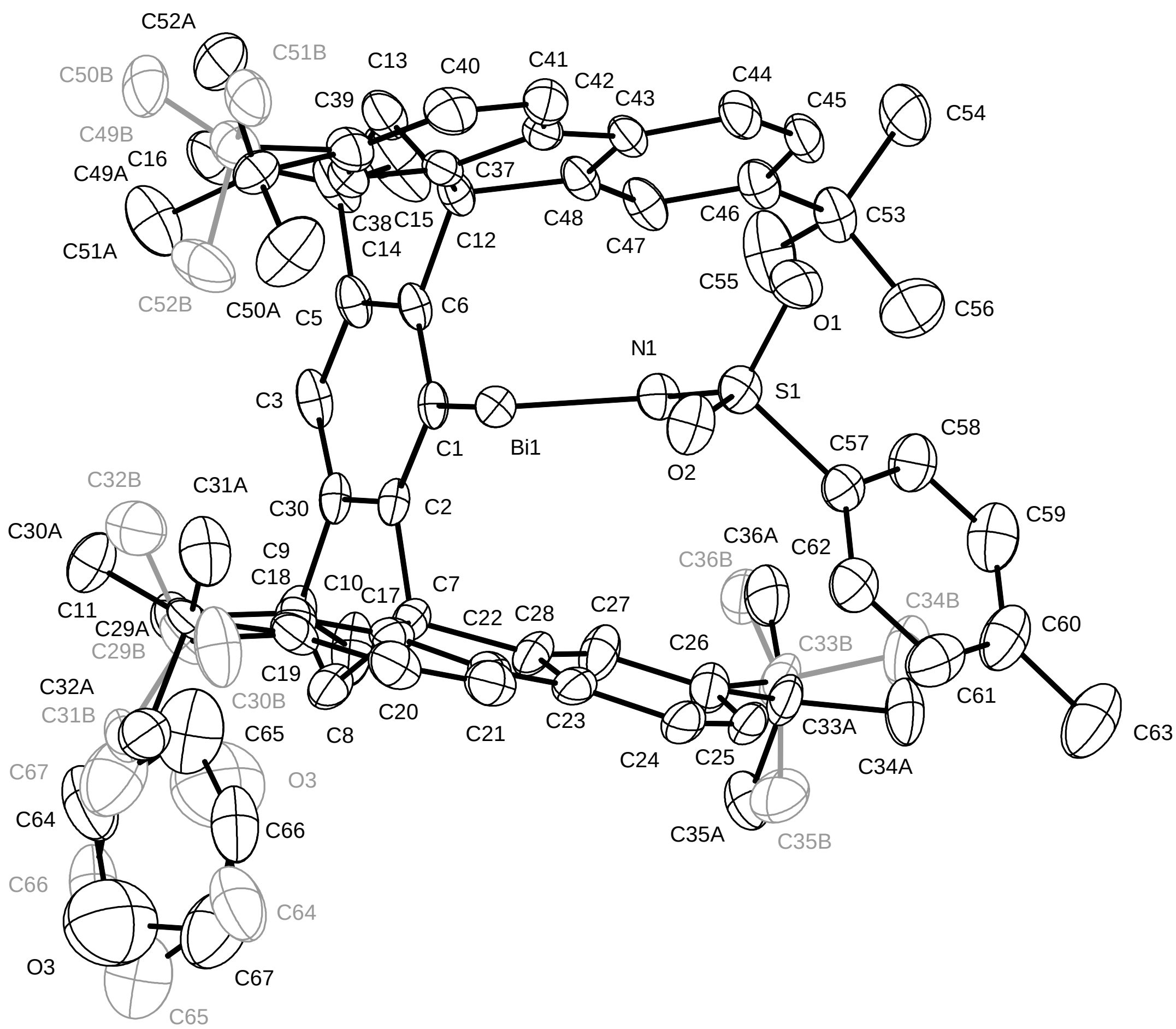

Figure S34. The molecular structure of **2 · THF solvate**. H atoms have been removed for clarity and disordered parts shown in gray.

X-ray Crystal Structure Analysis of **2 · THF solvate**:

$C_{130}H_{152}Bi_2N_2O_5S_2$, M_r = 2304.61 g mol^{-1}, yellow orange plate, crystal size 0.145 x 0.136 x 0.078 mm^3, Triclinic, space group *P*-1 [2], a = 10.7322(4) Å, b = 15.1719(6) Å, c = 18.6742(7) Å, α = 71.381(2)°, β = 76.449(2)°, γ = 80.847(2)°, *V* = 2789.58(19) Å^3, *T* = 100(2) K, *Z* = 1, D_{calc} = 1.372 g·cm^3, λ = 0.71073 Å, μ(*Mo-K*$_\alpha$) = 3.243 mm^{-1}, Gaussian absorption correction ($T_{\min}$ = 0.71388, $T_{\max}$ = 0.86264), Bruker-AXS Kappa Mach3 with APEX-II detector and IµS microfocus Mo-anode X-ray source, θ range 1.173 to 28.667°, 79390 measured reflections, 14270 independent reflections, 12188 reflections with $I > 2\sigma(I)$, R_{int} = 0.0512. The structure was solved by *SHELXT* and refined by full-matrix least-squares (*SHELXL*) against F^2 to R_1 = 0.0393 [$I > 2\sigma(I)$], wR_2 = 0.0779 [all data] with 795 parameters and 525 restraints.

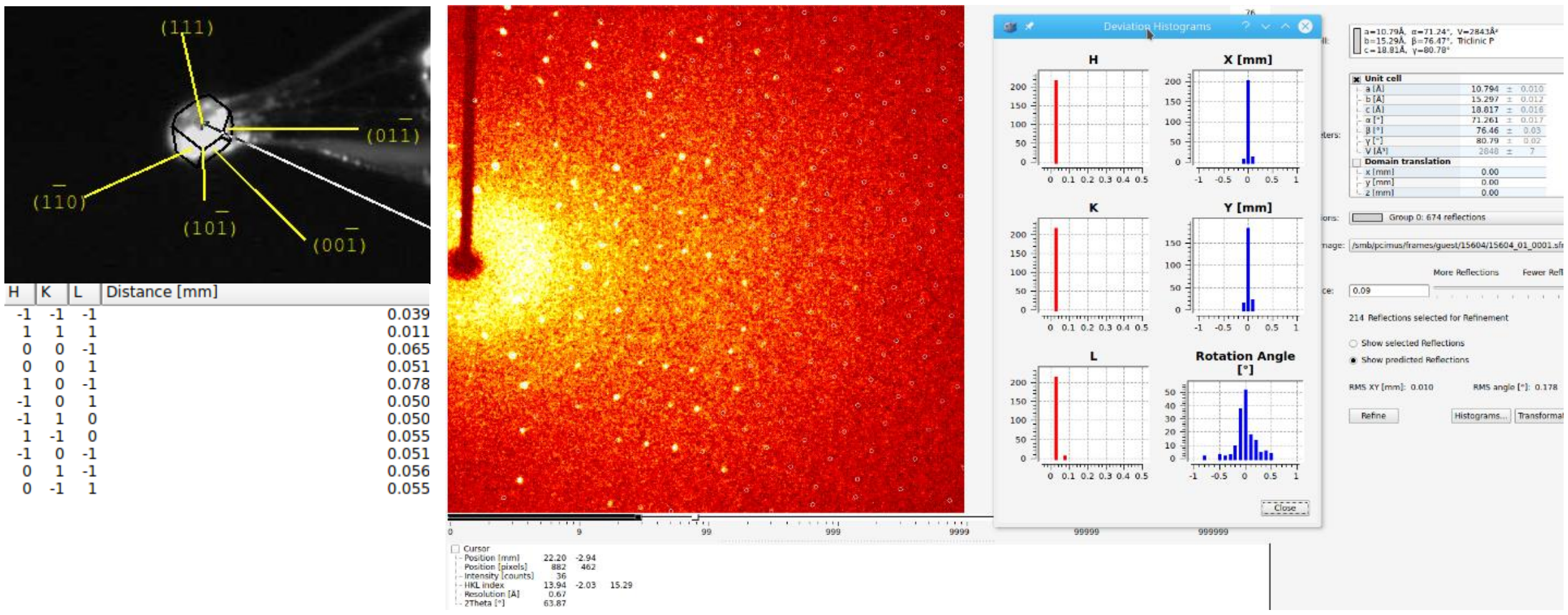

Figure S35. Crystal faces and unit cell determination/refinement of **2 · THF solvate**.

INTENSITY STATISTICS FOR DATASET

Resolution	#Data	#Theory	%Complete	Redundancy	Mean I	Mean I/s	Rmerge	Rsigma
Inf - 3.01	416	434	95.9	4.50	134.84	68.23	0.0167	0.0120
3.01 - 2.03	950	982	96.7	4.59	87.06	58.33	0.0200	0.0141
2.03 - 1.62	1312	1350	97.2	4.52	58.14	46.45	0.0252	0.0179
1.62 - 1.41	1379	1414	97.5	4.49	46.26	38.97	0.0299	0.0216
1.41 - 1.28	1380	1408	98.0	4.43	37.65	32.66	0.0368	0.0260
1.28 - 1.19	1304	1330	98.0	4.31	32.44	28.25	0.0435	0.0307
1.19 - 1.12	1414	1436	98.5	4.17	28.09	23.59	0.0516	0.0362
1.12 - 1.06	1398	1444	96.8	3.43	24.19	18.24	0.0615	0.0496
1.06 - 1.02	1174	1234	95.1	2.90	21.19	14.58	0.0732	0.0634
1.02 - 0.97	1685	1786	94.3	2.56	18.88	12.15	0.0777	0.0770
0.97 - 0.94	1184	1262	93.8	2.36	15.95	9.90	0.0924	0.0953
0.94 - 0.91	1333	1428	93.3	2.17	15.94	9.31	0.0921	0.1029
0.91 - 0.88	1509	1640	92.0	1.98	12.95	7.45	0.1049	0.1302
0.88 - 0.86	1136	1236	91.9	1.97	11.67	6.59	0.1185	0.1471
0.86 - 0.84	1211	1322	91.6	1.88	10.23	5.72	0.1357	0.1727
0.84 - 0.82	1361	1494	91.1	1.84	9.95	5.37	0.1355	0.1827
0.82 - 0.80	1499	1632	91.9	1.81	8.60	4.61	0.1577	0.2167
0.80 - 0.78	1629	1802	90.4	1.73	7.77	4.05	0.1797	0.2475
0.78 - 0.77	846	934	90.6	1.68	7.46	3.75	0.1781	0.2677
0.77 - 0.75	1921	2142	89.7	1.67	6.66	3.35	0.2006	0.3017
0.75 - 0.74	722	1040	69.4	1.20	6.21	2.93	0.2175	0.3448
0.84 - 0.74	7978	9044	88.2	1.68	7.86	4.08	0.1707	0.2472
Inf - 0.74	26763	28750	93.1	2.76	24.34	17.16	0.0447	0.0622

Complete CIF-data of the compound is available under the CCDC number **2495377**.

Three reflections (0 3 0; 5 -2 7 and 6 -1 4) showed a high I/sI and were omitted before the final refinement cycles. Three of the terminal tBu groups exhibit slight disorder across two distinct positions, which were described using the 'tert-butyl-C' fragment of the DSR tool as a plug-in in Olex2.[5-7] The corresponding parts were refined using free variables. DFIX was also employed to optimise the bond lengths between the quaternary carbon atom and the carbon atom in the aromatic ring. The ISOR instruction was used to improve some of the atomic displacement parameters.

Furthermore, the structure contains a disordered THF solute molecule, described using the 'THF' fragment of the DSR tool as a plug-in in Olex2. This molecule is located at a special position (inversion centre) and was refined using PART -1 with a fixed occupancy of 50:50.

Table S1. Bond lengths [Å] and angles [°] of **2 · THF solvate**.

Internal identification code	15604	
Empirical formula	$C_{130} H_{152} Bi_2 N_2 O_5 S_2$	
Color	yellow orange	
Formula weight	2304.61 g·mol^{-1}	
Temperature	100(2) K	
Wavelength	0.71073 Å	
Crystal system	Triclinic	
Space group	*P*-1, (no. 2)	
Unit cell dimensions	a = 10.7322(4) Å	α = 71.381(2)°.
	b = 15.1719(6) Å	β = 76.449(2)°.
	c = 18.6742(7) Å	γ = 80.847(2)°.
Volume	2789.58(19) Å^3	
Z	1	
Density (calculated)	1.372 Mg·m^{-3}	
Absorption coefficient	3.243 mm^{-1}	
F(000)	1184 e	
Crystal size	0.145 x 0.136 x 0.078 mm^3	
θ range for data collection	1.173 to 28.667°	
Index ranges	$-14 \leq h \leq 14$, $-20 \leq k \leq 20$, $-25 \leq l \leq 25$	
Reflections collected	79390	
Independent reflections	14270 [R_{int} = 0.0512]	
Reflections with I>2σ(I)	12188	
Completeness to θ = 25.242°	100 %	
Absorption correction	Gaussian	
Max. and min. transmission	0.86264 and 0.71388	
Refinement method	Full-matrix least-squares on F^2	
Data / restraints / parameters	14270 / 525 / 795	
Goodness-of-fit on F^2	1.166	
Final R indices [I>2σ(I)]	R_1 = 0.0393	wR^2 = 0.0747
R indices (all data)	R_1 = 0.0531	wR^2 = 0.0779
Extinction coefficient	n/a	
Largest diff. peak and hole	1.216 and -1.776 e·Å^{-3}	

Table S2. Bond lengths [Å] and angles [°] of **2 · THF solvate**.

Bi(1)-N(1)	2.041(3)	Bi(1)-C(1)	2.266(3)
S(1)-O(1)	1.440(3)	S(1)-O(2)	1.452(3)
S(1)-N(1)	1.594(3)	S(1)-C(57)	1.775(4)
C(1)-C(2)	1.399(5)	C(1)-C(6)	1.380(5)
C(2)-C(7)	1.514(5)	C(2)-C(30)	1.398(5)
C(3)-H(3)	0.9500	C(3)-C(5)	1.395(5)
C(3)-C(30)	1.379(5)	C(5)-C(6)	1.400(5)
C(5)-C(14)	1.512(6)	C(6)-C(12)	1.516(5)
C(7)-C(8)	1.557(5)	C(7)-C(17)	1.527(6)
C(7)-C(28)	1.535(5)	C(8)-H(8A)	0.9900
C(8)-H(8B)	0.9900	C(8)-C(9)	1.560(6)
C(9)-C(10)	1.524(6)	C(9)-C(11)	1.535(5)
C(9)-C(30)	1.516(5)	C(10)-H(10A)	0.9800
C(10)-H(10B)	0.9800	C(10)-H(10C)	0.9800
C(11)-H(11A)	0.9800	C(11)-H(11B)	0.9800
C(11)-H(11C)	0.9800	C(12)-C(13)	1.553(5)
C(12)-C(37)	1.519(5)	C(12)-C(48)	1.527(5)
C(13)-H(13A)	0.9900	C(13)-H(13B)	0.9900
C(13)-C(14)	1.558(6)	C(14)-C(15)	1.538(6)
C(14)-C(16)	1.535(6)	C(15)-H(15A)	0.9800
C(15)-H(15B)	0.9800	C(15)-H(15C)	0.9800
C(16)-H(16A)	0.9800	C(16)-H(16B)	0.9800
C(16)-H(16C)	0.9800	C(17)-C(18)	1.381(6)
C(17)-C(22)	1.404(5)	C(18)-H(18)	0.9500
C(18)-C(19)	1.406(5)	C(19)-C(20)	1.402(5)
C(19)-C(29A)	1.538(7)	C(19)-C(29B)	1.506(8)
C(20)-H(20)	0.9500	C(20)-C(21)	1.373(7)
C(21)-H(21)	0.9500	C(21)-C(22)	1.393(6)
C(22)-C(23)	1.456(6)	C(23)-C(24)	1.387(6)
C(23)-C(28)	1.392(6)	C(24)-H(24)	0.9500
C(24)-C(25)	1.378(7)	C(25)-H(25)	0.9500
C(25)-C(26)	1.393(7)	C(26)-C(27)	1.396(6)
C(26)-C(33A)	1.516(7)	C(26)-C(33B)	1.538(9)
C(27)-H(27)	0.9500	C(27)-C(28)	1.363(6)

C(37)-C(38)	1.379(5)	C(37)-C(42)	1.404(5)
C(38)-H(38)	0.9500	C(38)-C(39)	1.398(6)
C(39)-C(40)	1.398(6)	C(39)-C(49A)	1.537(6)
C(39)-C(49B)	1.527(9)	C(40)-H(40)	0.9500
C(40)-C(41)	1.385(6)	C(41)-H(41)	0.9500
C(41)-C(42)	1.384(6)	C(42)-C(43)	1.460(5)
C(43)-C(44)	1.382(5)	C(43)-C(48)	1.395(5)
C(44)-H(44)	0.9500	C(44)-C(45)	1.385(6)
C(45)-H(45)	0.9500	C(45)-C(46)	1.399(6)
C(46)-C(47)	1.395(5)	C(46)-C(53)	1.523(6)
C(47)-H(47)	0.9500	C(47)-C(48)	1.374(6)
C(53)-C(54)	1.523(6)	C(53)-C(55)	1.529(6)
C(53)-C(56)	1.523(6)	C(54)-H(54A)	0.9800
C(54)-H(54B)	0.9800	C(54)-H(54C)	0.9800
C(55)-H(55A)	0.9800	C(55)-H(55B)	0.9800
C(55)-H(55C)	0.9800	C(56)-H(56A)	0.9800
C(56)-H(56B)	0.9800	C(56)-H(56C)	0.9800
C(57)-C(58)	1.387(6)	C(57)-C(62)	1.363(6)
C(58)-H(58)	0.9500	C(58)-C(59)	1.377(6)
C(59)-H(59)	0.9500	C(59)-C(60)	1.390(7)
C(60)-C(61)	1.380(7)	C(60)-C(63)	1.513(7)
C(61)-H(61)	0.9500	C(61)-C(62)	1.394(6)
C(62)-H(62)	0.9500	C(63)-H(63A)	0.9800
C(63)-H(63B)	0.9800	C(63)-H(63C)	0.9800
C(49A)-C(50A)	1.539(8)	C(49A)-C(51A)	1.536(9)
C(49A)-C(52A)	1.542(9)	C(50A)-H(50A)	0.9800
C(50A)-H(50B)	0.9800	C(50A)-H(50C)	0.9800
C(51A)-H(51A)	0.9800	C(51A)-H(51B)	0.9800
C(51A)-H(51C)	0.9800	C(52A)-H(52A)	0.9800
C(52A)-H(52B)	0.9800	C(52A)-H(52C)	0.9800
C(49B)-C(50B)	1.51(2)	C(49B)-C(51B)	1.53(2)
C(49B)-C(52B)	1.50(2)	C(50B)-H(50D)	0.9800
C(50B)-H(50E)	0.9800	C(50B)-H(50F)	0.9800
C(51B)-H(51D)	0.9800	C(51B)-H(51E)	0.9800
C(51B)-H(51F)	0.9800	C(52B)-H(52D)	0.9800
C(52B)-H(52E)	0.9800	C(52B)-H(52F)	0.9800

C(29A)-C(30A)	1.552(7)	C(29A)-C(31A)	1.550(8)
C(29A)-C(32A)	1.550(7)	C(30A)-H(30A)	0.9800
C(30A)-H(30B)	0.9800	C(30A)-H(30C)	0.9800
C(31A)-H(31A)	0.9800	C(31A)-H(31B)	0.9800
C(31A)-H(31C)	0.9800	C(32A)-H(32A)	0.9800
C(32A)-H(32B)	0.9800	C(32A)-H(32C)	0.9800
C(29B)-C(30B)	1.484(11)	C(29B)-C(31B)	1.488(11)
C(29B)-C(32B)	1.484(11)	C(30B)-H(30D)	0.9800
C(30B)-H(30E)	0.9800	C(30B)-H(30F)	0.9800
C(31B)-H(31D)	0.9800	C(31B)-H(31E)	0.9800
C(31B)-H(31F)	0.9800	C(32B)-H(32D)	0.9800
C(32B)-H(32E)	0.9800	C(32B)-H(32F)	0.9800
C(33A)-C(34A)	1.517(10)	C(33A)-C(35A)	1.513(10)
C(33A)-C(36A)	1.512(10)	C(34A)-H(34A)	0.9800
C(34A)-H(34B)	0.9800	C(34A)-H(34C)	0.9800
C(35A)-H(35A)	0.9800	C(35A)-H(35B)	0.9800
C(35A)-H(35C)	0.9800	C(36A)-H(36A)	0.9800
C(36A)-H(36B)	0.9800	C(36A)-H(36C)	0.9800
C(33B)-C(34B)	1.579(11)	C(33B)-C(35B)	1.569(12)
C(33B)-C(36B)	1.577(12)	C(34B)-H(34D)	0.9800
C(34B)-H(34E)	0.9800	C(34B)-H(34F)	0.9800
C(35B)-H(35D)	0.9800	C(35B)-H(35E)	0.9800
C(35B)-H(35F)	0.9800	C(36B)-H(36D)	0.9800
C(36B)-H(36E)	0.9800	C(36B)-H(36F)	0.9800
O(3)-C(64)	1.478(16)	O(3)-C(67)	1.42(3)
C(64)-H(64A)	0.9900	C(64)-H(64B)	0.9900
C(64)-C(65)	1.64(3)	C(65)-H(65A)	0.9900
C(65)-H(65B)	0.9900	C(65)-C(66)	1.554(13)
C(66)-H(66A)	0.9900	C(66)-H(66B)	0.9900
C(66)-C(67)	1.500(12)	C(67)-H(67A)	0.9900
C(67)-H(67B)	0.9900		

N(1)-Bi(1)-C(1)	94.46(12)	O(1)-S(1)-O(2)	
114.97(18)	O(1)-S(1)-N(1)	110.98(17)	O(1)-S(1)-
C(57)	105.76(19)	O(2)-S(1)-N(1)	
114.36(17)	O(2)-S(1)-C(57)	107.56(19)	N(1)-S(1)-

C(57)	101.91(17)	S(1)-N(1)-Bi(1)	
113.34(17)	C(2)-C(1)-Bi(1)	120.9(3)	C(6)-C(1)-
Bi(1)	120.5(3)	C(6)-C(1)-C(2)	117.7(3)
C(1)-C(2)-C(7)	127.3(3)	C(30)-C(2)-C(1)	121.2(3)
C(30)-C(2)-C(7)	111.5(3)	C(5)-C(3)-H(3)	120.4
C(30)-C(3)-H(3)	120.4	C(30)-C(3)-C(5)	119.2(3)
C(3)-C(5)-C(6)	119.8(4)	C(3)-C(5)-C(14)	127.7(3)
C(6)-C(5)-C(14)	112.4(3)	C(1)-C(6)-C(5)	121.7(3)
C(1)-C(6)-C(12)	127.1(3)	C(5)-C(6)-C(12)	111.2(3)
C(2)-C(7)-C(8)	102.6(3)	C(2)-C(7)-C(17)	113.3(3)
C(2)-C(7)-C(28)	111.0(3)	C(17)-C(7)-C(8)	114.4(3)
C(17)-C(7)-C(28)	101.3(3)	C(28)-C(7)-C(8)	114.7(3)
C(7)-C(8)-H(8A)	110.0	C(7)-C(8)-H(8B)	110.0
C(7)-C(8)-C(9)	108.5(3)	H(8A)-C(8)-H(8B)	108.4
C(9)-C(8)-H(8A)	110.0	C(9)-C(8)-H(8B)	110.0
C(10)-C(9)-C(8)	112.4(4)	C(10)-C(9)-C(11)	107.7(3)
C(11)-C(9)-C(8)	110.3(4)	C(30)-C(9)-C(8)	102.4(3)
C(30)-C(9)-C(10)	112.4(4)	C(30)-C(9)-C(11)	111.7(3)
C(9)-C(10)-H(10A)	109.5	C(9)-C(10)-H(10B)	109.5
C(9)-C(10)-H(10C)	109.5	H(10A)-C(10)-H(10B)	109.5
H(10A)-C(10)-H(10C)	109.5	H(10B)-C(10)-H(10C)	109.5
C(9)-C(11)-H(11A)	109.5	C(9)-C(11)-H(11B)	109.5
C(9)-C(11)-H(11C)	109.5	H(11A)-C(11)-H(11B)	109.5
H(11A)-C(11)-H(11C)	109.5	H(11B)-C(11)-H(11C)	109.5
C(6)-C(12)-C(13)	102.2(3)	C(6)-C(12)-C(37)	113.8(3)
C(6)-C(12)-C(48)	113.2(3)	C(37)-C(12)-C(13)	112.7(3)
C(37)-C(12)-C(48)	101.1(3)	C(48)-C(12)-C(13)	114.4(3)
C(12)-C(13)-H(13A)	109.9	C(12)-C(13)-H(13B)	109.9
C(12)-C(13)-C(14)	108.7(3)	H(13A)-C(13)-H(13B)	108.3
C(14)-C(13)-H(13A)	109.9	C(14)-C(13)-H(13B)	109.9
C(5)-C(14)-C(13)	102.1(3)	C(5)-C(14)-C(15)	111.0(4)
C(5)-C(14)-C(16)	111.3(3)	C(15)-C(14)-C(13)	113.4(3)
C(16)-C(14)-C(13)	110.3(4)	C(16)-C(14)-C(15)	108.7(4)
C(14)-C(15)-H(15A)	109.5	C(14)-C(15)-H(15B)	109.5
C(14)-C(15)-H(15C)	109.5	H(15A)-C(15)-H(15B)	109.5
H(15A)-C(15)-H(15C)	109.5	H(15B)-C(15)-H(15C)	109.5

C(14)-C(16)-H(16A)	109.5	C(14)-C(16)-H(16B)	109.5
C(14)-C(16)-H(16C)	109.5	H(16A)-C(16)-H(16B)	109.5
H(16A)-C(16)-H(16C)	109.5	H(16B)-C(16)-H(16C)	109.5
C(18)-C(17)-C(7)	128.8(3)	C(18)-C(17)-C(22)	120.9(4)
C(22)-C(17)-C(7)	110.3(4)	C(17)-C(18)-H(18)	119.7
C(17)-C(18)-C(19)	120.7(3)	C(19)-C(18)-H(18)	119.7
C(18)-C(19)-C(29A)	122.7(4)	C(18)-C(19)-C(29B)	120.0(6)
C(20)-C(19)-C(18)	117.0(4)	C(20)-C(19)-C(29A)	120.2(4)
C(20)-C(19)-C(29B)	122.0(7)	C(19)-C(20)-H(20)	118.5
C(21)-C(20)-C(19)	123.0(4)	C(21)-C(20)-H(20)	118.5
C(20)-C(21)-H(21)	120.4	C(20)-C(21)-C(22)	119.3(4)
C(22)-C(21)-H(21)	120.4	C(17)-C(22)-C(23)	108.8(4)
C(21)-C(22)-C(17)	119.1(4)	C(21)-C(22)-C(23)	132.1(4)
C(24)-C(23)-C(22)	131.4(4)	C(24)-C(23)-C(28)	119.4(4)
C(28)-C(23)-C(22)	109.2(3)	C(23)-C(24)-H(24)	120.5
C(25)-C(24)-C(23)	119.0(4)	C(25)-C(24)-H(24)	120.5
C(24)-C(25)-H(25)	118.8	C(24)-C(25)-C(26)	122.4(4)
C(26)-C(25)-H(25)	118.8	C(25)-C(26)-C(27)	117.3(4)
C(25)-C(26)-C(33A)	118.6(6)	C(25)-C(26)-C(33B)	125.0(7)
C(27)-C(26)-C(33A)	123.9(7)	C(27)-C(26)-C(33B)	117.7(7)
C(26)-C(27)-H(27)	119.5	C(28)-C(27)-C(26)	121.1(4)
C(28)-C(27)-H(27)	119.5	C(23)-C(28)-C(7)	110.3(4)
C(27)-C(28)-C(7)	128.9(4)	C(27)-C(28)-C(23)	120.8(4)
C(2)-C(30)-C(9)	112.3(3)	C(3)-C(30)-C(2)	120.4(3)
C(3)-C(30)-C(9)	127.3(3)	C(38)-C(37)-C(12)	128.2(3)
C(38)-C(37)-C(42)	121.1(4)	C(42)-C(37)-C(12)	110.7(3)
C(37)-C(38)-H(38)	119.6	C(37)-C(38)-C(39)	120.8(4)
C(39)-C(38)-H(38)	119.6	C(38)-C(39)-C(40)	117.1(4)
C(38)-C(39)-C(49A)	125.5(4)	C(38)-C(39)-C(49B)	113.3(7)
C(40)-C(39)-C(49A)	116.9(4)	C(40)-C(39)-C(49B)	128.5(7)
C(39)-C(40)-H(40)	118.7	C(41)-C(40)-C(39)	122.7(4)
C(41)-C(40)-H(40)	118.7	C(40)-C(41)-H(41)	120.3
C(42)-C(41)-C(40)	119.4(4)	C(42)-C(41)-H(41)	120.3
C(37)-C(42)-C(43)	108.3(3)	C(41)-C(42)-C(37)	118.8(4)
C(41)-C(42)-C(43)	132.8(4)	C(44)-C(43)-C(42)	131.3(4)
C(44)-C(43)-C(48)	119.6(4)	C(48)-C(43)-C(42)	108.9(3)

C(43)-C(44)-H(44)	120.5	C(43)-C(44)-C(45)	119.0(4)
C(45)-C(44)-H(44)	120.5	C(44)-C(45)-H(45)	118.8
C(44)-C(45)-C(46)	122.4(4)	C(46)-C(45)-H(45)	118.8
C(45)-C(46)-C(53)	119.8(4)	C(47)-C(46)-C(45)	117.4(4)
C(47)-C(46)-C(53)	122.8(4)	C(46)-C(47)-H(47)	119.6
C(48)-C(47)-C(46)	120.8(4)	C(48)-C(47)-H(47)	119.6
C(43)-C(48)-C(12)	110.5(3)	C(47)-C(48)-C(12)	128.7(3)
C(47)-C(48)-C(43)	120.8(3)	C(46)-C(53)-C(55)	111.9(3)
C(46)-C(53)-C(56)	109.4(4)	C(54)-C(53)-C(46)	110.2(4)
C(54)-C(53)-C(55)	107.7(4)	C(54)-C(53)-C(56)	108.6(4)
C(56)-C(53)-C(55)	108.8(4)	C(53)-C(54)-H(54A)	109.5
C(53)-C(54)-H(54B)	109.5	C(53)-C(54)-H(54C)	109.5
H(54A)-C(54)-H(54B)	109.5	H(54A)-C(54)-H(54C)	109.5
H(54B)-C(54)-H(54C)	109.5	C(53)-C(55)-H(55A)	109.5
C(53)-C(55)-H(55B)	109.5	C(53)-C(55)-H(55C)	109.5
H(55A)-C(55)-H(55B)	109.5	H(55A)-C(55)-H(55C)	109.5
H(55B)-C(55)-H(55C)	109.5	C(53)-C(56)-H(56A)	109.5
C(53)-C(56)-H(56B)	109.5	C(53)-C(56)-H(56C)	109.5
H(56A)-C(56)-H(56B)	109.5	H(56A)-C(56)-H(56C)	109.5
H(56B)-C(56)-H(56C)	109.5	C(58)-C(57)-S(1)	117.5(3)
C(62)-C(57)-S(1)	121.5(3)	C(62)-C(57)-C(58)	121.0(4)
C(57)-C(58)-H(58)	120.3	C(59)-C(58)-C(57)	119.3(4)
C(59)-C(58)-H(58)	120.3	C(58)-C(59)-H(59)	119.5
C(58)-C(59)-C(60)	120.9(5)	C(60)-C(59)-H(59)	119.5
C(59)-C(60)-C(63)	120.4(5)	C(61)-C(60)-C(59)	118.4(4)
C(61)-C(60)-C(63)	121.1(5)	C(60)-C(61)-H(61)	119.4
C(60)-C(61)-C(62)	121.2(5)	C(62)-C(61)-H(61)	119.4
C(57)-C(62)-C(61)	119.1(4)	C(57)-C(62)-H(62)	120.5
C(61)-C(62)-H(62)	120.5	C(60)-C(63)-H(63A)	109.5
C(60)-C(63)-H(63B)	109.5	C(60)-C(63)-H(63C)	109.5
H(63A)-C(63)-H(63B)	109.5	H(63A)-C(63)-H(63C)	109.5
H(63B)-C(63)-H(63C)	109.5	C(39)-C(49A)-C(50A)	113.2(6)
C(39)-C(49A)-C(52A)	108.9(7)	C(50A)-C(49A)-C(52A)	
107.9(7)	C(51A)-C(49A)-C(39)	111.5(6)	C(51A)-
C(49A)-C(50A)	108.1(7)	C(51A)-C(49A)-C(52A)	
107.0(8)	C(49A)-C(50A)-H(50A)	109.5	C(49A)-

C(50A)-H(50B)	109.5	C(49A)-C(50A)-H(50C)	109.5
H(50A)-C(50A)-H(50B)	109.5	H(50A)-C(50A)-H(50C)	109.5
H(50B)-C(50A)-H(50C)	109.5	C(49A)-C(51A)-H(51A)	109.5
C(49A)-C(51A)-H(51B)	109.5	C(49A)-C(51A)-H(51C)	109.5
H(51A)-C(51A)-H(51B)	109.5	H(51A)-C(51A)-H(51C)	109.5
H(51B)-C(51A)-H(51C)	109.5	C(49A)-C(52A)-H(52A)	109.5
C(49A)-C(52A)-H(52B)	109.5	C(49A)-C(52A)-H(52C)	109.5
H(52A)-C(52A)-H(52B)	109.5	H(52A)-C(52A)-H(52C)	109.5
H(52B)-C(52A)-H(52C)	109.5	C(50B)-C(49B)-C(39)	117.2(11)
C(50B)-C(49B)-C(51B)	110.9(12)	C(51B)-C(49B)-C(39)	107.4(11)
C(52B)-C(49B)-C(39)	102.9(11)	C(52B)-C(49B)-C(50B)	
107.8(14)	C(52B)-C(49B)-C(51B)	110.4(15)	C(49B)-
C(50B)-H(50D)	109.5	C(49B)-C(50B)-H(50E)	109.5
C(49B)-C(50B)-H(50F)	109.5	H(50D)-C(50B)-H(50E)	109.5
H(50D)-C(50B)-H(50F)	109.5	H(50E)-C(50B)-H(50F)	109.5
C(49B)-C(51B)-H(51D)	109.5	C(49B)-C(51B)-H(51E)	109.5
C(49B)-C(51B)-H(51F)	109.5	H(51D)-C(51B)-H(51E)	109.5
H(51D)-C(51B)-H(51F)	109.5	H(51E)-C(51B)-H(51F)	109.5
C(49B)-C(52B)-H(52D)	109.5	C(49B)-C(52B)-H(52E)	109.5
C(49B)-C(52B)-H(52F)	109.5	H(52D)-C(52B)-H(52E)	109.5
H(52D)-C(52B)-H(52F)	109.5	H(52E)-C(52B)-H(52F)	109.5
C(19)-C(29A)-C(30A)	114.7(5)	C(19)-C(29A)-C(31A)	115.2(5)
C(19)-C(29A)-C(32A)	109.7(5)	C(31A)-C(29A)-C(30A)	
104.9(6)	C(31A)-C(29A)-C(32A)	106.7(6)	C(32A)-
C(29A)-C(30A)	104.9(5)	C(29A)-C(30A)-H(30A)	109.5
C(29A)-C(30A)-H(30B)	109.5	C(29A)-C(30A)-H(30C)	109.5
H(30A)-C(30A)-H(30B)	109.5	H(30A)-C(30A)-H(30C)	109.5
H(30B)-C(30A)-H(30C)	109.5	C(29A)-C(31A)-H(31A)	109.5
C(29A)-C(31A)-H(31B)	109.5	C(29A)-C(31A)-H(31C)	109.5
H(31A)-C(31A)-H(31B)	109.5	H(31A)-C(31A)-H(31C)	109.5
H(31B)-C(31A)-H(31C)	109.5	C(29A)-C(32A)-H(32A)	109.5
C(29A)-C(32A)-H(32B)	109.5	C(29A)-C(32A)-H(32C)	109.5
H(32A)-C(32A)-H(32B)	109.5	H(32A)-C(32A)-H(32C)	109.5
H(32B)-C(32A)-H(32C)	109.5	C(30B)-C(29B)-C(19)	104.2(10)
C(30B)-C(29B)-C(31B)	114.4(12)	C(31B)-C(29B)-C(19)	107.1(9)
C(32B)-C(29B)-C(19)	103.2(11)	C(32B)-C(29B)-C(30B)	

110.4(14)	C(32B)-C(29B)-C(31B)	116.0(12)	C(29B)-
C(30B)-H(30D)	109.5	C(29B)-C(30B)-H(30E)	109.5
C(29B)-C(30B)-H(30F)	109.5	H(30D)-C(30B)-H(30E)	109.5
H(30D)-C(30B)-H(30F)	109.5	H(30E)-C(30B)-H(30F)	109.5
C(29B)-C(31B)-H(31D)	109.5	C(29B)-C(31B)-H(31E)	109.5
C(29B)-C(31B)-H(31F)	109.5	H(31D)-C(31B)-H(31E)	109.5
H(31D)-C(31B)-H(31F)	109.5	H(31E)-C(31B)-H(31F)	109.5
C(29B)-C(32B)-H(32D)	109.5	C(29B)-C(32B)-H(32E)	109.5
C(29B)-C(32B)-H(32F)	109.5	H(32D)-C(32B)-H(32E)	109.5
H(32D)-C(32B)-H(32F)	109.5	H(32E)-C(32B)-H(32F)	109.5
C(26)-C(33A)-C(34A)	110.9(8)	C(35A)-C(33A)-C(26)	108.1(10)
C(35A)-C(33A)-C(34A)	110.6(9)	C(36A)-C(33A)-C(26)	105.0(11)
C(36A)-C(33A)-C(34A)	111.5(10)	C(36A)-C(33A)-C(35A)	
110.6(11)	C(33A)-C(34A)-H(34A)	109.5	C(33A)-
C(34A)-H(34B)	109.5	C(33A)-C(34A)-H(34C)	109.5
H(34A)-C(34A)-H(34B)	109.5	H(34A)-C(34A)-H(34C)	109.5
H(34B)-C(34A)-H(34C)	109.5	C(33A)-C(35A)-H(35A)	109.5
C(33A)-C(35A)-H(35B)	109.5	C(33A)-C(35A)-H(35C)	109.5
H(35A)-C(35A)-H(35B)	109.5	H(35A)-C(35A)-H(35C)	109.5
H(35B)-C(35A)-H(35C)	109.5	C(33A)-C(36A)-H(36A)	109.5
C(33A)-C(36A)-H(36B)	109.5	C(33A)-C(36A)-H(36C)	109.5
H(36A)-C(36A)-H(36B)	109.5	H(36A)-C(36A)-H(36C)	109.5
H(36B)-C(36A)-H(36C)	109.5	C(26)-C(33B)-C(34B)	116.3(12)
C(26)-C(33B)-C(35B)	108.6(13)	C(26)-C(33B)-C(36B)	119.2(13)
C(35B)-C(33B)-C(34B)	104.5(11)	C(35B)-C(33B)-C(36B)	
103.9(12)	C(36B)-C(33B)-C(34B)	102.8(12)	C(33B)-
C(34B)-H(34D)	109.5	C(33B)-C(34B)-H(34E)	109.5
C(33B)-C(34B)-H(34F)	109.5	H(34D)-C(34B)-H(34E)	109.5
H(34D)-C(34B)-H(34F)	109.5	H(34E)-C(34B)-H(34F)	109.5
C(33B)-C(35B)-H(35D)	109.5	C(33B)-C(35B)-H(35E)	109.5
C(33B)-C(35B)-H(35F)	109.5	H(35D)-C(35B)-H(35E)	109.5
H(35D)-C(35B)-H(35F)	109.5	H(35E)-C(35B)-H(35F)	109.5
C(33B)-C(36B)-H(36D)	109.5	C(33B)-C(36B)-H(36E)	109.5
C(33B)-C(36B)-H(36F)	109.5	H(36D)-C(36B)-H(36E)	109.5
H(36D)-C(36B)-H(36F)	109.5	H(36E)-C(36B)-H(36F)	109.5
C(67)-O(3)-C(64)	106.0(13)	O(3)-C(64)-H(64A)	109.8

O(3)-C(64)-H(64B)	109.8	O(3)-C(64)-C(65)	109.3(15)
H(64A)-C(64)-H(64B)	108.3	C(65)-C(64)-H(64A)	109.8
C(65)-C(64)-H(64B)	109.8	C(64)-C(65)-H(65A)	112.3
C(64)-C(65)-H(65B)	112.3	H(65A)-C(65)-H(65B)	109.9
C(66)-C(65)-C(64)	97.4(13)	C(66)-C(65)-H(65A)	112.3
C(66)-C(65)-H(65B)	112.3	C(65)-C(66)-H(66A)	110.8
C(65)-C(66)-H(66B)	110.8	H(66A)-C(66)-H(66B)	108.9
C(67)-C(66)-C(65)	104.9(14)	C(67)-C(66)-H(66A)	110.8
C(67)-C(66)-H(66B)	110.8	O(3)-C(67)-C(66)	109.0(13)
O(3)-C(67)-H(67A)	109.9	O(3)-C(67)-H(67B)	109.9
C(66)-C(67)-H(67A)	109.9	C(66)-C(67)-H(67B)	109.9
H(67A)-C(67)-H(67B)	108.3		

3.2. Crystal structure analysis of 3

Various attempts were made to crystallise **3** using conventional methods such as slow solvent evaporation. However, these resulted in microcrystalline powders that were too small to be investigated using conventional single-crystal XRD techniques. For this reason, electron diffraction was employed to analyse the fine powder and to determine its structure.
Electron diffraction measurements were collected using a Rigaku XtaLAB Synergy-ED equipped with a Rigaku HyPix-ED detector optimised for operation in 3DED experimental setup.[8] The solid sample was ground up between microscope slides before being loaded onto a lacey carbon 200 mesh Cu grid. Cryo-transfer using the Gatan ELSA (Model 698) specimen holder was then applied to prevent desolvation of the crystals. This technique involves flash-cooling the samples in liquid nitrogen before introducing them to the XtaLAB Synergy-ED's high vacuum, leaving them unscathed. The sample was cooled to 175 K and diffraction patterns were collected using a wavelength of 0.0251 Å. Each individual measurement lasted between two and four minutes. A total of six datasets were collected. To achieve a higher level of completeness and redundancy, scans 5 and 6 were merged using CrysAlisPro program. The structure solution and refinement were performed using the resulting merged dataset.

No.	Number of frames	Scan range [°]	Scan width [°]	Exposure time [s]	Dose time [min]	Total dose [e^-/Å^2]
1	256	-63 to +65	0.5	0.5	02:08	1.63
2	248	-62 to +62	0.5	0.5	02:04	1.58
3	248	-62 to +62	0.5	1.0	04:08	3.15
4	256	-63 to +65	0.5	1.0	04:16	3.25
5	244	-61 to +61	0.5	0.5	02:02	1.55
6	252	-63 to +63	0.5	0.5	02:04	1.60

No.	Resolution [Å]	Data	Completeness [%]	Redundancy	F^2	$F^2/\sigma(F^2)$	R_{int}	R_{pim}	R_{rim}	CC1/2
1	inf- 0.83	33903	64.5	2.1	1180.87	2.14	0.207	0.188	0.291	0.984
2	inf- 0.83	64892	77.6	3.4	811.67	2.13	0.281	0.176	0.355	0.986
3	inf- 0.83	63182	89.0	2.9	907.37	1.54	0.358	0.263	0.533	0.413
4	inf- 0.83	67108	99.0	2.7	617.60	1.85	0.241	0.177	0.318	0.984
5	inf- 0.83	63632	88.6	2.9	1412.23	2.88	0.236	0.169	0.345	0.986
6	inf- 0.83	66319	97.4	2.7	1304.64	2.67	0.222	0.162	0.297	0.968

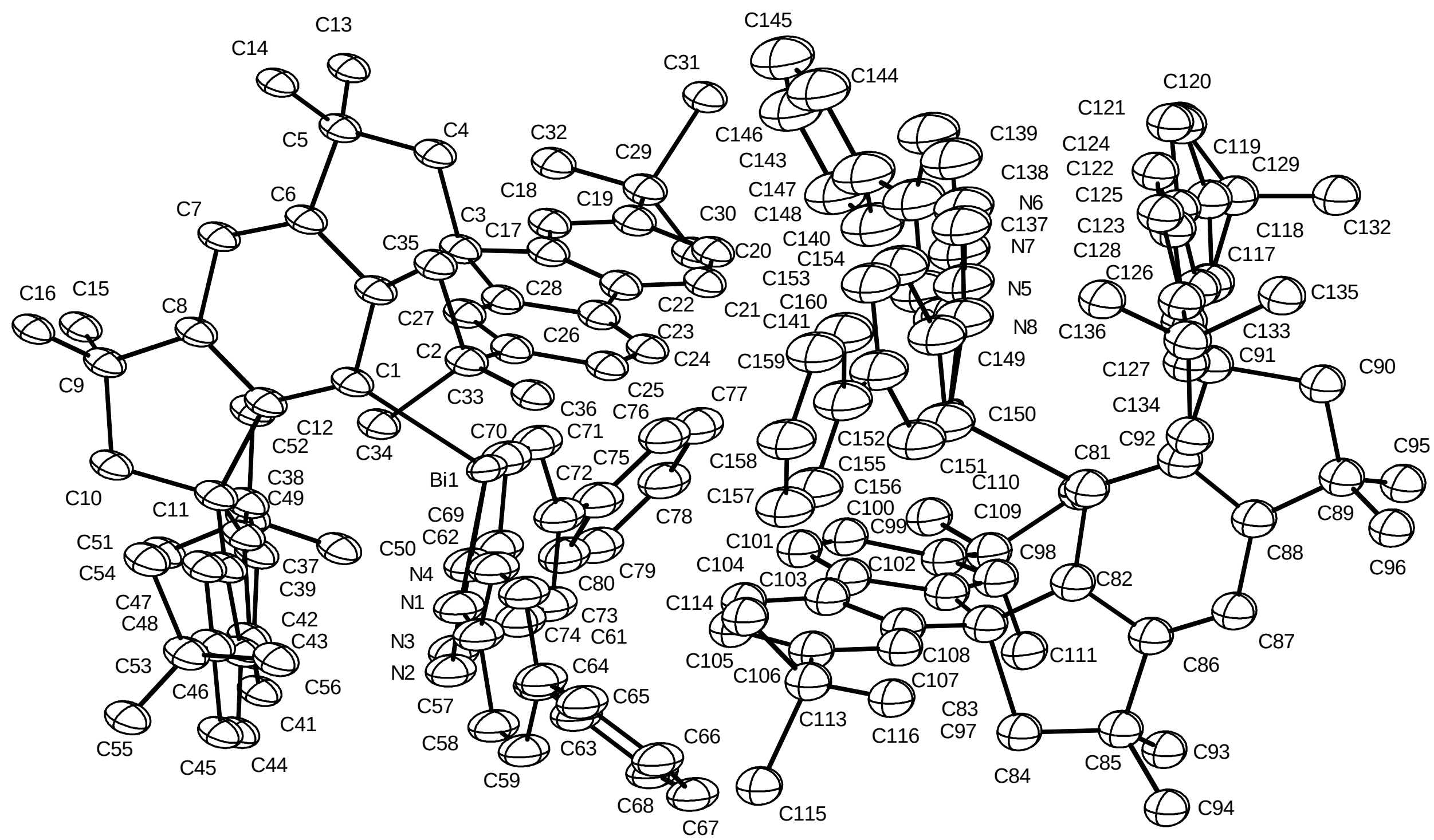

Figure S36. The molecular structure of **3**. H atoms have been removed for clarity.

3DED Crystal Structure Analysis of **3**:
C_{80} H_{83} Bi N_4, M_r = 1309.55 g mol^{-1}, white plate, approx. 0.01 x 0.005 x 0.0001 mm³, monoclinic, space group $P2_1/n$ [14], a = 12.5840(9) Å, b = 39.948(9) Å, c = 26.594(3) Å, β = 93.182(8)°, V = 13348(3) Å^3, T = 175.00(10) K, Z = 8, D_{calc} = 1.303 g·cm^3, λ = 0.0251 Å, Rigaku XtaLAB Synergy-ED equipped with a Rigaku HyPix-ED optimised for Micro-ED, LaB_6 electron gun, 200 keV (λ = 0.0251 Å) and three-stage electron-optical condenser system, A merged dataset of two single-axis rotations, θ range 0.033 to 0.866°, 128185 measured reflections, 24435 independent reflections, 12089 reflections with $I > 2\sigma(I)$, R_{int} = 0.3197. The structure was solved by *SHELXS* and refined by full-matrix least-squares (*SHELXL,* using SFAC according to UCLA)[9] against F^2 to R_1 = 0.2906 [$I > 2\sigma(I)$], wR_2 = 0.6312 [all data] with 1467 parameters and 2680 restraints. All non-hydrogen atoms were refined in anisotropic approximation, with hydrogen atoms 'riding' in idealized positions. The model is based on the kinematical approximation and no dynamical refinement was applied.

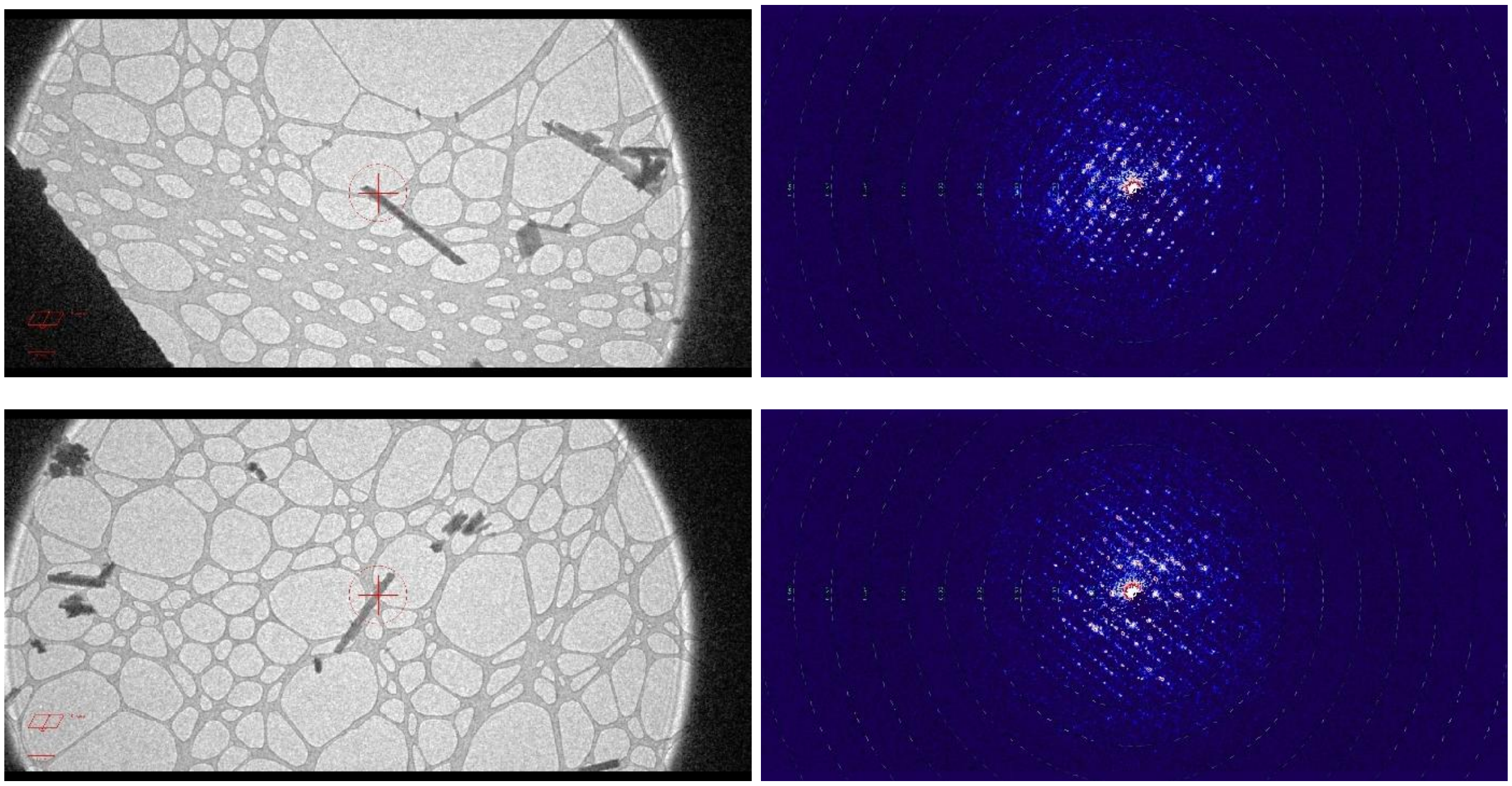

Figure S37. TEM images of crystal No. 5 and 6 (left) and exemplary, cropped diffraction pattern patterns (right) of **3**.

Statistics vs resolution (taking redundancy into account) - point group symmetry: P2/m (b-unique)[Lattice extinct removed]

resolu- tion(A)	# kept	# theory	# unique	% complete	average redundancy	mean F2	mean F2/sig(F2)	Rint	Rpim	Rurim	CC 1/2
39.95- 1.72	14546	2831	2831	100.0	5.1	7029.98	8.56	0.153	0.072	0.169	0.967
1.72- 1.36	14871	2831	2831	100.0	5.3	1553.79	5.62	0.275	0.131	0.328	0.945
1.36- 1.19	14961	2831	2831	100.0	5.3	929.47	4.18	0.387	0.186	0.491	0.828
1.19- 1.08	14991	2831	2831	100.0	5.3	737.82	3.45	0.456	0.219	0.613	0.729
1.08- 1.00	15022	2831	2831	100.0	5.3	465.71	2.51	0.564	0.273	0.873	0.581
1.00- 0.94	14967	2831	2831	100.0	5.3	313.16	1.91	0.664	0.322	1.182	0.361
0.94- 0.90	14914	2831	2831	100.0	5.3	217.16	1.41	0.764	0.374	1.578	0.149
0.90- 0.86	14509	2831	2831	100.0	5.1	178.07	1.19	0.793	0.393	1.917	0.135
0.86- 0.82	13912	2831	2831	100.0	4.9	132.05	0.88	0.829	0.422	2.573	0.076
0.82- 0.79	10572	2838	2394	84.4	4.4	116.24	0.71	0.836	0.453	2.841	0.027
39.95- 0.79	143265	28317	27873	98.4	5.1	1192.91	3.12	0.308	0.149	0.399	0.969
39.95- 0.80	142883	27753	27716	99.9	5.2	1195.80	3.13	0.307	0.148	0.397	0.969

Complete CIF-data of the compound is available under the CCDC number **2495378**.

The higher Rint values may be due to slight dynamic effects. To refine the structure, the SHEL (999 0.83) card was used to exclude the incomplete shells at higher angles. The ADPs of all the atoms were treated using SIMU and RIGU.[10.11] Nevertheless, some atoms exhibited disproportionately large deformation parameters. Distances between C63-C60, C72-C75, C140-C143 and C152-C155 were restrained using DFIX instructions. An AFIX instruction was applied to all six-membered rings of the biphenyl ring systems to improve convergence. The short distances between H atoms may be due to the use of neutron distances for the H positions. Furthermore, other areas may also be disordered, but this could not be properly resolved. The size of the crystals was estimated based on electron microscope images.

Table S3. Bond lengths [Å] and angles [°] of **3**.

Internal identification code	m_10380_10381	
Empirical formula	C_{80} H_{83} Bi N_4	
Color	white	
Formula weight	1309.55 g·mol^{-1}	
Temperature	175.00 K	
Wavelength	0.0251 Å	
Crystal system	Monoclinic	
Space group	$P2_1/n$, (no. 14)	
Unit cell dimensions	a = 12.5840(9) Å	α = 90°.
	b = 39.948(9) Å	β = 93.182(8)°.
	c = 26.594(3) Å	γ = 90°.
Volume	13348(3) Å^3	
Z	8	
Density (calculated)	1.303 Mg·m^{-3}	
Absorption coefficient	0.000 mm^{-1}	
F(000)	2131 e	
Crystal size	0.01 x 0.005 x 0.0001 mm^3	
θ range for data collection	0.033 to 0.866°.	
Index ranges	-15 ≤ h ≤ 15, -48 ≤ k ≤ 48, -32 ≤ l ≤ 32	
Reflections collected	128185	
Independent reflections	24435 [R_{int} = 0.3197]	
Reflections with I>2σ(I)	12089	
Completeness to θ = 0.863°	100.0 %	
Absorption correction	None	
Refinement method	Full-matrix least-squares on F^2	
Data / restraints / parameters	24435 / 2680 / 1467	
Goodness-of-fit on F^2	1.066	
Final R indices [I>2σ(I)]	R_1 = 0.2906	wR^2 = 0.5905
R indices (all data)	R_1 = 0.3747	wR^2 = 0.6312
Extinction coefficient	n/a	
Largest diff. peak and hole	0.292 and -0.509 e·Å^{-3}	

Table S4. Bond lengths [Å] and angles [°] of **3**.

Bi(1)-N(1)	2.25(2)	Bi(1)-N(4)	2.271(18)
Bi(1)-C(1)	2.349(19)	N(1)-N(2)	1.33(2)
N(1)-C(57)	1.37(2)	N(2)-N(3)	1.30(3)
N(3)-N(4)	1.32(3)	N(4)-C(69)	1.37(2)
C(1)-C(2)	1.40(2)	C(1)-C(12)	1.40(2)
C(2)-C(3)	1.50(2)	C(2)-C(6)	1.47(3)
C(3)-C(4)	1.52(2)	C(3)-C(17)	1.57(3)
C(3)-C(28)	1.58(3)	C(4)-H(4A)	1.1020
C(4)-H(4B)	1.1020	C(4)-C(5)	1.62(2)
C(5)-C(6)	1.49(2)	C(5)-C(13)	1.51(3)
C(5)-C(14)	1.50(3)	C(6)-C(7)	1.37(2)
C(7)-H(7)	1.1030	C(7)-C(8)	1.47(2)
C(8)-C(9)	1.52(2)	C(8)-C(12)	1.41(3)
C(9)-C(10)	1.48(2)	C(9)-C(15)	1.56(3)
C(9)-C(16)	1.56(3)	C(10)-H(10A)	1.1020
C(10)-H(10B)	1.1020	C(10)-C(11)	1.60(3)
C(11)-C(12)	1.61(2)	C(11)-C(37)	1.47(3)
C(11)-C(48)	1.46(3)	C(13)-H(13A)	1.0970
C(13)-H(13B)	1.0970	C(13)-H(13C)	1.0970
C(14)-H(14A)	1.0970	C(14)-H(14B)	1.0970
C(14)-H(14C)	1.0970	C(15)-H(15A)	1.0970
C(15)-H(15B)	1.0970	C(15)-H(15C)	1.0970
C(16)-H(16A)	1.0970	C(16)-H(16B)	1.0970
C(16)-H(16C)	1.0970	C(17)-C(18)	1.37(3)
C(17)-C(22)	1.36(3)	C(18)-H(18)	1.1030
C(18)-C(19)	1.41(3)	C(19)-C(20)	1.39(3)
C(19)-C(29)	1.50(3)	C(20)-H(20)	1.1030
C(20)-C(21)	1.39(3)	C(21)-H(21)	1.1030
C(21)-C(22)	1.39(3)	C(22)-C(23)	1.49(3)
C(23)-C(24)	1.37(3)	C(23)-C(28)	1.43(3)
C(24)-H(24)	1.1030	C(24)-C(25)	1.42(3)
C(25)-H(25)	1.1030	C(25)-C(26)	1.41(3)
C(26)-C(27)	1.48(3)	C(26)-C(33)	1.51(3)
C(27)-H(27)	1.1030	C(27)-C(28)	1.30(3)

C(29)-C(30)	1.49(3)	C(29)-C(31)	1.62(2)
C(29)-C(32)	1.57(3)	C(30)-H(30A)	1.0970
C(30)-H(30B)	1.0970	C(30)-H(30C)	1.0970
C(31)-H(31A)	1.0970	C(31)-H(31B)	1.0970
C(31)-H(31C)	1.0970	C(32)-H(32A)	1.0970
C(32)-H(32B)	1.0970	C(32)-H(32C)	1.0970
C(33)-C(34)	1.59(2)	C(33)-C(35)	1.59(2)
C(33)-C(36)	1.49(3)	C(34)-H(34A)	1.0970
C(34)-H(34B)	1.0970	C(34)-H(34C)	1.0970
C(35)-H(35A)	1.0970	C(35)-H(35B)	1.0970
C(35)-H(35C)	1.0970	C(36)-H(36A)	1.0970
C(36)-H(36B)	1.0970	C(36)-H(36C)	1.0970
C(37)-C(38)	1.37(3)	C(37)-C(42)	1.54(3)
C(38)-H(38)	1.1030	C(38)-C(39)	1.38(3)
C(39)-C(40)	1.53(3)	C(39)-C(49)	1.35(3)
C(40)-H(40)	1.1030	C(40)-C(41)	1.29(3)
C(41)-H(41)	1.1030	C(41)-C(42)	1.40(3)
C(42)-C(43)	1.43(3)	C(43)-C(44)	1.43(3)
C(43)-C(48)	1.42(3)	C(44)-H(44)	1.1030
C(44)-C(45)	1.35(3)	C(45)-H(45)	1.1030
C(45)-C(46)	1.45(3)	C(46)-C(47)	1.38(3)
C(46)-C(53)	1.53(3)	C(47)-H(47)	1.1030
C(47)-C(48)	1.36(3)	C(49)-C(50)	1.69(3)
C(49)-C(51)	1.54(2)	C(49)-C(52)	1.59(2)
C(50)-H(50A)	1.0970	C(50)-H(50B)	1.0970
C(50)-H(50C)	1.0970	C(51)-H(51A)	1.0970
C(51)-H(51B)	1.0970	C(51)-H(51C)	1.0970
C(52)-H(52A)	1.0970	C(52)-H(52B)	1.0970
C(52)-H(52C)	1.0970	C(53)-C(54)	1.56(3)
C(53)-C(55)	1.40(3)	C(53)-C(56)	1.60(3)
C(54)-H(54A)	1.0970	C(54)-H(54B)	1.0970
C(54)-H(54C)	1.0970	C(55)-H(55A)	1.0970
C(55)-H(55B)	1.0970	C(55)-H(55C)	1.0970
C(56)-H(56A)	1.0970	C(56)-H(56B)	1.0970
C(56)-H(56C)	1.0970	C(58)-H(58)	1.1030
C(58)-C(57)	1.3900	C(58)-C(59)	1.3900

C(57)-C(62)	1.3900	C(62)-H(62)	1.1030
C(62)-C(61)	1.3900	C(61)-H(61)	1.1030
C(61)-C(60)	1.3900	C(60)-C(59)	1.3900
C(60)-C(63)	1.514(8)	C(59)-H(59)	1.1030
C(63)-C(68)	1.3900	C(63)-C(64)	1.3900
C(68)-H(68)	1.1030	C(68)-C(67)	1.3900
C(67)-H(67)	1.1030	C(67)-C(66)	1.3900
C(66)-H(66)	1.1030	C(66)-C(65)	1.3900
C(65)-H(65)	1.1030	C(65)-C(64)	1.3900
C(64)-H(64)	1.1030	C(73)-H(73)	1.1030
C(73)-C(72)	1.3900	C(73)-C(74)	1.3900
C(72)-C(71)	1.3900	C(72)-C(75)	1.510(9)
C(71)-H(71)	1.1030	C(71)-C(70)	1.3900
C(70)-H(70)	1.1030	C(70)-C(69)	1.3900
C(69)-C(74)	1.3900	C(74)-H(74)	1.1030
C(75)-C(80)	1.3900	C(75)-C(76)	1.3900
C(80)-H(80)	1.1030	C(80)-C(79)	1.3900
C(79)-H(79)	1.1030	C(79)-C(78)	1.3900
C(78)-H(78)	1.1030	C(78)-C(77)	1.3900
C(77)-H(77)	1.1030	C(77)-C(76)	1.3900
C(76)-H(76)	1.1030	Bi(2)-N(5)	2.16(3)
Bi(2)-N(8)	2.22(2)	Bi(2)-C(81)	2.29(2)
N(5)-N(6)	1.44(3)	N(5)-C(137)	1.46(3)
N(6)-N(7)	1.28(4)	N(7)-N(8)	1.48(3)
N(8)-C(149)	1.38(3)	C(81)-C(82)	1.46(3)
C(81)-C(92)	1.44(3)	C(82)-C(83)	1.52(3)
C(82)-C(86)	1.42(3)	C(83)-C(84)	1.66(3)
C(83)-C(97)	1.52(3)	C(83)-C(108)	1.53(3)
C(84)-H(84A)	1.1020	C(84)-H(84B)	1.1020
C(84)-C(85)	1.47(3)	C(85)-C(86)	1.51(3)
C(85)-C(93)	1.53(3)	C(85)-C(94)	1.68(3)
C(86)-C(87)	1.30(3)	C(87)-H(87)	1.1030
C(87)-C(88)	1.47(3)	C(88)-C(89)	1.42(3)
C(88)-C(92)	1.42(3)	C(89)-C(90)	1.56(3)
C(89)-C(95)	1.46(3)	C(89)-C(96)	1.57(3)
C(90)-H(90A)	1.1020	C(90)-H(90B)	1.1020

C(90)-C(91)	1.69(3)	C(91)-C(92)	1.50(3)
C(91)-C(117)	1.44(3)	C(91)-C(128)	1.60(3)
C(93)-H(93A)	1.0970	C(93)-H(93B)	1.0970
C(93)-H(93C)	1.0970	C(94)-H(94A)	1.0970
C(94)-H(94B)	1.0970	C(94)-H(94C)	1.0970
C(95)-H(95A)	1.0970	C(95)-H(95B)	1.0970
C(95)-H(95C)	1.0970	C(96)-H(96A)	1.0970
C(96)-H(96B)	1.0970	C(96)-H(96C)	1.0970
C(97)-C(98)	1.39(3)	C(97)-C(102)	1.47(3)
C(98)-H(98)	1.1030	C(98)-C(99)	1.34(3)
C(99)-C(100)	1.47(3)	C(99)-C(109)	1.49(3)
C(100)-H(100)	1.1030	C(100)-C(101)	1.40(3)
C(101)-H(101)	1.1030	C(101)-C(102)	1.39(3)
C(102)-C(103)	1.40(3)	C(103)-C(104)	1.40(3)
C(103)-C(108)	1.42(3)	C(104)-H(104)	1.1030
C(104)-C(105)	1.46(3)	C(105)-H(105)	1.1030
C(105)-C(106)	1.41(3)	C(106)-C(107)	1.45(3)
C(106)-C(113)	1.63(3)	C(107)-H(107)	1.1030
C(107)-C(108)	1.32(3)	C(109)-C(110)	1.64(3)
C(109)-C(111)	1.58(3)	C(109)-C(112)	1.62(3)
C(110)-H(11A)	1.0970	C(110)-H(11B)	1.0970
C(110)-H(11C)	1.0970	C(111)-H(11D)	1.0970
C(111)-H(11E)	1.0970	C(111)-H(11F)	1.0970
C(112)-H(11G)	1.0970	C(112)-H(11H)	1.0970
C(112)-H(11I)	1.0970	C(113)-C(114)	1.50(3)
C(113)-C(115)	1.68(3)	C(113)-C(116)	1.38(3)
C(114)-H(11J)	1.0970	C(114)-H(11K)	1.0970
C(114)-H(11L)	1.0970	C(115)-H(11M)	1.0970
C(115)-H(11N)	1.0970	C(115)-H(11O)	1.0970
C(116)-H(11P)	1.0970	C(116)-H(11Q)	1.0970
C(116)-H(11R)	1.0970	C(117)-C(118)	1.34(3)
C(117)-C(122)	1.40(3)	C(118)-H(118)	1.1030
C(118)-C(119)	1.46(3)	C(119)-C(120)	1.39(3)
C(119)-C(129)	1.53(3)	C(120)-H(120)	1.1030
C(120)-C(121)	1.42(3)	C(121)-H(121)	1.1030
C(121)-C(122)	1.46(3)	C(122)-C(123)	1.39(3)

C(123)-C(124)	1.44(3)	C(123)-C(128)	1.40(3)
C(124)-H(124)	1.1030	C(124)-C(125)	1.41(3)
C(125)-H(125)	1.1030	C(125)-C(126)	1.34(3)
C(126)-C(127)	1.42(3)	C(126)-C(133)	1.55(3)
C(127)-H(127)	1.1030	C(127)-C(128)	1.44(3)
C(129)-C(130)	1.51(3)	C(129)-C(131)	1.52(3)
C(129)-C(132)	1.51(3)	C(130)-H(13D)	1.0970
C(130)-H(13E)	1.0970	C(130)-H(13F)	1.0970
C(131)-H(13G)	1.0970	C(131)-H(13H)	1.0970
C(131)-H(13I)	1.0970	C(132)-H(13J)	1.0970
C(132)-H(13K)	1.0970	C(132)-H(13L)	1.0970
C(133)-C(134)	1.45(3)	C(133)-C(135)	1.61(3)
C(133)-C(136)	1.66(3)	C(134)-H(13M)	1.0970
C(134)-H(13N)	1.0970	C(134)-H(13O)	1.0970
C(135)-H(13P)	1.0970	C(135)-H(13Q)	1.0970
C(135)-H(13R)	1.0970	C(136)-H(13S)	1.0970
C(136)-H(13T)	1.0970	C(136)-H(13U)	1.0970
C(137)-C(142)	1.3900	C(137)-C(138)	1.3900
C(142)-H(142)	1.1030	C(142)-C(141)	1.3900
C(141)-H(141)	1.1030	C(141)-C(140)	1.3900
C(140)-C(139)	1.3900	C(140)-C(143)	1.509(9)
C(139)-H(139)	1.1030	C(139)-C(138)	1.3900
C(138)-H(138)	1.1030	C(143)-C(148)	1.3900
C(143)-C(144)	1.3900	C(148)-H(148)	1.1030
C(148)-C(147)	1.3900	C(147)-H(147)	1.1030
C(147)-C(146)	1.3900	C(146)-H(146)	1.1030
C(146)-C(145)	1.3900	C(145)-H(145)	1.1030
C(145)-C(144)	1.3900	C(144)-H(144)	1.1030
C(154)-H(154)	1.1030	C(154)-C(149)	1.3900
C(154)-C(153)	1.3900	C(149)-C(150)	1.3900
C(150)-H(150)	1.1030	C(150)-C(151)	1.3900
C(151)-H(151)	1.1030	C(151)-C(152)	1.3900
C(152)-C(153)	1.3900	C(152)-C(155)	1.485(9)
C(153)-H(153)	1.1030	C(155)-C(160)	1.3900
C(155)-C(156)	1.3900	C(160)-H(160)	1.1030
C(160)-C(159)	1.3900	C(159)-H(159)	1.1030

C(159)-C(158)	1.3900	C(158)-H(158)	1.1030
C(158)-C(157)	1.3900	C(157)-H(157)	1.1030
C(157)-C(156)	1.3900	C(156)-H(156)	1.1030
N(1)-Bi(1)-N(4)	72.6(7)	N(1)-Bi(1)-C(1)	110.1(7)
N(4)-Bi(1)-C(1)	106.8(6)	N(2)-N(1)-Bi(1)	111.6(16)
N(2)-N(1)-C(57)	120.3(17)	C(57)-N(1)-Bi(1)	125.2(11)
N(3)-N(2)-N(1)	123.1(19)	N(2)-N(3)-N(4)	119.6(18)
N(3)-N(4)-Bi(1)	113.2(15)	N(3)-N(4)-C(69)	120.6(17)
C(69)-N(4)-Bi(1)	123.0(12)	C(2)-C(1)-Bi(1)	109.2(13)
C(12)-C(1)-Bi(1)	127.8(13)	C(12)-C(1)-C(2)	121.6(17)
C(1)-C(2)-C(3)	132.5(17)	C(1)-C(2)-C(6)	118.2(16)
C(6)-C(2)-C(3)	109.3(14)	C(2)-C(3)-C(4)	104.5(15)
C(2)-C(3)-C(17)	115.7(17)	C(2)-C(3)-C(28)	116.1(17)
C(4)-C(3)-C(17)	112.3(17)	C(4)-C(3)-C(28)	108.9(17)
C(17)-C(3)-C(28)	99.4(16)	C(3)-C(4)-H(4A)	109.7
C(3)-C(4)-H(4B)	109.7	C(3)-C(4)-C(5)	109.7(14)
H(4A)-C(4)-H(4B)	108.2	C(5)-C(4)-H(4A)	109.7
C(5)-C(4)-H(4B)	109.7	C(6)-C(5)-C(4)	99.7(14)
C(6)-C(5)-C(13)	111.3(18)	C(6)-C(5)-C(14)	111.8(18)
C(13)-C(5)-C(4)	114.4(17)	C(14)-C(5)-C(4)	108.7(17)
C(14)-C(5)-C(13)	110.4(16)	C(2)-C(6)-C(5)	113.7(15)
C(7)-C(6)-C(2)	122.8(16)	C(7)-C(6)-C(5)	123.4(17)
C(6)-C(7)-H(7)	122.3	C(6)-C(7)-C(8)	115.4(17)
C(8)-C(7)-H(7)	122.3	C(7)-C(8)-C(9)	120.8(16)
C(12)-C(8)-C(7)	122.8(17)	C(12)-C(8)-C(9)	115.9(15)
C(8)-C(9)-C(15)	112.8(18)	C(8)-C(9)-C(16)	108.8(16)
C(10)-C(9)-C(8)	102.1(15)	C(10)-C(9)-C(15)	112.6(17)
C(10)-C(9)-C(16)	110.5(19)	C(16)-C(9)-C(15)	109.7(16)
C(9)-C(10)-H(10A)	109.5	C(9)-C(10)-H(10B)	109.5
C(9)-C(10)-C(11)	110.9(15)	H(10A)-C(10)-H(10B)	108.0
C(11)-C(10)-H(10A)	109.5	C(11)-C(10)-H(10B)	109.5
C(10)-C(11)-C(12)	101.3(14)	C(37)-C(11)-C(10)	111.7(16)
C(37)-C(11)-C(12)	113.9(18)	C(48)-C(11)-C(10)	108.1(18)
C(48)-C(11)-C(12)	114.9(16)	C(48)-C(11)-C(37)	106.9(16)
C(1)-C(12)-C(8)	118.3(16)	C(1)-C(12)-C(11)	134.2(17)

C(8)-C(12)-C(11)	106.4(15)	C(5)-C(13)-H(13A)	109.5
C(5)-C(13)-H(13B)	109.5	C(5)-C(13)-H(13C)	109.5
H(13A)-C(13)-H(13B)	109.5	H(13A)-C(13)-H(13C)	109.5
H(13B)-C(13)-H(13C)	109.5	C(5)-C(14)-H(14A)	109.5
C(5)-C(14)-H(14B)	109.5	C(5)-C(14)-H(14C)	109.5
H(14A)-C(14)-H(14B)	109.5	H(14A)-C(14)-H(14C)	109.5
H(14B)-C(14)-H(14C)	109.5	C(9)-C(15)-H(15A)	109.5
C(9)-C(15)-H(15B)	109.5	C(9)-C(15)-H(15C)	109.5
H(15A)-C(15)-H(15B)	109.5	H(15A)-C(15)-H(15C)	109.5
H(15B)-C(15)-H(15C)	109.5	C(9)-C(16)-H(16A)	109.5
C(9)-C(16)-H(16B)	109.5	C(9)-C(16)-H(16C)	109.5
H(16A)-C(16)-H(16B)	109.5	H(16A)-C(16)-H(16C)	109.5
H(16B)-C(16)-H(16C)	109.5	C(18)-C(17)-C(3)	124.9(18)
C(22)-C(17)-C(3)	110(2)	C(22)-C(17)-C(18)	125(2)
C(17)-C(18)-H(18)	121.4	C(17)-C(18)-C(19)	117.3(19)
C(19)-C(18)-H(18)	121.4	C(18)-C(19)-C(29)	123.0(18)
C(20)-C(19)-C(18)	119(2)	C(20)-C(19)-C(29)	117(2)
C(19)-C(20)-H(20)	119.8	C(19)-C(20)-C(21)	120(2)
C(21)-C(20)-H(20)	119.8	C(20)-C(21)-H(21)	119.6
C(20)-C(21)-C(22)	120.8(19)	C(22)-C(21)-H(21)	119.6
C(17)-C(22)-C(21)	117(2)	C(17)-C(22)-C(23)	113(2)
C(21)-C(22)-C(23)	129.6(19)	C(24)-C(23)-C(22)	134(2)
C(24)-C(23)-C(28)	120(2)	C(28)-C(23)-C(22)	105.3(18)
C(23)-C(24)-H(24)	120.0	C(23)-C(24)-C(25)	120(2)
C(25)-C(24)-H(24)	120.0	C(24)-C(25)-H(25)	119.1
C(26)-C(25)-C(24)	122(2)	C(26)-C(25)-H(25)	119.1
C(25)-C(26)-C(27)	114(2)	C(25)-C(26)-C(33)	122.9(19)
C(27)-C(26)-C(33)	123.3(18)	C(26)-C(27)-H(27)	117.5
C(28)-C(27)-C(26)	125(2)	C(28)-C(27)-H(27)	117.5
C(23)-C(28)-C(3)	111(2)	C(27)-C(28)-C(3)	128.6(19)
C(27)-C(28)-C(23)	119(2)	C(19)-C(29)-C(31)	108.4(16)
C(19)-C(29)-C(32)	112.0(18)	C(30)-C(29)-C(19)	117.2(17)
C(30)-C(29)-C(31)	106.7(17)	C(30)-C(29)-C(32)	109.2(16)
C(32)-C(29)-C(31)	102.1(15)	C(29)-C(30)-H(30A)	109.5
C(29)-C(30)-H(30B)	109.5	C(29)-C(30)-H(30C)	109.5
H(30A)-C(30)-H(30B)	109.5	H(30A)-C(30)-H(30C)	109.5

H(30B)-C(30)-H(30C)	109.5	C(29)-C(31)-H(31A)	109.5
C(29)-C(31)-H(31B)	109.5	C(29)-C(31)-H(31C)	109.5
H(31A)-C(31)-H(31B)	109.5	H(31A)-C(31)-H(31C)	109.5
H(31B)-C(31)-H(31C)	109.5	C(29)-C(32)-H(32A)	109.5
C(29)-C(32)-H(32B)	109.5	C(29)-C(32)-H(32C)	109.5
H(32A)-C(32)-H(32B)	109.5	H(32A)-C(32)-H(32C)	109.5
H(32B)-C(32)-H(32C)	109.5	C(26)-C(33)-C(34)	111.1(16)
C(26)-C(33)-C(35)	108.2(17)	C(34)-C(33)-C(35)	106.5(15)
C(36)-C(33)-C(26)	112.6(18)	C(36)-C(33)-C(34)	112.6(17)
C(36)-C(33)-C(35)	105.4(16)	C(33)-C(34)-H(34A)	109.5
C(33)-C(34)-H(34B)	109.5	C(33)-C(34)-H(34C)	109.5
H(34A)-C(34)-H(34B)	109.5	H(34A)-C(34)-H(34C)	109.5
H(34B)-C(34)-H(34C)	109.5	C(33)-C(35)-H(35A)	109.5
C(33)-C(35)-H(35B)	109.5	C(33)-C(35)-H(35C)	109.5
H(35A)-C(35)-H(35B)	109.5	H(35A)-C(35)-H(35C)	109.5
H(35B)-C(35)-H(35C)	109.5	C(33)-C(36)-H(36A)	109.5
C(33)-C(36)-H(36B)	109.5	C(33)-C(36)-H(36C)	109.5
H(36A)-C(36)-H(36B)	109.5	H(36A)-C(36)-H(36C)	109.5
H(36B)-C(36)-H(36C)	109.5	C(11)-C(37)-C(42)	106.9(19)
C(38)-C(37)-C(11)	133.9(18)	C(38)-C(37)-C(42)	117.4(18)
C(37)-C(38)-H(38)	115.5	C(37)-C(38)-C(39)	129.0(19)
C(39)-C(38)-H(38)	115.5	C(38)-C(39)-C(40)	108(2)
C(49)-C(39)-C(38)	126.8(18)	C(49)-C(39)-C(40)	123.3(18)
C(39)-C(40)-H(40)	116.4	C(41)-C(40)-C(39)	127.2(19)
C(41)-C(40)-H(40)	116.4	C(40)-C(41)-H(41)	119.6
C(40)-C(41)-C(42)	120.9(19)	C(42)-C(41)-H(41)	119.6
C(41)-C(42)-C(37)	116(2)	C(41)-C(42)-C(43)	139.6(19)
C(43)-C(42)-C(37)	104.7(17)	C(44)-C(43)-C(42)	128.5(18)
C(48)-C(43)-C(42)	111.5(19)	C(48)-C(43)-C(44)	119(2)
C(43)-C(44)-H(44)	120.1	C(45)-C(44)-C(43)	119.9(19)
C(45)-C(44)-H(44)	120.1	C(44)-C(45)-H(45)	120.3
C(44)-C(45)-C(46)	119(2)	C(46)-C(45)-H(45)	120.3
C(45)-C(46)-C(53)	114.2(19)	C(47)-C(46)-C(45)	118(2)
C(47)-C(46)-C(53)	123.1(19)	C(46)-C(47)-H(47)	119.5
C(48)-C(47)-C(46)	121.0(19)	C(48)-C(47)-H(47)	119.5
C(43)-C(48)-C(11)	109(2)	C(47)-C(48)-C(11)	131.0(19)

C(47)-C(48)-C(43)	119(2)	C(39)-C(49)-C(50)	112.3(17)
C(39)-C(49)-C(51)	113.7(19)	C(39)-C(49)-C(52)	116.7(18)
C(51)-C(49)-C(50)	104.5(16)	C(51)-C(49)-C(52)	107.2(15)
C(52)-C(49)-C(50)	100.9(16)	C(49)-C(50)-H(50A)	109.5
C(49)-C(50)-H(50B)	109.5	C(49)-C(50)-H(50C)	109.5
H(50A)-C(50)-H(50B)	109.5	H(50A)-C(50)-H(50C)	109.5
H(50B)-C(50)-H(50C)	109.5	C(49)-C(51)-H(51A)	109.5
C(49)-C(51)-H(51B)	109.5	C(49)-C(51)-H(51C)	109.5
H(51A)-C(51)-H(51B)	109.5	H(51A)-C(51)-H(51C)	109.5
H(51B)-C(51)-H(51C)	109.5	C(49)-C(52)-H(52A)	109.5
C(49)-C(52)-H(52B)	109.5	C(49)-C(52)-H(52C)	109.5
H(52A)-C(52)-H(52B)	109.5	H(52A)-C(52)-H(52C)	109.5
H(52B)-C(52)-H(52C)	109.5	C(46)-C(53)-C(54)	111.6(18)
C(46)-C(53)-C(56)	108.2(17)	C(54)-C(53)-C(56)	99.0(14)
C(55)-C(53)-C(46)	122.7(17)	C(55)-C(53)-C(54)	108.6(19)
C(55)-C(53)-C(56)	103.9(17)	C(53)-C(54)-H(54A)	109.5
C(53)-C(54)-H(54B)	109.5	C(53)-C(54)-H(54C)	109.5
H(54A)-C(54)-H(54B)	109.5	H(54A)-C(54)-H(54C)	109.5
H(54B)-C(54)-H(54C)	109.5	C(53)-C(55)-H(55A)	109.5
C(53)-C(55)-H(55B)	109.5	C(53)-C(55)-H(55C)	109.5
H(55A)-C(55)-H(55B)	109.5	H(55A)-C(55)-H(55C)	109.5
H(55B)-C(55)-H(55C)	109.5	C(53)-C(56)-H(56A)	109.5
C(53)-C(56)-H(56B)	109.5	C(53)-C(56)-H(56C)	109.5
H(56A)-C(56)-H(56B)	109.5	H(56A)-C(56)-H(56C)	109.5
H(56B)-C(56)-H(56C)	109.5	C(57)-C(58)-H(58)	120.0
C(57)-C(58)-C(59)	120.0	C(59)-C(58)-H(58)	120.0
N(1)-C(57)-C(58)	120.7(11)	N(1)-C(57)-C(62)	118.8(10)
C(58)-C(57)-C(62)	120.0	C(57)-C(62)-H(62)	120.0
C(57)-C(62)-C(61)	120.0	C(61)-C(62)-H(62)	120.0
C(62)-C(61)-H(61)	120.0	C(60)-C(61)-C(62)	120.0
C(60)-C(61)-H(61)	120.0	C(61)-C(60)-C(59)	120.0
C(61)-C(60)-C(63)	122.8(9)	C(59)-C(60)-C(63)	117.1(9)
C(58)-C(59)-H(59)	120.0	C(60)-C(59)-C(58)	120.0
C(60)-C(59)-H(59)	120.0	C(68)-C(63)-C(60)	119.6(10)
C(68)-C(63)-C(64)	120.0	C(64)-C(63)-C(60)	120.2(10)
C(63)-C(68)-H(68)	120.0	C(63)-C(68)-C(67)	120.0

C(67)-C(68)-H(68)	120.0	C(68)-C(67)-H(67)	120.0
C(68)-C(67)-C(66)	120.0	C(66)-C(67)-H(67)	120.0
C(67)-C(66)-H(66)	120.0	C(67)-C(66)-C(65)	120.0
C(65)-C(66)-H(66)	120.0	C(66)-C(65)-H(65)	120.0
C(64)-C(65)-C(66)	120.0	C(64)-C(65)-H(65)	120.0
C(63)-C(64)-H(64)	120.0	C(65)-C(64)-C(63)	120.0
C(65)-C(64)-H(64)	120.0	C(72)-C(73)-H(73)	120.0
C(72)-C(73)-C(74)	120.0	C(74)-C(73)-H(73)	120.0
C(73)-C(72)-C(71)	120.0	C(73)-C(72)-C(75)	118.8(9)
C(71)-C(72)-C(75)	121.1(9)	C(72)-C(71)-H(71)	120.0
C(72)-C(71)-C(70)	120.0	C(70)-C(71)-H(71)	120.0
C(71)-C(70)-H(70)	120.0	C(71)-C(70)-C(69)	120.0
C(69)-C(70)-H(70)	120.0	N(4)-C(69)-C(70)	122.2(11)
N(4)-C(69)-C(74)	117.8(11)	C(74)-C(69)-C(70)	120.0
C(73)-C(74)-H(74)	120.0	C(69)-C(74)-C(73)	120.0
C(69)-C(74)-H(74)	120.0	C(80)-C(75)-C(72)	118.2(9)
C(80)-C(75)-C(76)	120.0	C(76)-C(75)-C(72)	121.8(9)
C(75)-C(80)-H(80)	120.0	C(79)-C(80)-C(75)	120.0
C(79)-C(80)-H(80)	120.0	C(80)-C(79)-H(79)	120.0
C(80)-C(79)-C(78)	120.0	C(78)-C(79)-H(79)	120.0
C(79)-C(78)-H(78)	120.0	C(77)-C(78)-C(79)	120.0
C(77)-C(78)-H(78)	120.0	C(78)-C(77)-H(77)	120.0
C(78)-C(77)-C(76)	120.0	C(76)-C(77)-H(77)	120.0
C(75)-C(76)-H(76)	120.0	C(77)-C(76)-C(75)	120.0
C(77)-C(76)-H(76)	120.0	N(5)-Bi(2)-N(8)	75.2(10)
N(5)-Bi(2)-C(81)	107.4(9)	N(8)-Bi(2)-C(81)	104.7(8)
N(6)-N(5)-Bi(2)	116(2)	N(6)-N(5)-C(137)	109(2)
C(137)-N(5)-Bi(2)	133.4(15)	N(7)-N(6)-N(5)	116(2)
N(6)-N(7)-N(8)	121(2)	N(7)-N(8)-Bi(2)	110(2)
C(149)-N(8)-Bi(2)	125.9(16)	C(149)-N(8)-N(7)	118(2)
C(82)-C(81)-Bi(2)	109.6(14)	C(92)-C(81)-Bi(2)	133.1(14)
C(92)-C(81)-C(82)	117.3(18)	C(81)-C(82)-C(83)	128.1(18)
C(86)-C(82)-C(81)	118.3(18)	C(86)-C(82)-C(83)	112.9(17)
C(82)-C(83)-C(84)	100.0(15)	C(82)-C(83)-C(97)	118(2)
C(82)-C(83)-C(108)	117.0(19)	C(97)-C(83)-C(84)	109.9(17)
C(97)-C(83)-C(108)	105.5(18)	C(108)-C(83)-C(84)	106.0(18)

C(83)-C(84)-H(84A)	110.5	C(83)-C(84)-H(84B)	110.5
H(84A)-C(84)-H(84B)	108.7	C(85)-C(84)-C(83)	106.0(16)
C(85)-C(84)-H(84A)	110.5	C(85)-C(84)-H(84B)	110.5
C(84)-C(85)-C(86)	108.8(17)	C(84)-C(85)-C(93)	116.4(19)
C(84)-C(85)-C(94)	112.2(19)	C(86)-C(85)-C(93)	109.3(19)
C(86)-C(85)-C(94)	108.7(17)	C(93)-C(85)-C(94)	101.1(16)
C(82)-C(86)-C(85)	108.5(17)	C(87)-C(86)-C(82)	124.3(19)
C(87)-C(86)-C(85)	127.1(19)	C(86)-C(87)-H(87)	119.6
C(86)-C(87)-C(88)	121(2)	C(88)-C(87)-H(87)	119.6
C(89)-C(88)-C(87)	126.7(19)	C(92)-C(88)-C(87)	117.2(18)
C(92)-C(88)-C(89)	115.1(18)	C(88)-C(89)-C(90)	104.9(17)
C(88)-C(89)-C(95)	118(2)	C(88)-C(89)-C(96)	108(2)
C(90)-C(89)-C(96)	101.6(17)	C(95)-C(89)-C(90)	118(2)
C(95)-C(89)-C(96)	105.5(18)	C(89)-C(90)-H(90A)	110.8
C(89)-C(90)-H(90B)	110.8	C(89)-C(90)-C(91)	104.8(15)
H(90A)-C(90)-H(90B)	108.9	C(91)-C(90)-H(90A)	110.8
C(91)-C(90)-H(90B)	110.8	C(92)-C(91)-C(90)	100.9(15)
C(92)-C(91)-C(128)	119(2)	C(117)-C(91)-C(90)	111.9(19)
C(117)-C(91)-C(92)	122(2)	C(117)-C(91)-C(128)	102.5(17)
C(128)-C(91)-C(90)	97.9(16)	C(81)-C(92)-C(91)	127.0(18)
C(88)-C(92)-C(81)	121.8(18)	C(88)-C(92)-C(91)	111.1(17)
C(85)-C(93)-H(93A)	109.5	C(85)-C(93)-H(93B)	109.5
C(85)-C(93)-H(93C)	109.5	H(93A)-C(93)-H(93B)	109.5
H(93A)-C(93)-H(93C)	109.5	H(93B)-C(93)-H(93C)	109.5
C(85)-C(94)-H(94A)	109.5	C(85)-C(94)-H(94B)	109.5
C(85)-C(94)-H(94C)	109.5	H(94A)-C(94)-H(94B)	109.5
H(94A)-C(94)-H(94C)	109.5	H(94B)-C(94)-H(94C)	109.5
C(89)-C(95)-H(95A)	109.5	C(89)-C(95)-H(95B)	109.5
C(89)-C(95)-H(95C)	109.5	H(95A)-C(95)-H(95B)	109.5
H(95A)-C(95)-H(95C)	109.5	H(95B)-C(95)-H(95C)	109.5
C(89)-C(96)-H(96A)	109.5	C(89)-C(96)-H(96B)	109.5
C(89)-C(96)-H(96C)	109.5	H(96A)-C(96)-H(96B)	109.5
H(96A)-C(96)-H(96C)	109.5	H(96B)-C(96)-H(96C)	109.5
C(98)-C(97)-C(83)	128(2)	C(98)-C(97)-C(102)	125(2)
C(102)-C(97)-C(83)	107(2)	C(97)-C(98)-H(98)	122.2
C(99)-C(98)-C(97)	116(2)	C(99)-C(98)-H(98)	122.2

C(98)-C(99)-C(100)	123(2)	C(98)-C(99)-C(109)	117(2)
C(100)-C(99)-C(109)	120(2)	C(99)-C(100)-H(100)	120.5
C(101)-C(100)-C(99)	119(2)	C(101)-C(100)-H(100)	120.5
C(100)-C(101)-H(101)	119.7	C(102)-C(101)-C(100)	121(2)
C(102)-C(101)-H(101)	119.7	C(101)-C(102)-C(97)	116(2)
C(101)-C(102)-C(103)	136(2)	C(103)-C(102)-C(97)	108(2)
C(102)-C(103)-C(108)	114(2)	C(104)-C(103)-C(102)	129(2)
C(104)-C(103)-C(108)	116(2)	C(103)-C(104)-H(104)	119.0
C(103)-C(104)-C(105)	122(2)	C(105)-C(104)-H(104)	119.0
C(104)-C(105)-H(105)	121.3	C(106)-C(105)-C(104)	117(2)
C(106)-C(105)-H(105)	121.3	C(105)-C(106)-C(107)	119(2)
C(105)-C(106)-C(113)	122(2)	C(107)-C(106)-C(113)	118.8(19)
C(106)-C(107)-H(107)	119.4	C(108)-C(107)-C(106)	121(2)
C(108)-C(107)-H(107)	119.4	C(103)-C(108)-C(83)	105(2)
C(107)-C(108)-C(83)	131(2)	C(107)-C(108)-C(103)	124(2)
C(99)-C(109)-C(110)	117.7(18)	C(99)-C(109)-C(111)	110.3(19)
C(99)-C(109)-C(112)	115.7(18)	C(111)-C(109)-C(110)	104.7(17)
C(111)-C(109)-C(112)	104.2(17)	C(112)-C(109)-C(110)	102.9(18)
C(109)-C(110)-H(11A)	109.5	C(109)-C(110)-H(11B)	109.5
C(109)-C(110)-H(11C)	109.5	H(11A)-C(110)-H(11B)	109.5
H(11A)-C(110)-H(11C)	109.5	H(11B)-C(110)-H(11C)	109.5
C(109)-C(111)-H(11D)	109.5	C(109)-C(111)-H(11E)	109.5
C(109)-C(111)-H(11F)	109.5	H(11D)-C(111)-H(11E)	109.5
H(11D)-C(111)-H(11F)	109.5	H(11E)-C(111)-H(11F)	109.5
C(109)-C(112)-H(11G)	109.5	C(109)-C(112)-H(11H)	109.5
C(109)-C(112)-H(11I)	109.5	H(11G)-C(112)-H(11H)	109.5
H(11G)-C(112)-H(11I)	109.5	H(11H)-C(112)-H(11I)	109.5
C(106)-C(113)-C(115)	102.9(16)	C(114)-C(113)-C(106)	107.5(18)
C(114)-C(113)-C(115)	104.9(17)	C(116)-C(113)-C(106)	116(2)
C(116)-C(113)-C(114)	117(2)	C(116)-C(113)-C(115)	107.8(18)
C(113)-C(114)-H(11J)	109.5	C(113)-C(114)-H(11K)	109.5
C(113)-C(114)-H(11L)	109.5	H(11J)-C(114)-H(11K)	109.5
H(11J)-C(114)-H(11L)	109.5	H(11K)-C(114)-H(11L)	109.5
C(113)-C(115)-H(11M)	109.5	C(113)-C(115)-H(11N)	109.5
C(113)-C(115)-H(11O)	109.5	H(11M)-C(115)-H(11N)	109.5
H(11M)-C(115)-H(11O)	109.5	H(11N)-C(115)-H(11O)	109.5

C(113)-C(116)-H(11P)	109.5	C(113)-C(116)-H(11Q)	109.5
C(113)-C(116)-H(11R)	109.5	H(11P)-C(116)-H(11Q)	109.5
H(11P)-C(116)-H(11R)	109.5	H(11Q)-C(116)-H(11R)	109.5
C(118)-C(117)-C(91)	128(2)	C(118)-C(117)-C(122)	121(2)
C(122)-C(117)-C(91)	110(2)	C(117)-C(118)-H(118)	117.9
C(117)-C(118)-C(119)	124(2)	C(119)-C(118)-H(118)	117.9
C(118)-C(119)-C(129)	122.9(19)	C(120)-C(119)-C(118)	113(2)
C(120)-C(119)-C(129)	124(2)	C(119)-C(120)-H(120)	117.0
C(119)-C(120)-C(121)	126(2)	C(121)-C(120)-H(120)	117.0
C(120)-C(121)-H(121)	122.0	C(120)-C(121)-C(122)	116(2)
C(122)-C(121)-H(121)	122.0	C(117)-C(122)-C(121)	119(2)
C(123)-C(122)-C(117)	111(2)	C(123)-C(122)-C(121)	129(2)
C(122)-C(123)-C(124)	132(2)	C(122)-C(123)-C(128)	110(2)
C(128)-C(123)-C(124)	118(2)	C(123)-C(124)-H(124)	121.2
C(125)-C(124)-C(123)	118(2)	C(125)-C(124)-H(124)	121.2
C(124)-C(125)-H(125)	117.7	C(126)-C(125)-C(124)	125(2)
C(126)-C(125)-H(125)	117.7	C(125)-C(126)-C(127)	119(2)
C(125)-C(126)-C(133)	120(2)	C(127)-C(126)-C(133)	119.8(19)
C(126)-C(127)-H(127)	121.3	C(126)-C(127)-C(128)	117(2)
C(128)-C(127)-H(127)	121.3	C(123)-C(128)-C(91)	106(2)
C(123)-C(128)-C(127)	123(2)	C(127)-C(128)-C(91)	130.7(18)
C(130)-C(129)-C(119)	111(2)	C(130)-C(129)-C(131)	101.7(17)
C(131)-C(129)-C(119)	110.3(19)	C(132)-C(129)-C(119)	106.0(18)
C(132)-C(129)-C(130)	107.1(18)	C(132)-C(129)-C(131)	121(2)
C(129)-C(130)-H(13D)	109.5	C(129)-C(130)-H(13E)	109.5
C(129)-C(130)-H(13F)	109.5	H(13D)-C(130)-H(13E)	109.5
H(13D)-C(130)-H(13F)	109.5	H(13E)-C(130)-H(13F)	109.5
C(129)-C(131)-H(13G)	109.5	C(129)-C(131)-H(13H)	109.5
C(129)-C(131)-H(13I)	109.5	H(13G)-C(131)-H(13H)	109.5
H(13G)-C(131)-H(13I)	109.5	H(13H)-C(131)-H(13I)	109.5
C(129)-C(132)-H(13J)	109.5	C(129)-C(132)-H(13K)	109.5
C(129)-C(132)-H(13L)	109.5	H(13J)-C(132)-H(13K)	109.5
H(13J)-C(132)-H(13L)	109.5	H(13K)-C(132)-H(13L)	109.5
C(126)-C(133)-C(135)	104.5(18)	C(126)-C(133)-C(136)	107.7(17)
C(134)-C(133)-C(126)	110.1(19)	C(134)-C(133)-C(135)	111.4(17)
C(134)-C(133)-C(136)	113(2)	C(135)-C(133)-C(136)	109.5(17)

C(133)-C(134)-H(13M)	109.5	C(133)-C(134)-H(13N)	109.5
C(133)-C(134)-H(13O)	109.5	H(13M)-C(134)-H(13N)	109.5
H(13M)-C(134)-H(13O)	109.5	H(13N)-C(134)-H(13O)	109.5
C(133)-C(135)-H(13P)	109.5	C(133)-C(135)-H(13Q)	109.5
C(133)-C(135)-H(13R)	109.5	H(13P)-C(135)-H(13Q)	109.5
H(13P)-C(135)-H(13R)	109.5	H(13Q)-C(135)-H(13R)	109.5
C(133)-C(136)-H(13S)	109.5	C(133)-C(136)-H(13T)	109.5
C(133)-C(136)-H(13U)	109.5	H(13S)-C(136)-H(13T)	109.5
H(13S)-C(136)-H(13U)	109.5	H(13T)-C(136)-H(13U)	109.5
C(142)-C(137)-N(5)	116.3(13)	C(142)-C(137)-C(138)	120.0
C(138)-C(137)-N(5)	122.7(13)	C(137)-C(142)-H(142)	120.0
C(137)-C(142)-C(141)	120.0	C(141)-C(142)-H(142)	120.0
C(142)-C(141)-H(141)	120.0	C(140)-C(141)-C(142)	120.0
C(140)-C(141)-H(141)	120.0	C(141)-C(140)-C(139)	120.0
C(141)-C(140)-C(143)	118.8(12)	C(139)-C(140)-C(143)	119.0(12)
C(140)-C(139)-H(139)	120.0	C(140)-C(139)-C(138)	120.0
C(138)-C(139)-H(139)	120.0	C(137)-C(138)-H(138)	120.0
C(139)-C(138)-C(137)	120.0	C(139)-C(138)-H(138)	120.0
C(148)-C(143)-C(140)	120.2(12)	C(148)-C(143)-C(144)	120.0
C(144)-C(143)-C(140)	119.1(12)	C(143)-C(148)-H(148)	120.0
C(143)-C(148)-C(147)	120.0	C(147)-C(148)-H(148)	120.0
C(148)-C(147)-H(147)	120.0	C(146)-C(147)-C(148)	120.0
C(146)-C(147)-H(147)	120.0	C(147)-C(146)-H(146)	120.0
C(145)-C(146)-C(147)	120.0	C(145)-C(146)-H(146)	120.0
C(146)-C(145)-H(145)	120.0	C(146)-C(145)-C(144)	120.0
C(144)-C(145)-H(145)	120.0	C(143)-C(144)-H(144)	120.0
C(145)-C(144)-C(143)	120.0	C(145)-C(144)-H(144)	120.0
C(149)-C(154)-H(154)	120.0	C(149)-C(154)-C(153)	120.0
C(153)-C(154)-H(154)	120.0	N(8)-C(149)-C(154)	119.4(14)
N(8)-C(149)-C(150)	120.5(14)	C(150)-C(149)-C(154)	120.0
C(149)-C(150)-H(150)	120.0	C(151)-C(150)-C(149)	120.0
C(151)-C(150)-H(150)	120.0	C(150)-C(151)-H(151)	120.0
C(150)-C(151)-C(152)	120.0	C(152)-C(151)-H(151)	120.0
C(151)-C(152)-C(153)	120.0	C(151)-C(152)-C(155)	114.1(12)
C(153)-C(152)-C(155)	125.7(12)	C(154)-C(153)-H(153)	120.0
C(152)-C(153)-C(154)	120.0	C(152)-C(153)-H(153)	120.0

C(160)-C(155)-C(152)	113.6(12)	C(160)-C(155)-C(156)	120.0
C(156)-C(155)-C(152)	126.3(12)	C(155)-C(160)-H(160)	120.0
C(159)-C(160)-C(155)	120.0	C(159)-C(160)-H(160)	120.0
C(160)-C(159)-H(159)	120.0	C(160)-C(159)-C(158)	120.0
C(158)-C(159)-H(159)	120.0	C(159)-C(158)-H(158)	120.0
C(157)-C(158)-C(159)	120.0	C(157)-C(158)-H(158)	120.0
C(158)-C(157)-H(157)	120.0	C(156)-C(157)-C(158)	120.0
C(156)-C(157)-H(157)	120.0	C(155)-C(156)-H(156)	120.0
C(157)-C(156)-C(155)	120.0	C(157)-C(156)-H(156)	120.0

4. Reactivity and Catalysis

4.1. Reduction of 2 with pinacolborane

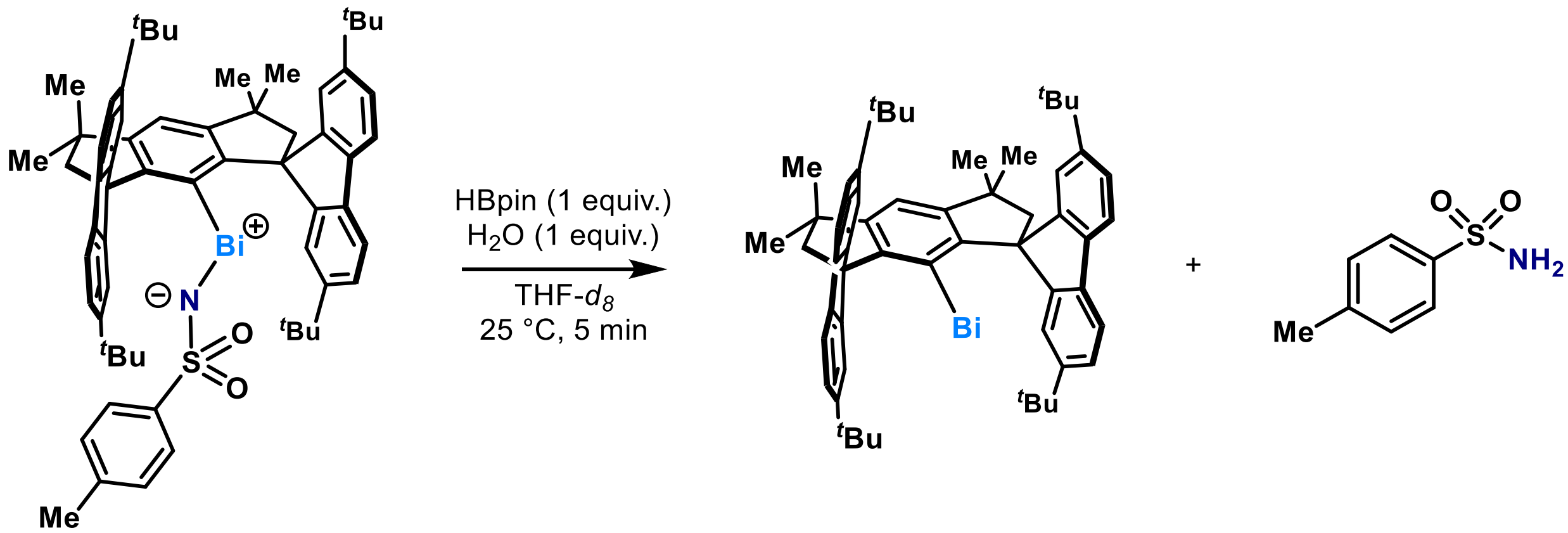

1 equiv. >99% 95%

Procedure: In an argon-filled glove box, **2** (10 mg, 0.0090 mmol, 1.0 equiv.) was weighed in an oven-dried 12 mL vial, followed by the addition of 0.5 mL of THF-d_8. Pinacolborane (1.3 µL, 0.0090 mmol, 1.0 equiv.) and 1,3,5-trimethoxybenzene (2.50 mg, 0.0149 mmol, 1.66 equiv.) were added, and the solution was allowed to stir for 5 min at 25 °C. The reaction mixture was transferred to an NMR tube, and the tube was capped with a septum. The tube was removed from the glovebox, and 6.8 µL of a 1.4 M solution of distilled water in THF-d_8 (0.0090 mmol, 1.0 equiv.) was added to the tube via microsyringe. The mixture was then shaken and the NMR spectrum recorded.

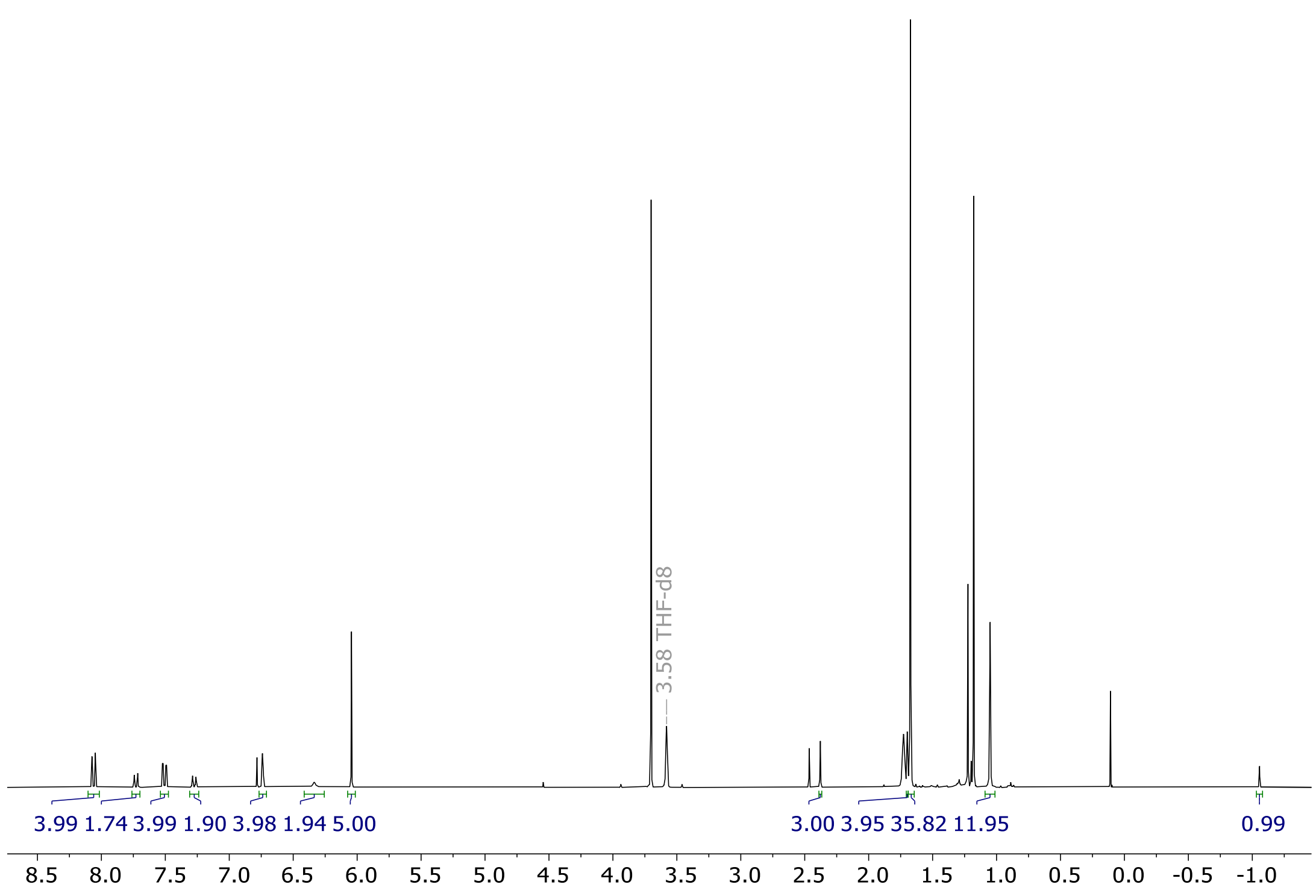

Figure S38. ^{1}H NMR spectrum displaying the quantitative reduction of **2** to *p*-toluenesulfonamide and full recovery of **1**.

4.2. Reduction of 3 with pinacolborane

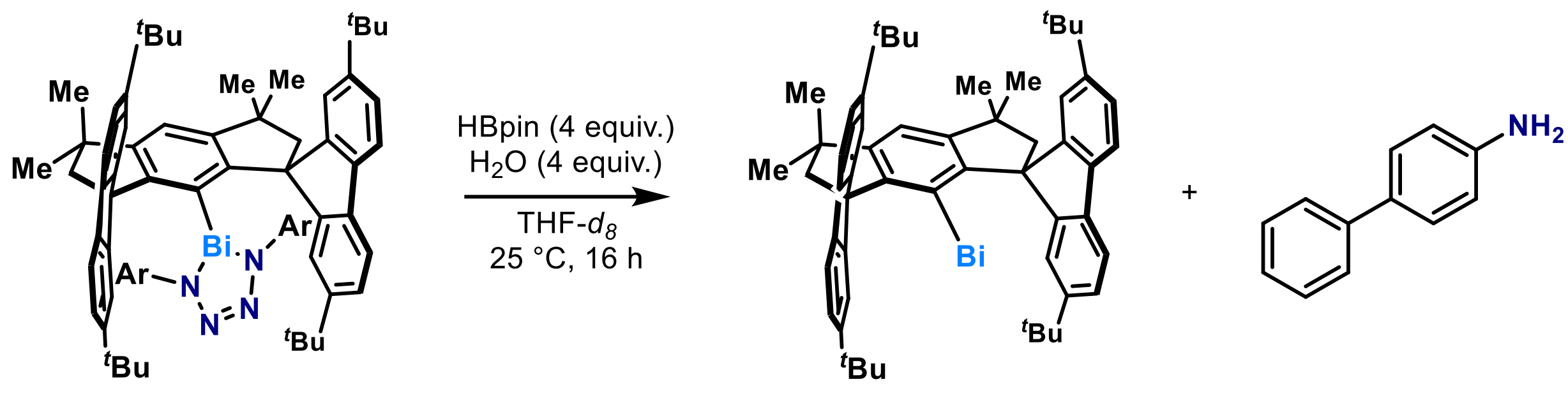

Procedure: In an argon-filled glove box, **3** (10 mg, 0.0076 mmol, 1.0 equiv.) was weighed in an oven-dried 12 mL vial, followed by the addition of 0.5 mL of THF-d_8. Pinacolborane (4.4 µL, 0.031 mmol, 4.0 equiv.) and 1,3,5-trimethoxybenzene (4.50 mg, 0.0268 mmol, 3.50 equiv.) were added, and the solution was allowed to stir for 5 min at 25 °C. The reaction mixture was transferred to an NMR tube, and the tube was capped with a septum. The tube was removed from the glovebox, and 22 µL of a 1.4 M solution of distilled water in THF-d_8 (0.031 mmol, 4.0 equiv.) was added to the tube via microsyringe. The mixture was then shaken and the NMR spectrum recorded after 16 h.

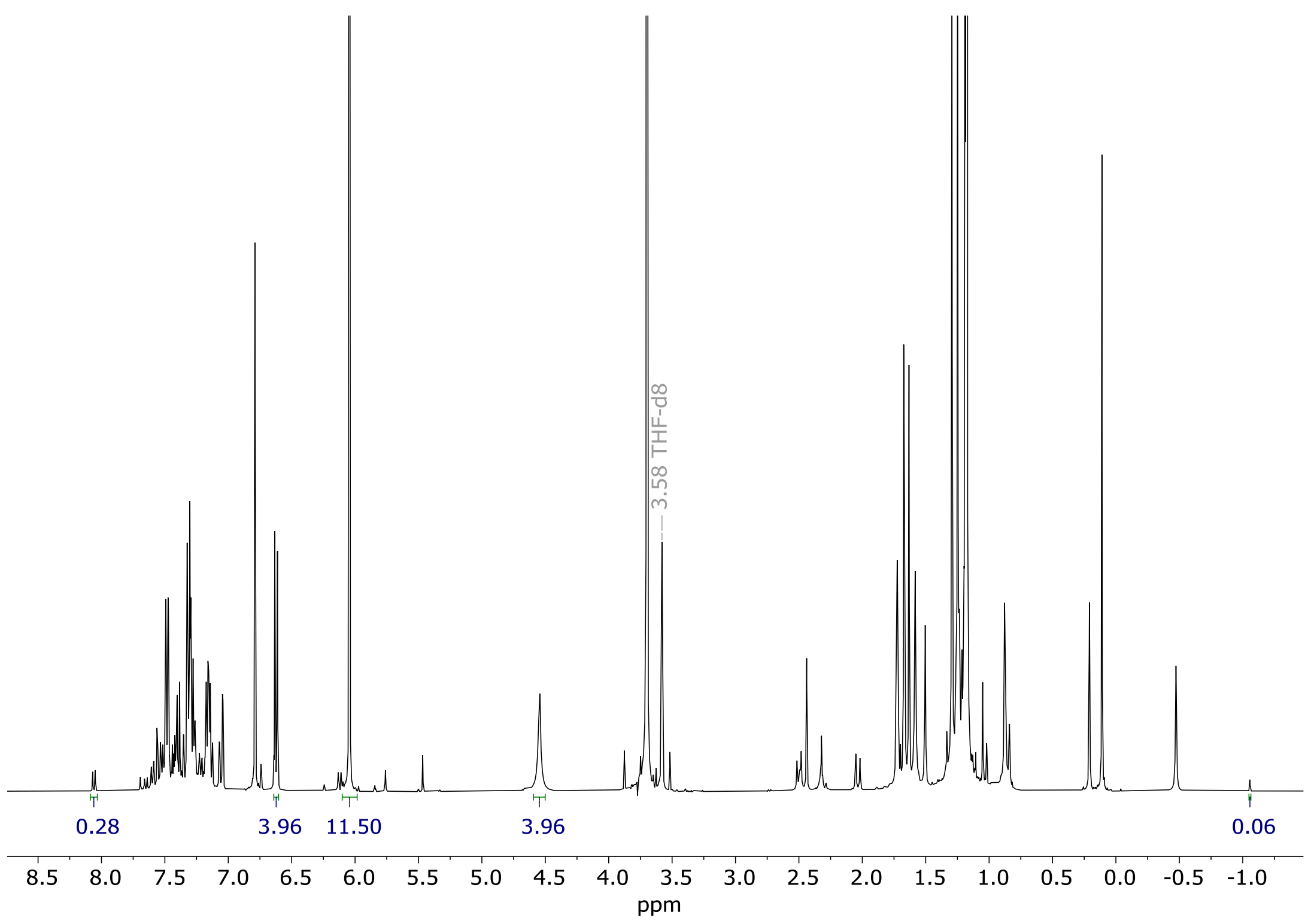

Figure S39. ^{1}H NMR spectrum displaying the quantitative reduction of **3** to 4-aminobiphenyl and partial recovery of **1**.

4.3. Catalytic reduction of *p*-toluenesulfonyl azide

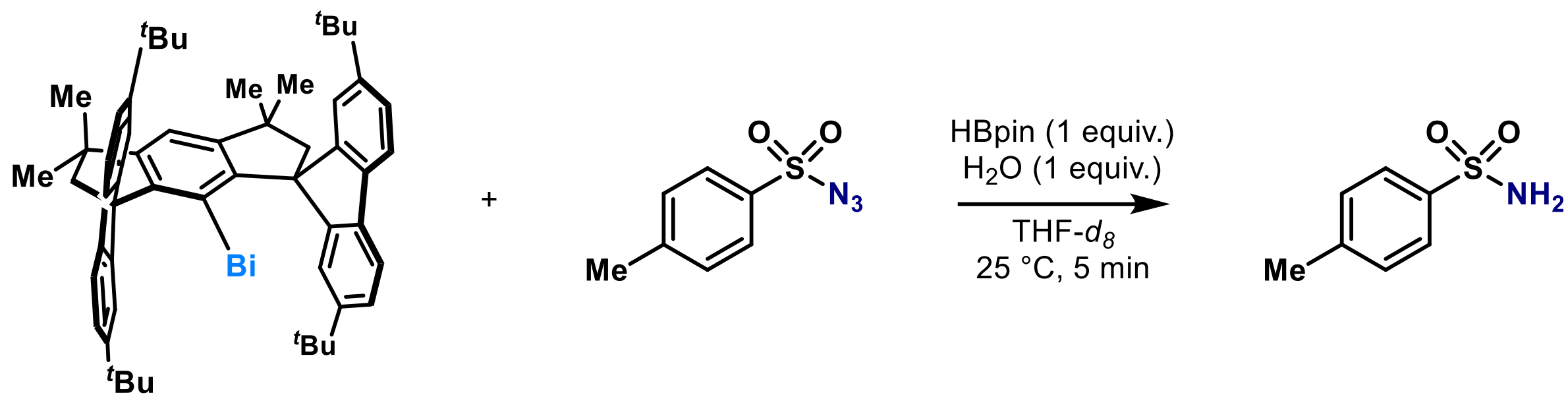

5 mol% 1 equiv. >99%

Procedure: In an argon-filled glove box, **1** (5.0 mg, 0.0053 mmol, 5.0 mol%) was weighed in an oven-dried 12 mL vial, followed by the addition of 0.5 mL of THF-d_8. *p*-Toluenesulfonyl azide (21.0 mg, 0.106 mmol, 1.00 equiv.), pinacolborane (15.4 μL, 0.106 mmol, 1.00 equiv.) and 1,3,5-trimethoxybenzene (4.44 mg, 0.0264 mmol, 0.250 equiv.) were added, and the solution was allowed to stir for 5 min at 25 °C. The reaction mixture was transferred to an NMR tube, and the tube was capped with a septum. The tube was removed from the glovebox, and 76 μL of a 1.4 M solution of distilled water in THF-d_8 (0.11 mmol, 1.0 equiv.) was added to the tube via microsyringe. The mixture was then shaken and the NMR spectrum recorded.

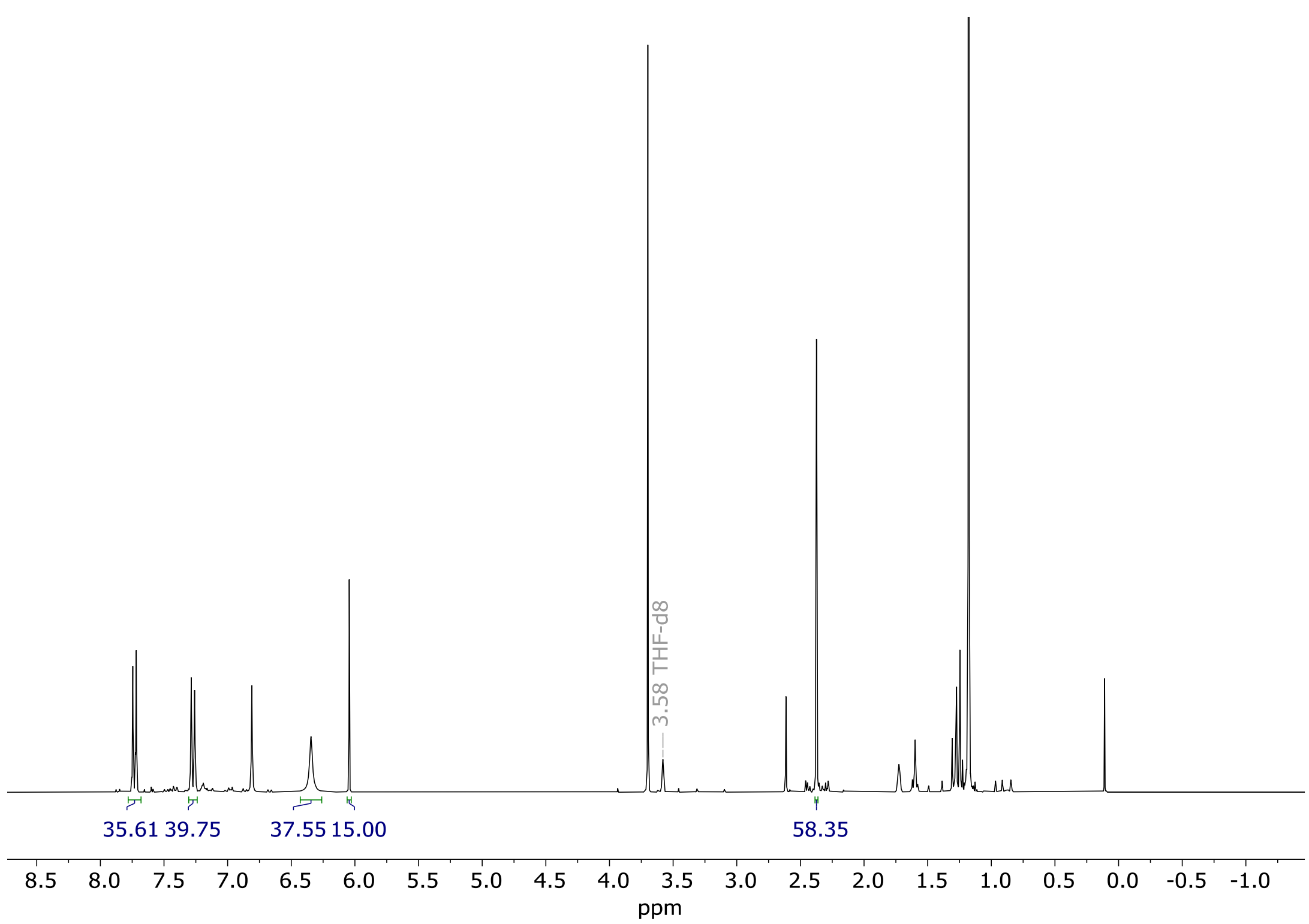

Figure S40. ^{1}H NMR spectrum displaying the catalytic reduction of *p*-toluenesulfonyl azide to *p*-toluenesulfonamide in quantitative yield (>99%).

4.4. Catalytic reduction of 4-acetamidobenzenesulfonyl azide

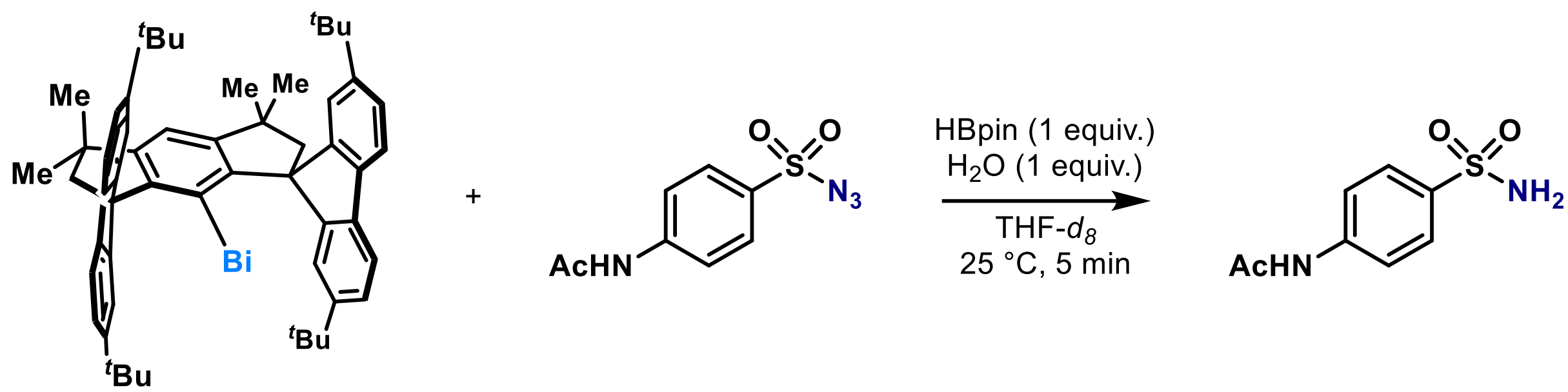

5 mol% **1 equiv.** **>99%**

Procedure: In an argon-filled glove box, **1** (5.0 mg, 0.0053 mmol, 5.0 mol%) was weighed in an oven-dried 12 mL vial, followed by the addition of 0.5 mL of THF-d_8. 4-Acetamidobenzenesulfonyl azide (25.4 mg, 0.106 mmol, 1.00 equiv.), pinacolborane (15.4 μL, 0.106 mmol, 1.00 equiv.) and 1,3,5-trimethoxybenzene (4.44 mg, 0.0264 mmol, 0.250 equiv.) were added, and the solution was allowed to stir for 5 min at 25 °C. The reaction mixture was transferred to an NMR tube, and the tube was capped with a septum. The tube was removed from the glovebox, and 76 μL of a 1.4 M solution of distilled water in THF-d_8 (0.11 mmol, 1.0 equiv.) was added to the tube via microsyringe. The mixture was then shaken and the NMR spectrum recorded.

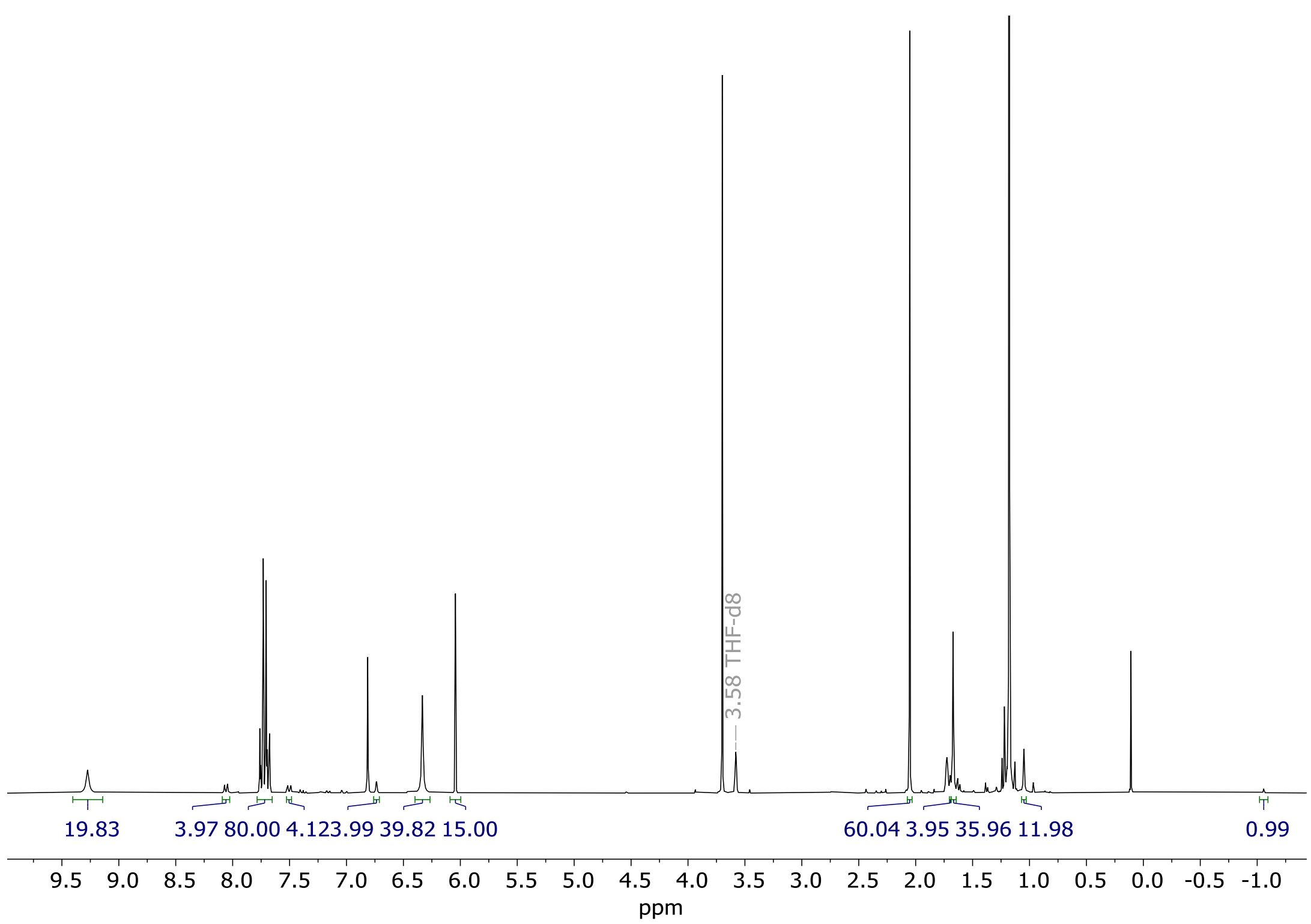

Figure S41. ^{1}H NMR spectrum displaying the catalytic reduction of 4-acetamidobenzenesulfonyl azide to 4-acetamidobenzenesulfonamide in quantitative yield (>99%), as well as complete recovery of Bi catalyst **1** (>99%).

4.5. Catalytic reduction of 4-azidobiphenyl

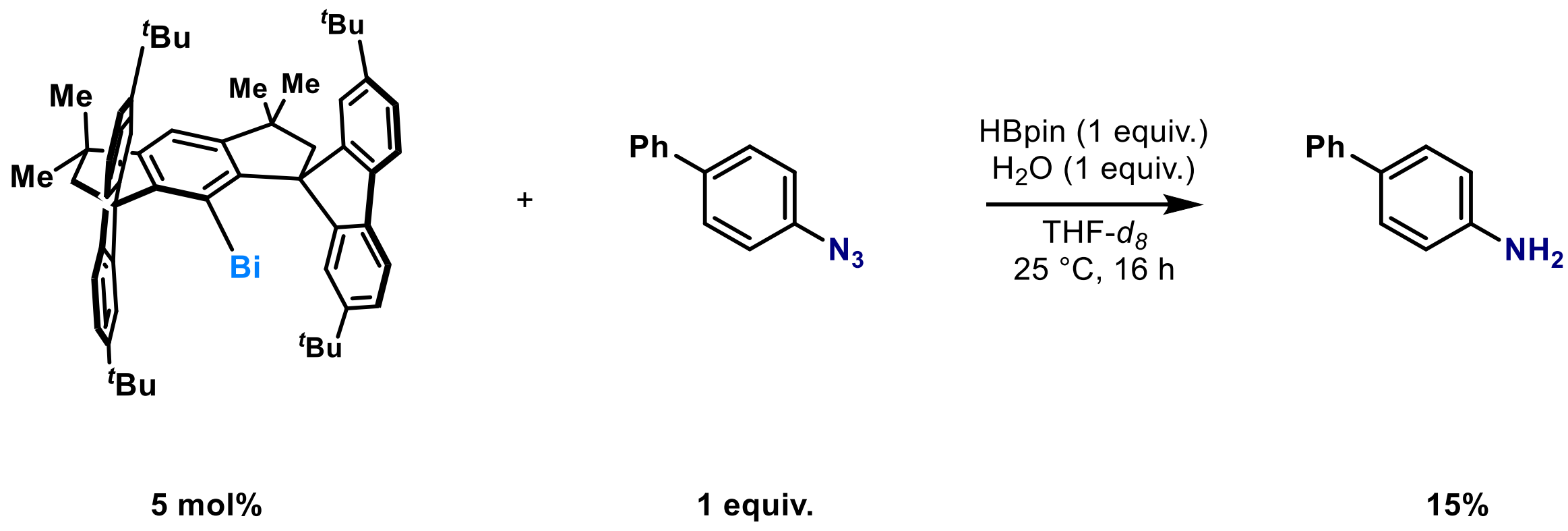

Procedure: In an argon-filled glove box, **1** (5.0 mg, 0.0053 mmol, 5.0 mol%) was weighed in an oven-dried 12 mL vial, followed by the addition of 0.5 mL of THF-d_8. 4-Azidobiphenyl (20.6 mg, 0.106 mmol, 1.00 equiv.), pinacolborane (15.4 µL, 0.106 mmol, 1.00 equiv.) and 1,3,5-trimethoxybenzene (2.00 mg, 0.0119 mmol, 0.112 equiv.) were added, and the solution was allowed to stir for 5 min at 25 °C. The reaction mixture was transferred to an NMR tube, and the tube was capped with a septum. The tube was removed from the glovebox, and 76 µL of a 1.4 M solution of distilled water in THF-d_8 (0.11 mmol, 1.0 equiv.) was added to the tube via microsyringe. The mixture was then shaken and the NMR spectrum recorded after 16 h.

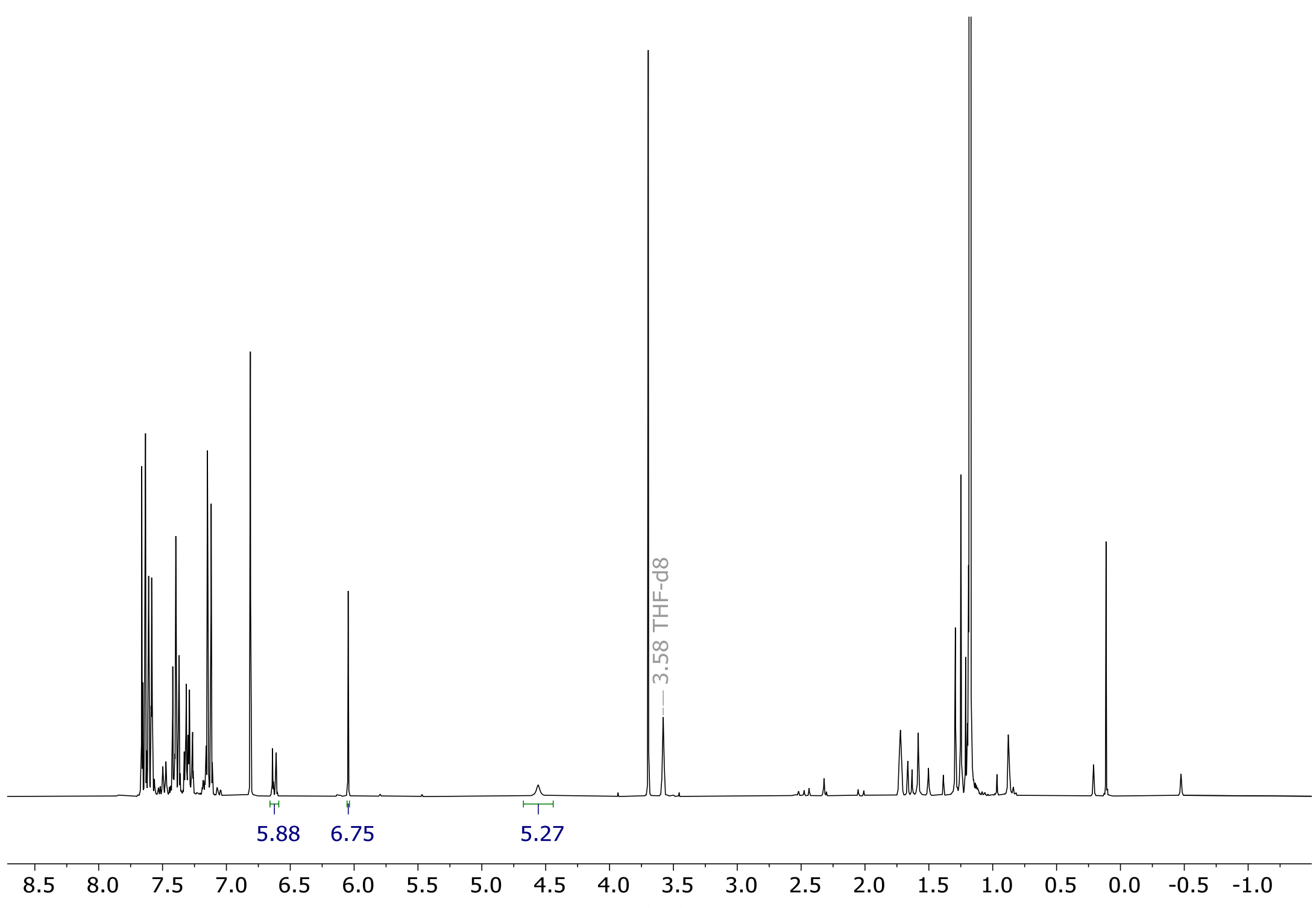

Figure S42. ^{1}H NMR spectrum displaying the catalytic reduction of 4-azidobiphenyl to 4-aminobiphenyl in 15% yield after 16 h.

4.6. Catalytic reduction of 1-azido-4-(trifluoromethyl)benzene

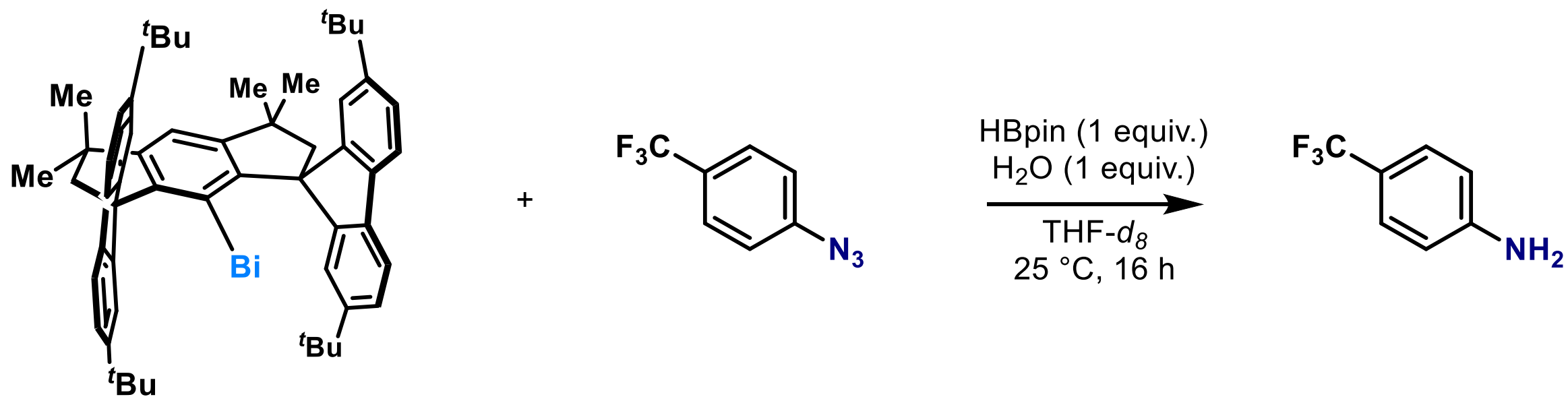

5 mol% **1 equiv.** **75%**

Procedure: In an argon-filled glove box, **1** (5.0 mg, 0.0053 mmol, 5.0 mol%) was weighed in an oven-dried 12 mL vial, followed by the addition of 0.5 mL of THF-d_8. 1-Azido-4-(trifluoromethyl)benzene (19.8 mg, 0.106 mmol, 1.00 equiv.), pinacolborane (15.4 µL, 0.106 mmol, 1.00 equiv.) and 1,3,5-trimethoxybenzene (2.00 mg, 0.0119 mmol, 0.112 equiv.) were added, and the solution was allowed to stir for 5 min at 25 °C. The reaction mixture was transferred to an NMR tube, and the tube was capped with a septum. The tube was removed from the glovebox, and 76 µL of a 1.4 M solution of distilled water in THF-d_8 (0.11 mmol, 1.0 equiv.) was added to the tube via microsyringe. The mixture was then shaken and the NMR spectrum recorded after 16 h.

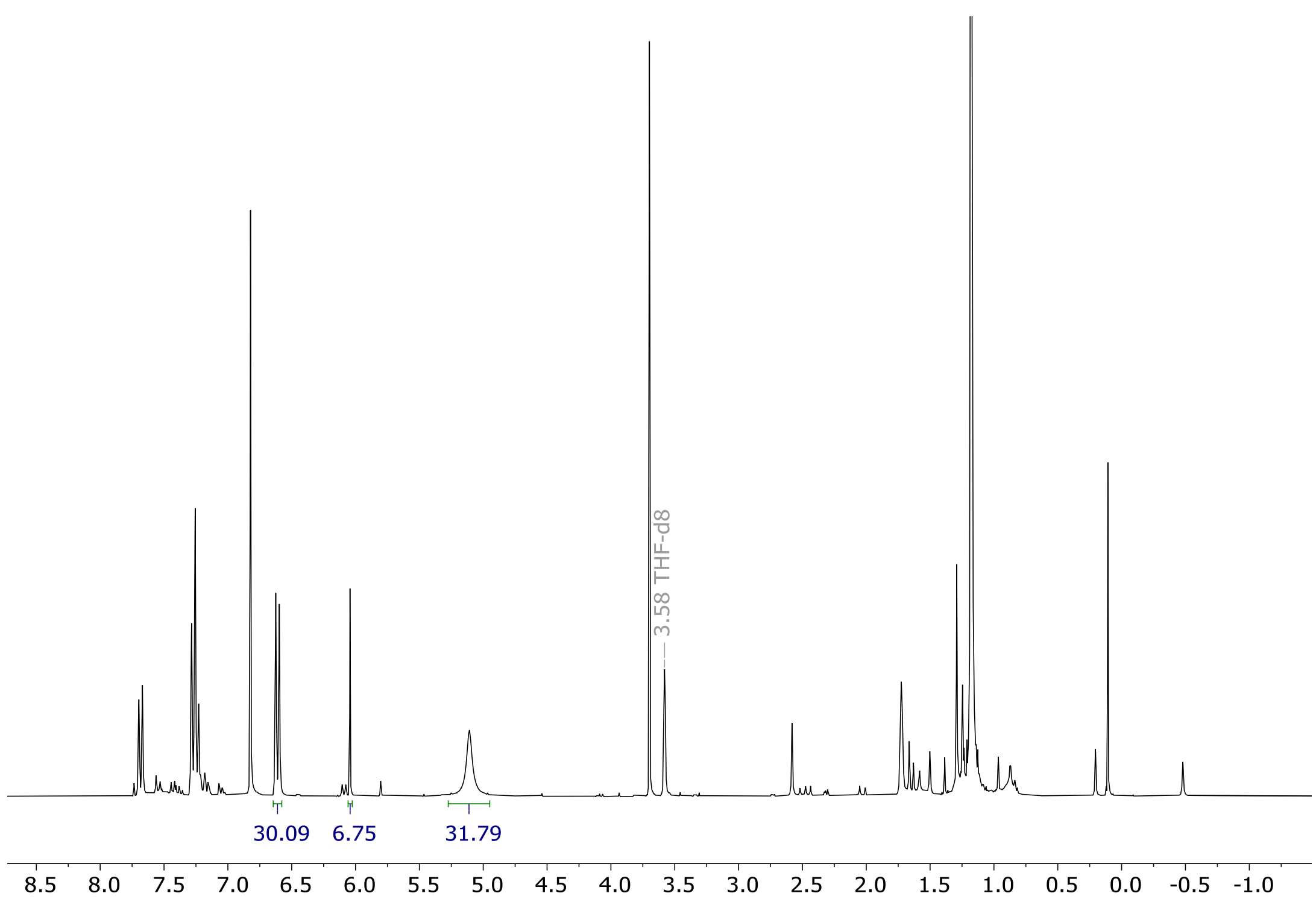

Figure S43. ^{1}H NMR spectrum displaying the catalytic reduction of 1-azido-4-(trifluoromethyl)benzene to 4-(trifluoromethyl)aniline in 75% yield after 16 h.

4.7. Catalytic reduction of 1-azido-4-methoxybenzene

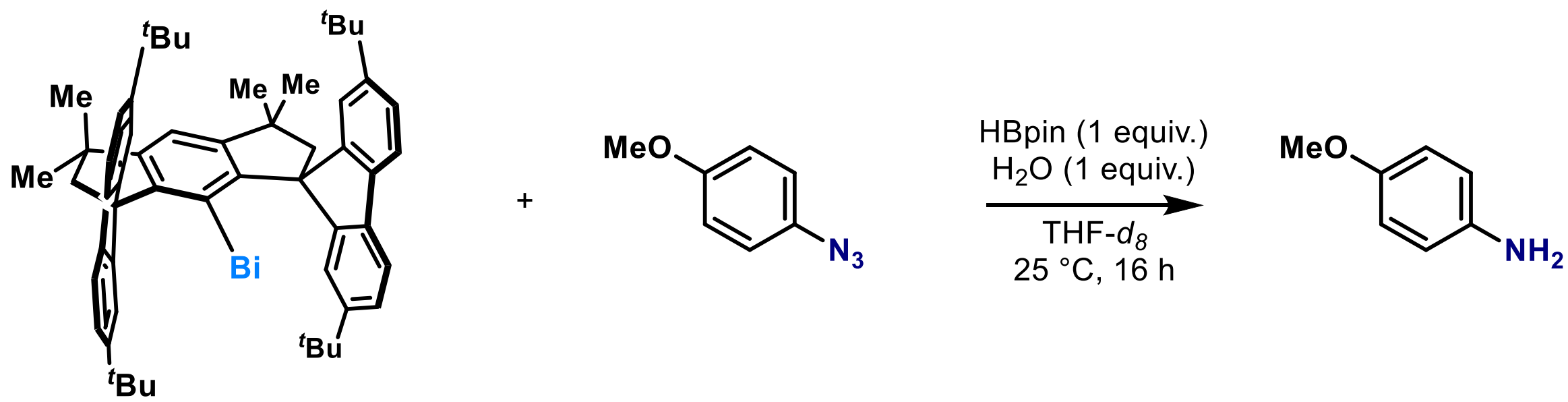

5 mol% **1 equiv.** **47%**

Procedure: In an argon-filled glove box, **1** (5.0 mg, 0.0053 mmol, 5.0 mol%) was weighed in an oven-dried 12 mL vial, followed by the addition of 0.5 mL of THF-d_8. 1-Azido-4-methoxybenzene (15.8 mg, 0.106 mmol, 1.00 equiv.), pinacolborane (15.4 µL, 0.106 mmol, 1.00 equiv.) and 1,3,5-trimethoxybenzene (2.00 mg, 0.0119 mmol, 0.112 equiv.) were added, and the solution was allowed to stir for 5 min at 25 °C. The reaction mixture was transferred to an NMR tube, and the tube was capped with a septum. The tube was removed from the glovebox, and 76 µL of a 1.4 M solution of distilled water in THF-d_8 (0.11 mmol, 1.0 equiv.) was added to the tube via microsyringe. The mixture was then shaken and the NMR spectrum recorded after 16 h.

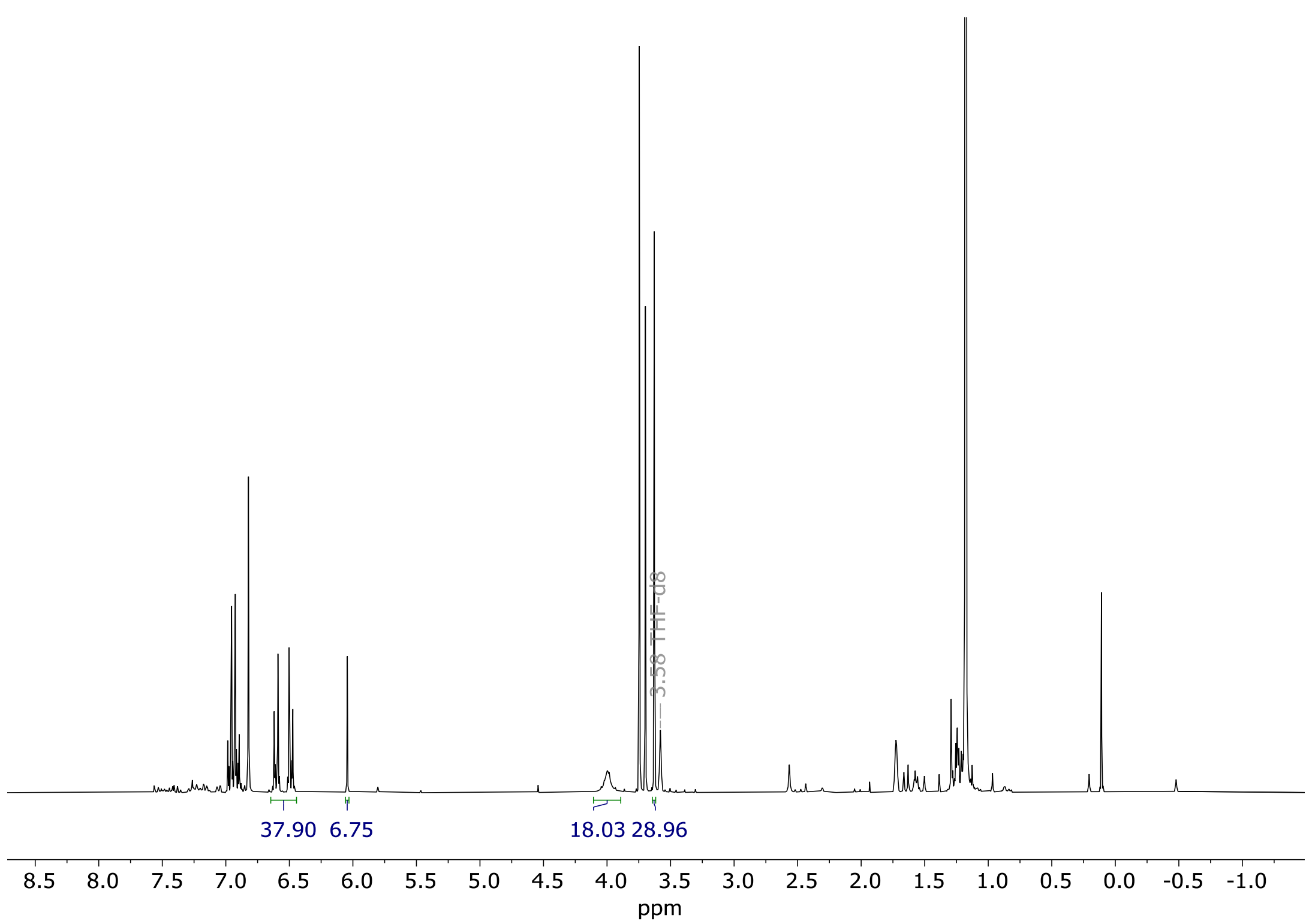

Figure S44. ^{1}H NMR spectrum displaying the catalytic reduction of 1-azido-4-methoxybenzene to 4-methoxyaniline in 47% yield after 16 h.

4.8. Catalytic reductive borylation of *p*-toluenesulfonyl azide

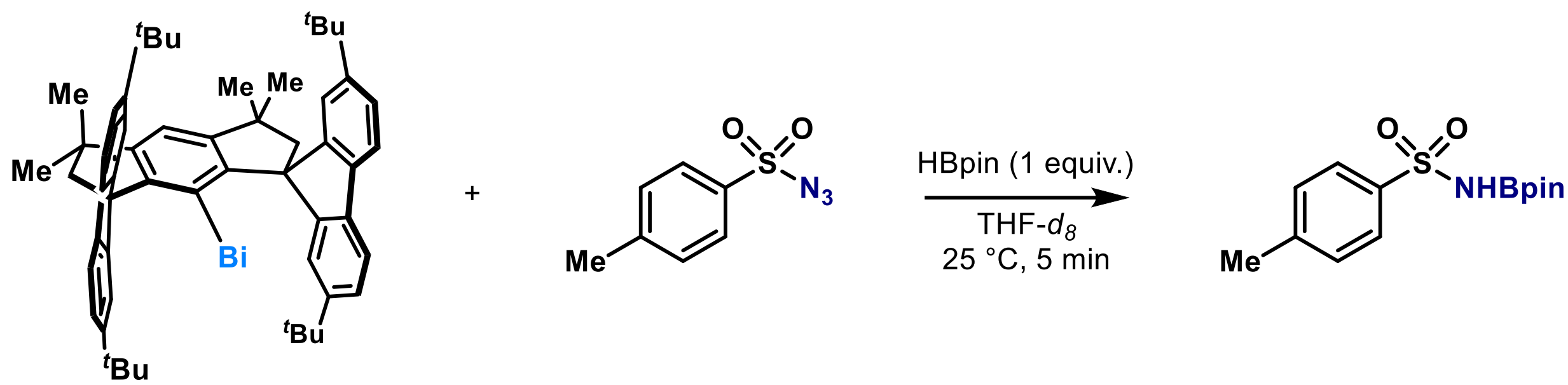

5 mol% **1 equiv.** **>99%**

Procedure: In an argon-filled glove box, **1** (5.0 mg, 0.0053 mmol, 5.0 mol%) was weighed in an oven-dried 12 mL vial, followed by the addition of 0.5 mL of THF-d_8. *p*-Toluenesulfonyl azide (21.0 mg, 0.106 mmol, 1.00 equiv.), pinacolborane (15.4 µL, 0.106 mmol, 1.00 equiv.) and 1,3,5-trimethoxybenzene (13.3 mg, 0.0791 mmol, 0.750 equiv.) were added, and the solution was allowed to stir for 5 min at 25 °C. The reaction mixture was transferred to an NMR tube, and the NMR spectrum recorded.

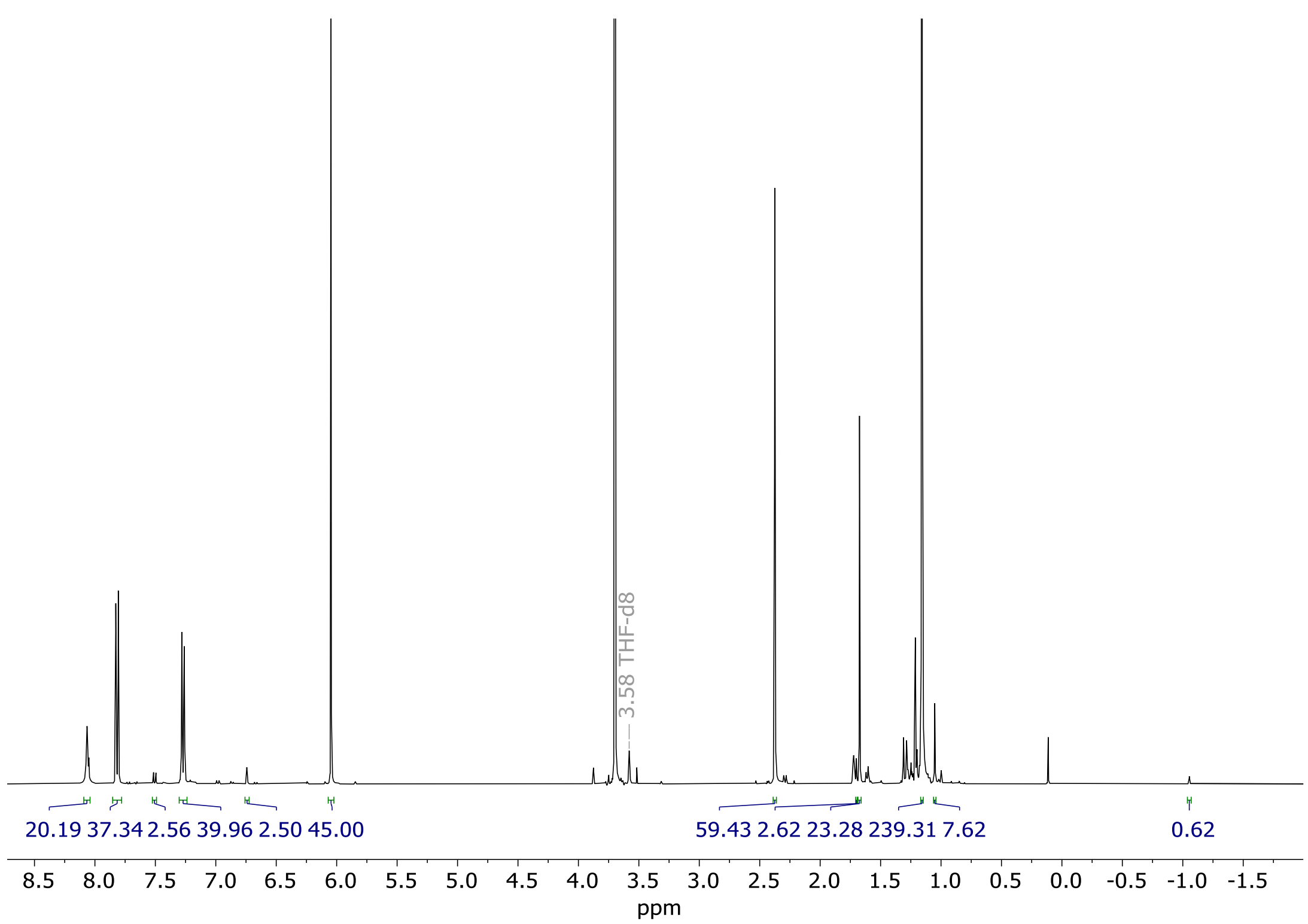

Figure S45. ^{1}H NMR spectrum displaying the catalytic reduction of *p*-toluenesulfonyl azide to 4-methyl-N-(4,4,5,5-tetramethyl-1,3,2-dioxaborolan-2-yl)-benzenesulfonamide in quantitative yield (>99%), as well as partial recovery of Bi catalyst **1** (62%).

4.9. Unsuccessful substrates

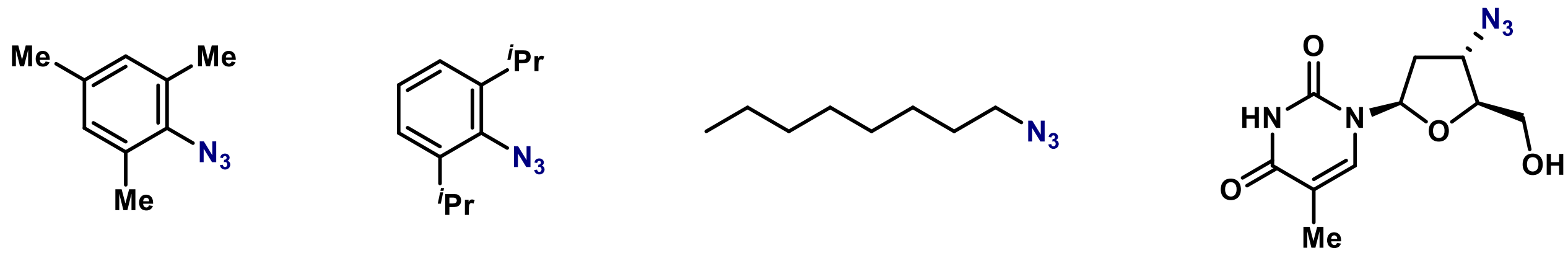

0% 0% 0% 0%

Figure S46. Overview of aryl and alkyl azides that demonstrated no reactivity with **1** to form the corresponding iminobismuthane or tetraazabismole species, as well as no yield of the corresponding amine when subjected to the standard reaction conditions of the catalytic reduction (5 mol% **1**, 1.0 equiv. HBpin, 1.0 equiv. H_2O, 0.5 mL THF-d_8 at 25 °C for 16 h).

5. X-ray absorption spectroscopy (XAS)

Bi L_3-edge XAS spectra were collected with an *easyXES100-extended* laboratory spectrometer (easyXAFS, LLC, Seattle, USA) using a palladium cathode source operating at 25 kV accelerating potential.[12,13] A circular aperture of 1 mm diameter was placed in front of the source to improve resolution. A helium flight path encompassing the Rowland circle area was used to reduce the X-ray attenuation in air. A circularly bent Si(880) crystal with 1 m radius was used as monochromator. The samples were diluted to 70, 52, 10 and 12 % mass fraction in boron nitride for [Bi(N_4(1,1'-Ph_2)$_2$)(^{t}BuM^{s}Fluind)], [Bi(^{t}BuM^{s}Fluind)Br_2], metallic Bi and Bi_2O_3, respectively. The resulting powder was ground and packed into a plastic cell (5 mm diameter, 1 mm thickness) and sealed with Kapton tape; in the case of [Bi(N_4(1,1'-Ph_2)$_2$)(^{t}BuM^{s}Fluind)], the pellet was prepared in a in a N_2-filled glovebox and kept in strictly anaerobic condition, since a colour change from blue to yellow was observed on this sample when XAS was measured on a sample prepared in air. The transmitted photons in the 13380 – 13550 eV range were detected by a Vortex detector. The samples were measured under constant stream of dry N_2 flow to hinder permeation of air into the cells. At least ten scans were acquired consecutively with 1 eV step across the Bi L_3-edge region and spaced data points before and after for normalization. Each scan was inspected individually for radiation damage and oxidation and then averaged together. The incident flux (I_0) was measured in the same way without any sample. The energy axis of all spectra was calibrated versus the first inflection point of metallic Bi spectrum at 13419 eV measured in the same conditions. The final spectra were obtained with the $\ln(I_0/I_1)$ relationship and normalized. A five-point smoothing was applied to the spectrum of metallic Bi to improve clarity, while the spectral features were neither suppressed nor introduced (see Figure S47).

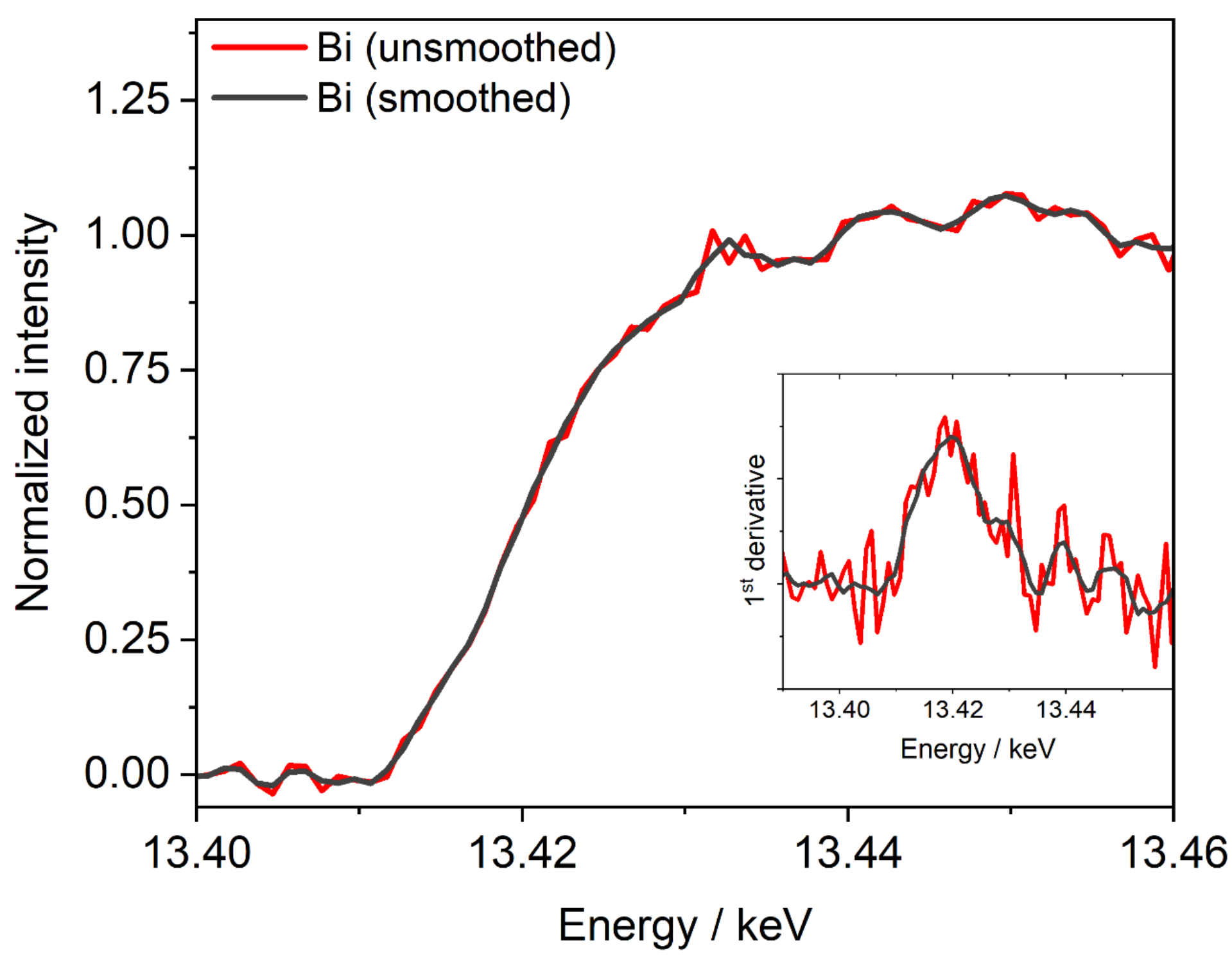

Figure S47. Average of 10 Bi L_3-edge XAS scans of metallic Bi in red overlaid with the respective smoothed spectra in gray, as produced by five-point Savitzky-Golay smoothing algorithm.

6. Molecular modeling

6.1 Computational details

All calculations were performed with the ORCA electronic structure package version 6.1.[14,15] Geometry optimizations were carried out using restricted density functional calculations with the B3LYP exchange-correlation functional,[16-19] the DLU-X2C approximation for relativistic effects,[20-28] the D4 dispersion correction,[29-30] and a conductor-like polarizable continuum model (CPCM, CH_2Cl_2) of solvation were used.[31,32] x2c-TZVPall basis sets were used for all elements and the RIJCOSX approximation was used with x2c/J auxiliary basis sets.[33-36] Frequency calculations were carried out with the same setup on the identified local minima, but an expanded x2c-QZVPall basis set was used on the Bi atom. IR spectra were subsequently simulated using the orca_mapspc tool included in ORCA, applying a Gaussian broadening of 25 cm^{-1}. Crystal structures were used as an initial guess for geometry optimizations of all compounds, and convergence to a minimum energy geometry was confirmed by the absence of any imaginary frequency. UV-Vis spectra were calculated by time-dependent DFT within Tamm-Dancoff approximation using the CAM-B3LYP functional on B3LYP-optimized geometries and including spin-orbit coupling effects.[37] The oscillator strengths for the first 500 roots were computed based on the sum of the full semiclassical field-matter interaction operator as implemented in ORCA, including electric dipole, magnetic dipole and electric quadrupole contributions. The transitions were assigned based on the inspection of the natural transition orbitals. Since TD-DFT spectra achieved a good description of the experimental features, and attempts at complete active space (CAS) calculations based on previous reports on similar compounds were found to be extremely difficult to converge even after active space expansion to higher level shells, the multiconfigurational character of the compounds was deemed to be negligible.[38-40]

Valence occupancy for **2** and **3** was explored using the Pipek-Mezey localization scheme,[41] as natively implemented in ORCA and used to visualize complex molecular orbital ensembles in terms of easily recognisable σ- and π-type orbitals. Nucleus-independent chemical shieldings (NICS) were computed at the center of mass of benzene, phenylpentazole and **3** as natively implemented in ORCA.[42-43]

6.2 IR spectroscopy

Compound **2** exhibits a complex absorption at around 3000 cm^{-1}, reflecting the antisymmetric and symmetric C-H stretching vibrations of both aliphatic and aromatic moieties of the molecule. Conversely, features at lower wavenumbers (<1650 cm^{-1}) instead correspond to either stretching modes of heavier atoms or deformations that involve larger groups of atoms. A comparison of experimental and calculated IR spectra for **2** is shown below (Figure S48).

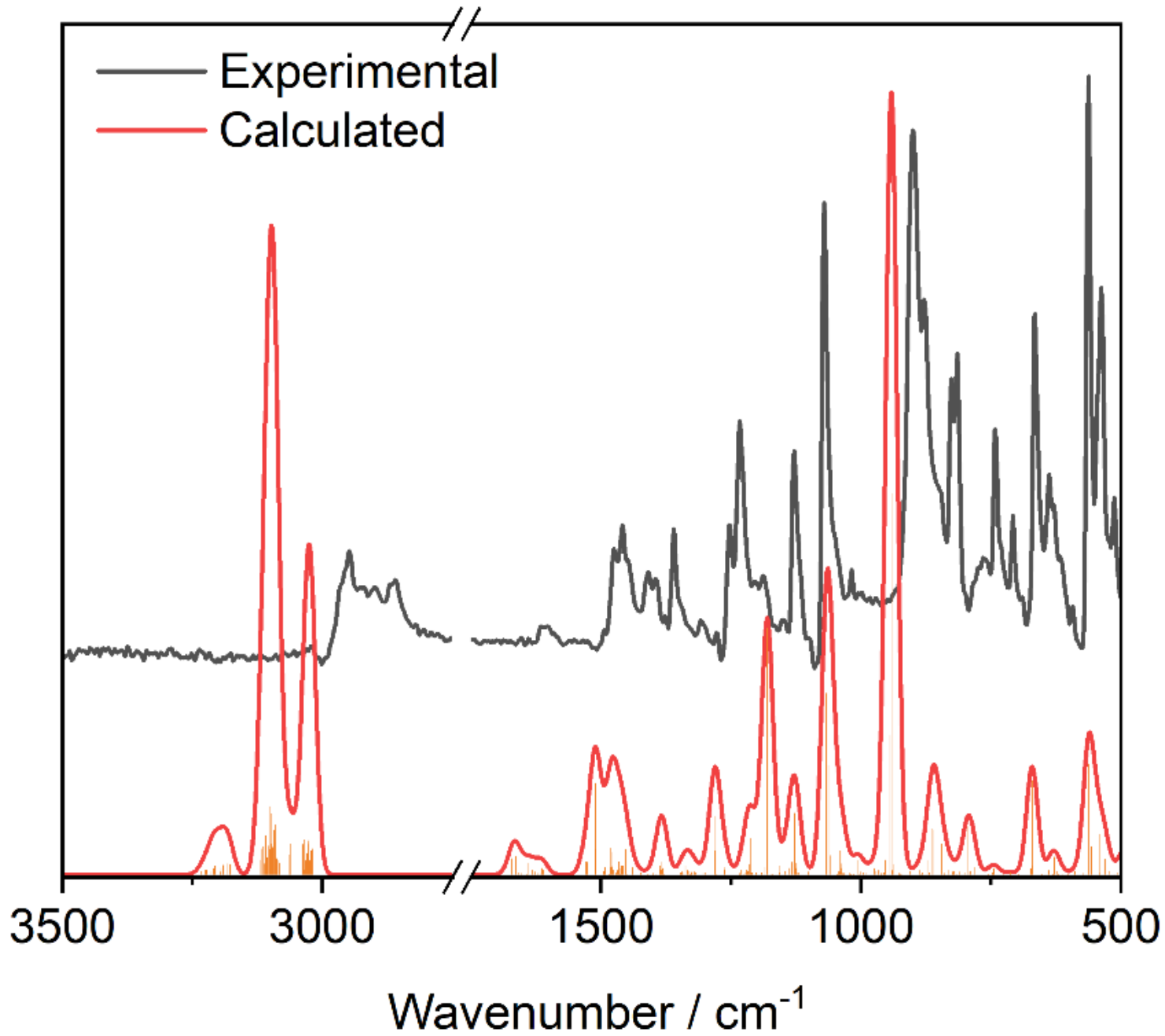

Figure S48. Comparison of experimental (black) and calculated (red) IR spectra for **2**. Gaussian broadening of 25 cm^{-1} was applied to the calculated vibrational modes for ease of comparison with the experimental data. Individual normal modes from the calculation are shown as orange sticks. No shift was applied to the calculated spectra. The spectra have been vertically shifted for better visualization.

The calculated spectrum is in good agreement with the experimental one, allowing for assignment of specific (intense) features. The C-H stretching features around 3000 cm^{-1} are significantly shifted compared to the experiment, as a consequence of substantial anharmonicity effects of these modes that are not captured in regular frequency calculations. Nonetheless, these normal modes are well-known and it is beyond the scope of our method to accurately reproduce them with calculations. In the following, the vibrations will be described according to the scale of the experimental spectrum (and not the calculated one) for ease of interpretation.

The weak absorption manifold around 1600 cm^{-1} corresponds to C-C stretching in the aromatic moieties consisting of 6-membered rings, akin to the e_{2g} vibrational modes of benzene. All absorptions at lower wavenumbers correspond to expected deformation modes of the aromatic structure of the molecule according to functional groups. Below 850 cm^{-1}, vibrational modes reflect significantly delocalized modes that involve significant deformation of the entire molecule. Some notable diagnostic peaks include:

- 1229 cm^{-1}: antisymmetrical stretching of the -SO_2- moiety;
- 1070 cm-1: symmetrical stretching of the -SO_2- moiety (with some deformation of the p-toluensulfonyl group);
- 900 cm^{-1}: stretching mode of the -N-S- bond;
- 667 cm^{-1}: deformation mode of the p-toluensulfonyl group;
- 635 cm^{-1}: stretching mode of the -Bi-N- bond;
- 561 cm^{-1}: deformation mode of the p-toluensulfonyl group;
- 539 cm^{-1}: deformation mode of the p-toluensulfonyl group.

Compound **3** exhibits a complex absorption at around 3000 cm^{-1}, reflecting the antisymmetric and symmetric C-H stretching vibrations of both aliphatic and aromatic moieties of the molecule. Conversely, features at lower wavenumbers (<1650 cm^{-1}) instead correspond to either stretching modes of heavier atoms or deformations that involve larger groups of atoms. A comparison of experimental and calculated IR spectra for **3** is shown below (Figure S49).

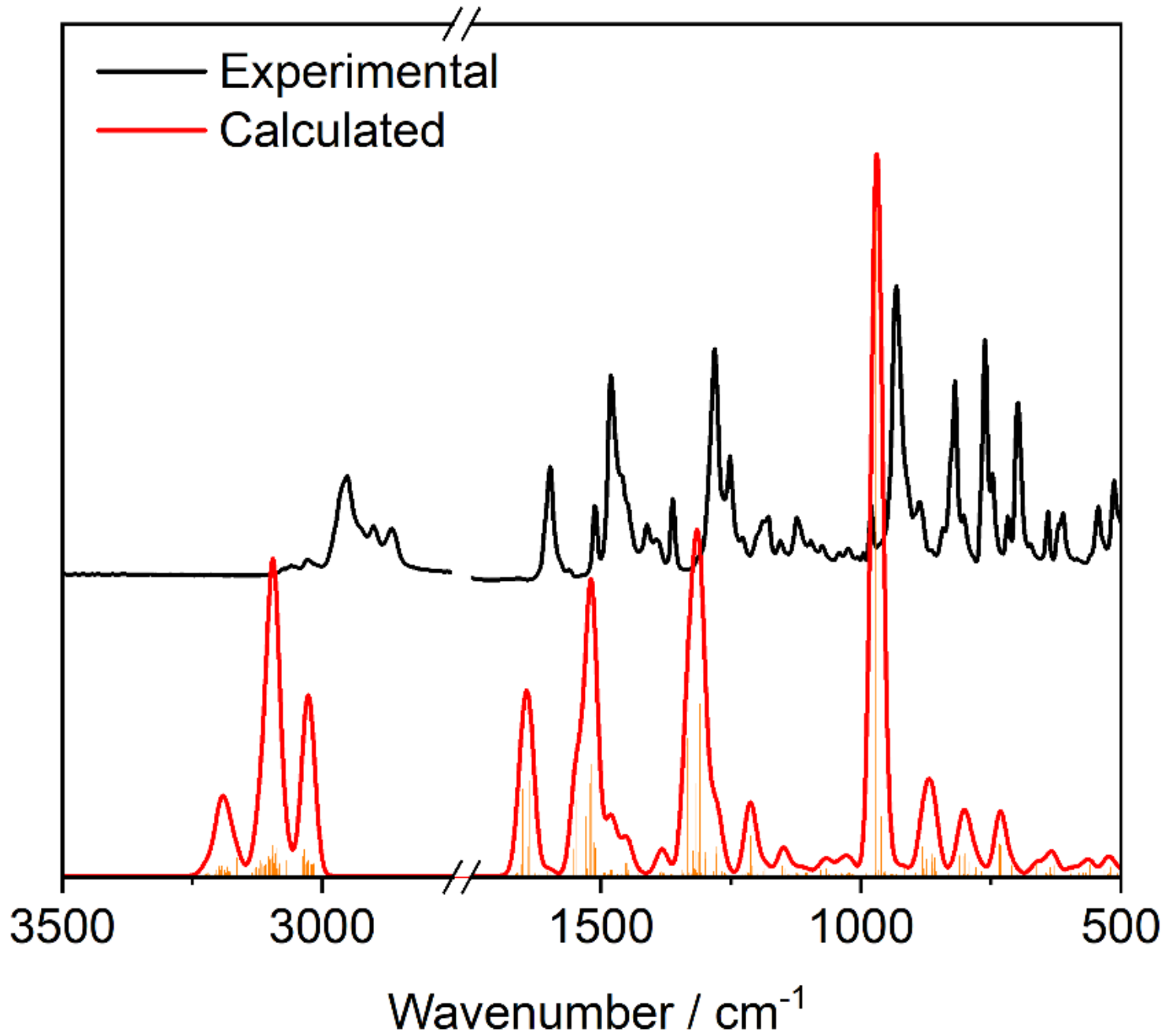

Figure S49. Comparison of experimental (black) and calculated (red) IR spectra for **3**. Gaussian broadening of 25 cm^{-1} was applied to the calculated vibrational modes for ease of comparison with the experimental data. Individual normal modes from the calculation are shown as orange sticks. No shift was applied to the calculated spectra. The spectra have been vertically shifted for better visualization.

The calculated spectrum is in excellent agreement with the experimental one, allowing for assignment of specific (intense) features. The C-H stretching features around 3000 cm^{-1} are significantly shifted compared to the experiment, as a consequence of substantial anharmonicity effects of these modes that are not captured in regular frequency calculations. Nonetheless, these normal modes are well-known and it is beyond the scope of our method to accurately reproduce them with calculations. In the following, the vibrations will be described according to the scale of the experimental spectrum (and not the calculated one) for ease of interpretation.

The sharp absorption feature at 1600 cm^{-1} correspond to C-C stretching in the aromatic moieties consisting of 6-membered rings, akin to the e_{2g} vibrational modes of benzene. Compared to **2**, this feature is less broad and more intense due to the more similar structure of 6-membered aromatic groups for **3** (*e.g.*, the absence of the p-toluensulfonyl group). All absorptions at lower wavenumber correspond to expected deformation modes of the aromatic structure of the molecule according to functional groups. Below 850 cm^{-1}, vibrational modes reflect significantly delocalized modes that involve significant deformation of the entire molecule. Some notable diagnostic peaks include:

- 1510 cm^{-1}: C-C stretching in the 6-membered rings of the $Bi(N_4\{1,1'\text{-}Ph_2\}_2)$ unit, akin to the e_{1u} vibrational modes of benzene (antisymmetric motion in two adjacent 6-membered rings);
- 1478 cm^{-1}: C-C stretching in the 6-membered rings of the $Bi(N_4\{1,1'\text{-}Ph_2\}_2)$ unit, akin to the e_{1u} vibrational modes of benzene (symmetric motion in two adjacent 6-membered rings);
- 1281 cm^{-1}: C-N stretching of the the $Bi(N_4\{1,1'\text{-}Ph_2\}_2)$ unit (involving significant contribution from the whole moiety);
- 980 cm^{-1}: N_1-N_2 and N_3-N_4 symmetrical stretchings of the tetraazabismole ring;
- 930 cm^{-1}: N_1-N_2 and N_3-N_4 antisymmetrical stretchings of the tetraazabismole ring;
- 700 cm-1: C-C bending in the terminal 6-membered rings of the $Bi(N_4\{1,1'\text{-}Ph_2\}_2)$ unit, akin to the a_{2u} vibrational modes of benzene.

6.3 UV-Vis spectroscopy and natural transition orbitals (NTOs)

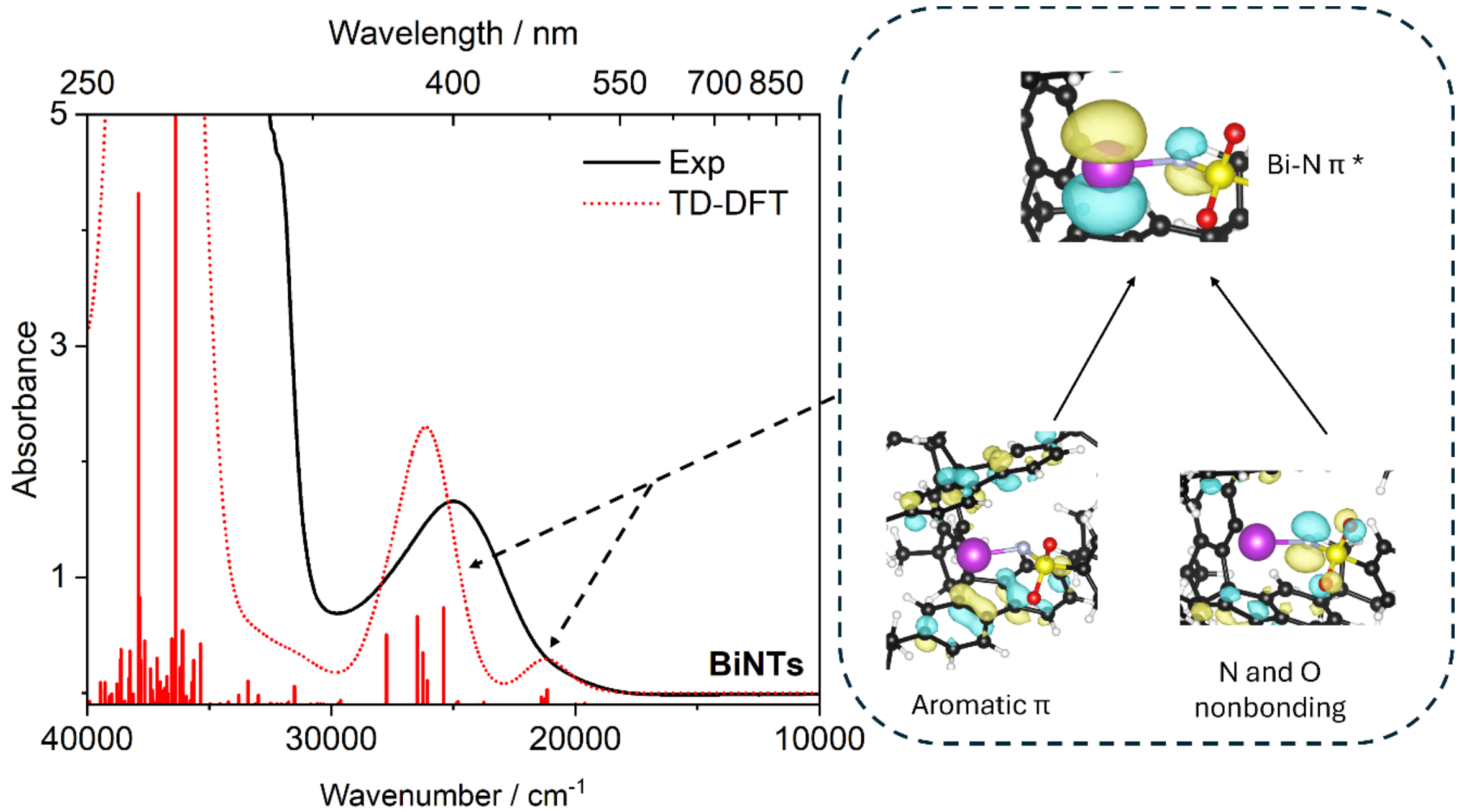

Figure S50. Comparison of experimental (black) and simulated (red) UV-Vis spectra for **2**. Vertical sticks indicate individual transitions before gaussian broadening. Most intense transitions in the experimental spectrum are assigned according to NTOs associated to the main transition under each peak (reported on the right).

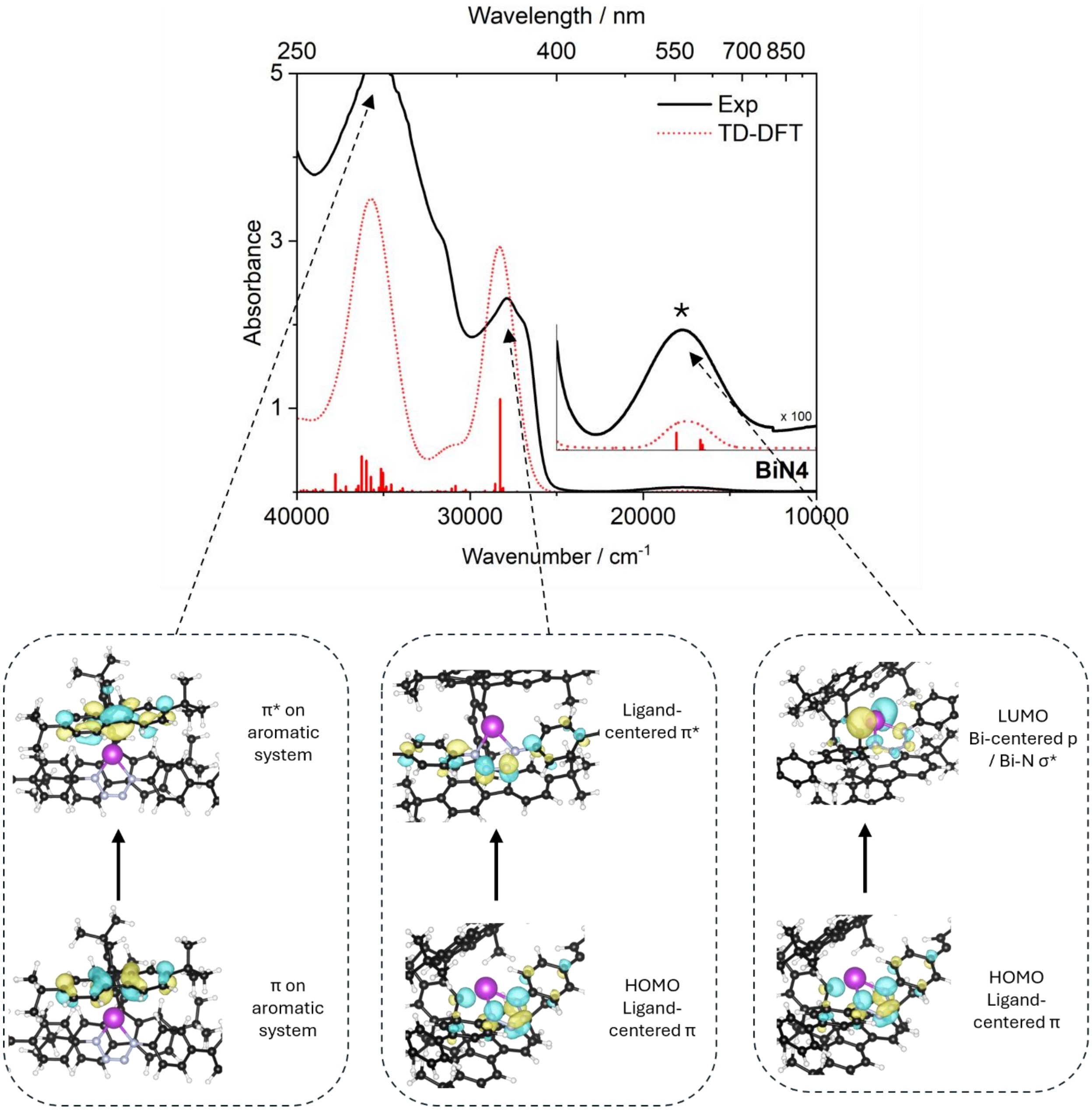

Figure S51. Comparison of experimental (black) and simulated (red) UV-Vis spectra for **3**. Vertical sticks indicate individual transitions before gaussian broadening. Most intense transitions in the experimental spectrum are assigned according to NTOs associated to the main transition under each peak (reported below). The features under the low-energy peak marked by an asterisk (HOMO – LUMO transition) are split due to spin-orbit coupling.

6.4 ETS-NOCV Analysis

The Extended Transition State–Natural Orbitals for Chemical Valence (ETS-NOCV) scheme has been performed using its native implementation in the ORCA 6.1 code,[44-47] as described in the original publication by Cárdenas Sabando et al.,[48] using the same computational setup employed for the UV-Vis calculations. This technique was used to quantitatively assess the energetic contributions to chemical bonding in the Bi-C and Bi-N bonds in **2**, which are reported in Table S5.

	**Bi - carbene**	**Bi - imine**
ΔE_{int}	-107.74	-123.84
ΔE_{orb}	-125.29	-203.84
ΔE_{Pauli}	302.30	356.46
ΔE_{elstat}	-127.82	-130.88
ΔE^{0}_{XC}	-109.60	-111.02
ΔE_{disp}	-43.00	-24.51
ΔE_{CPCM}	-4.28	-10.03

Table S5. ETS analysis for the Bi-N and Bi-C bonds (corresponding to the imine and carbene moieties) in **2**. All energies are reported in kcal/mol.

In both cases, the interaction between Bi and the ligand is remarkably strong, indicating the thermodynamically favored formation of a chemical bond. Both ligands present a strong Pauli repulsion term, with a more marked repulsion in the case of the imine moiety. Adding up the Pauli (repulsive) and electrostatic (attractive) contributions to energy, yielding the total steric contribution to bonding,[49,50] the resulting interaction is still repulsive in both cases; for the Bi-imine bond, the interaction is further sterically destabilized by ca. 50 kcal/mol compared to the Bi-carbene, likely due to the lower Bi-N bond distance. This result highlights how, even though the electrostatic attraction between Bi and its ligands contributes significantly to the stabilization of the complex, it is not the dominant attractive force responsible for chemical bonding. The major term contributing to the stabilization of these bonds is ΔE_{orb} (responsible for covalent bonding via polarization and charge transfer), which is largely stabilizing in both cases and especially so for the Bi-imine bond. The most significant deformation densities plots contributing to the ΔE_{orb} term are shown below (Figure S52).

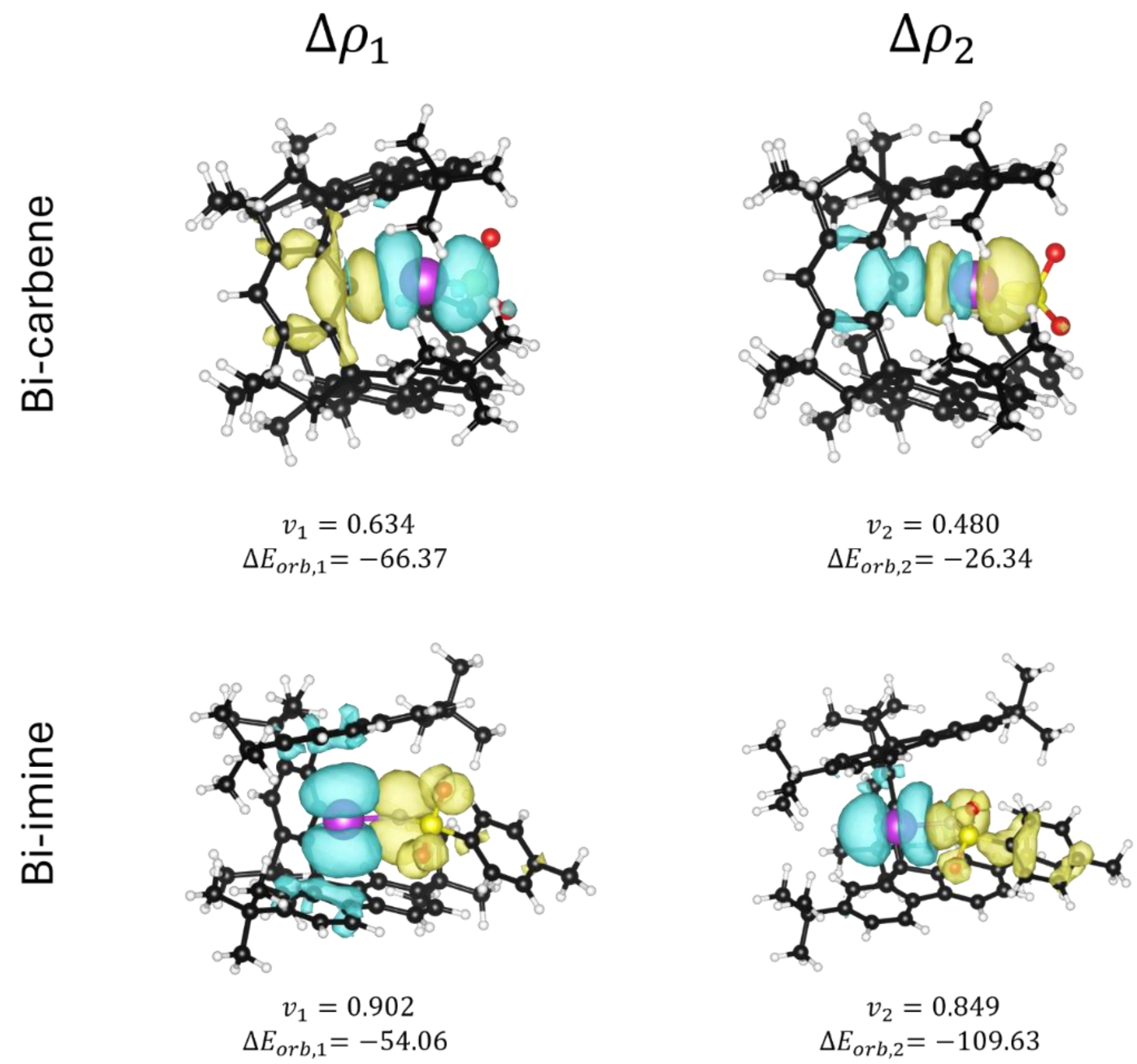

Figure S52. First two NOCV deformation densities $\Delta\rho1$ (left panels) and $\Delta\rho2$ (right panels) for the Bi-carbene and Bi-imine fragments interactions. The corresponding NOCV eigenvalues (v1, v2) and orbital interaction energy contributions (ΔE_{orb},1, ΔE_{orb},2) are shown below each panel. The energy contributions are expressed in kcal/mol. All deformation densities are visualized at an isosurface level of 1×10^{-4} e/Bohr^3. Cyan and yellow surfaces indicate regions of charge depletion and accumulation, respectively.

The deformation density plots for the Bi-carbene interaction indicate that this bond is characterized by essentially a σ interaction (charge transfer from Bi and back-donation from C); conversely, the Bi-imine bond shows strong interactions (in terms of relative energetic contributions) from both σ and π contributions. This difference further emphasizes the difference in the nature of the two bonds, strengthening the assignment of a Bi-C single and Bi-N double bond, respectively, as already suggested by both experimental (from crystallography) and modeling (from localized orbitals) arguments.

6.5 Cartesian coordinates for optimized structures

Compound **2**:

Bi	-0.99642946573707	-0.42764873712893	0.83821829450910
S	1.17060593546238	0.50890300071168	2.85244006501889
O	0.70736302157812	1.75107456574383	3.48425520247399
O	0.79358357279603	-0.69310261166418	3.61116279328917
N	0.82445806028246	0.43242200739925	1.31978463915616
C	-0.66564126460085	-0.24291639237375	-1.38623969763176
C	-0.20483971268645	-1.33889068124239	-2.10531756875593
C	-0.82011260422590	-0.32500713212919	-4.20431898218054
H	-0.92300023057611	-0.37489251238493	-5.28215587915522
C	-1.24799247877580	0.79635898306465	-3.50339661499115
C	-1.15686010226765	0.83301016210770	-2.11478348638352
C	0.45941904783640	-2.57606790519552	-1.53573159257381
C	0.48685479432894	-3.54707892276602	-2.75558395259835
H	-0.28249901115584	-4.30657316291950	-2.61793324447137
H	1.44265817370466	-4.06534282796152	-2.82261709541298
C	0.17972711956100	-2.72527317035079	-4.04534665144774
C	1.41806338968697	-2.56099991870412	-4.93849565881618
H	1.75055776435318	-3.53464000771842	-5.30657706320694
H	2.24699405073351	-2.10312812228107	-4.39951641627929
H	1.18875772125534	-1.93194919823941	-5.80154428068200
C	-0.93533279905674	-3.40074138623908	-4.85676605210786
H	-0.61900995749924	-4.39506342103977	-5.18199947190228
H	-1.18211599051423	-2.81729225839399	-5.74651850734214
H	-1.84046331768857	-3.50827190607719	-4.25551604279485
C	-1.66230783962541	2.14872728075150	-1.55420746758617
C	-2.34007611801791	2.81535493695702	-2.78953949329688
H	-2.08512913033536	3.87242954344212	-2.85316348725066
H	-3.42180421325307	2.75137091163058	-2.67410170009739
C	-1.90847270309463	2.03689616174714	-4.06983398070239
C	-0.93240552992492	2.84259746798124	-4.93983515662809
H	-0.61111994774174	2.25256401053462	-5.80106147929894
H	-0.04274188254702	3.13571933056121	-4.38318204456251
H	-1.41540636611183	3.75011060383236	-5.31008522212397
C	-3.13873059860466	1.66772843984860	-4.91096272371938
H	-3.65553064411695	2.57174840539722	-5.24296788937564
H	-3.84009550879138	1.06607809851040	-4.32926803047858
H	-2.85234973897176	1.09890585443813	-5.79819419948703
C	-0.24422731578385	-3.13858503680718	-0.31309415811745
C	-1.54093160393285	-3.63236897373666	-0.21442187706292
H	-2.18202682834978	-3.60549390097865	-1.08396702507004
C	-1.99835117383260	-4.16497197880426	0.99132247702196
C	-1.12271884099969	-4.16172245302133	2.09143206028892
H	-1.46054466859885	-4.56131021622408	3.03802959954725
C	0.16801010854700	-3.66819387565636	2.01161997804856
H	0.81358175060834	-3.68136493666459	2.87949671600042
C	0.62159153227143	-3.16400370937117	0.79372217023689
C	1.91598239159262	-2.63605011526420	0.38609214749680

C	3.08601058227264	-2.43592829472281	1.09789710907217
H	3.15835922029024	-2.71661998990141	2.14043996040749
C	4.17016535476776	-1.84064062712033	0.46055455081734
H	5.07207892036179	-1.67808809588412	1.03104160832332
C	4.10141127187837	-1.42845096525489	-0.87221122342331
C	2.90550600657412	-1.64305855648909	-1.57305430047786
H	2.82028197654128	-1.31257851385922	-2.59818756173452
C	1.83593305982504	-2.25611904536736	-0.95984549365228
C	-3.39453459860120	-4.76669318779334	1.14983622584082
C	-0.28921316339730	-1.39435956187223	-3.49342680145656
C	5.26262815067454	-0.73269025705446	-1.58494464968208
C	-2.58184722152993	1.94197582669947	-0.36728107174684
C	-3.77870303454942	1.25053072061606	-0.32485604998466
H	-4.15667257547254	0.79168940581747	-1.22886405964379
C	-4.49428923232232	1.14506477349170	0.87368676557587
C	-3.95293793847223	1.74478561486857	2.01768000473213
H	-4.47191426874434	1.66598466928503	2.96134610433806
C	-2.75295715753739	2.44847291338848	1.98887201358474
H	-2.36662914306076	2.90065508893174	2.89331647258389
C	-2.06462121277949	2.56269257428857	0.78461572307205
C	-0.82814795018139	3.24468334371889	0.42173534000765
C	0.04190253386316	4.02144051916428	1.17451642683999
H	-0.15697642424721	4.22527728732517	2.21794342128658
C	1.18783193019573	4.51677517470318	0.57190995805760
H	1.85891349586313	5.12701231619569	1.16171411395537
C	1.50026171814044	4.24256866291865	-0.76805049511893
C	0.60928794991362	3.45796746905610	-1.50563769093516
H	0.82384980511505	3.21779423045060	-2.53500083112154
C	-0.55261991542408	2.98438713717244	-0.92275708805167
C	2.76967379792958	4.83536010633569	-1.38556687376709
C	2.63513674368057	6.37086425218744	-1.41493795526976
H	1.77228359032164	6.67346542815131	-2.01266989798119
H	3.53002730318682	6.81844776223898	-1.85463171363813
H	2.51179944110474	6.77839063891644	-0.41026146541435
C	3.00362471208074	4.34131940312338	-2.81861997212159
H	3.09860228360723	3.25443366083246	-2.85980304361553
H	3.92932828524626	4.77294842183864	-3.20425232722186
H	2.19465171195992	4.63940086878384	-3.48848340715836
C	3.99775296637087	4.45275747172239	-0.53914062304214
H	3.91907224150368	4.82102401875158	0.48420595899902
H	4.90105569448347	4.88050416268022	-0.98043783510611
H	4.11935774630270	3.36989639698786	-0.50127850720366
C	2.95118840287538	0.59819865989604	2.87277823560458
C	3.60578154919486	1.51879608979563	2.06169184018330
H	3.03995700697954	2.15036136700674	1.39091384507143
C	4.98727668726432	1.61733555130299	2.11366030476875
H	5.49277289878294	2.33567460281147	1.47948968250515
C	5.73906182435057	0.80558246908296	2.96743694562773
C	5.06093571077949	-0.10695595499997	3.77470340877682
H	5.62209122466134	-0.74475906065895	4.44781815064499
C	3.67630185617470	-0.21389122196766	3.73243377589463

H	3.16140093738358	-0.92818473518121	4.35942116076833
C	7.23945007975301	0.89282276920628	2.99306415824728
H	7.67937517803313	0.18606087882274	2.28284608261651
H	7.63306054076479	0.64959677113324	3.98098752525603
H	7.58330031697621	1.89026893469399	2.71607746921344
H	-3.79405173387609	-5.14470056727198	-0.96560705232239
C	5.63570822204198	-1.52013095193437	-2.85583014491025
C	6.51128770135520	-0.62527302973826	-0.70239086047612
C	4.81907668055679	0.69010891920315	-1.97688304655389
H	3.97256217508665	0.67172988421703	-2.66495668940486
H	5.63950840794878	1.22157380553385	-2.46517869017962
C	-6.36747922639142	0.18834438991593	2.30405821334359
H	-6.56798316338971	1.13246703537370	2.81372648968880
C	-3.25320672585492	-6.27057467283671	1.46001330127869
H	-2.74185621904046	-6.78737332067898	0.64480625260661
H	-4.24140482623897	-6.71984975046539	1.58396848407170
H	-2.68797815798813	-6.43927439115063	2.37788858702537
C	-4.23895420125053	-4.61502228715261	-0.12085841376380
H	-4.36530464682711	-3.56873665110753	-0.40169939517626
H	-5.23017547967596	-5.03792778115299	0.05184406563910
C	-4.12723038313299	-4.07701605947583	2.31687306328257
H	-3.61727906799409	-4.24097116523473	3.26668347790344
H	-5.13998346407569	-4.47591347901451	2.40798162023659
H	-4.19745416932939	-3.00091903989040	2.15354744958735
H	4.52271712583664	1.25623595861941	-1.09311427060803
C	-5.83207810186413	0.40561569957796	0.88361236562848
C	-5.66789864819122	-0.97133463198335	0.21579104613579
H	-4.88694562946263	-1.54701869887828	0.71207557712808
H	-5.40583673297561	-0.88797552819535	-0.83927276618925
H	-6.60236258701362	-1.53223276195549	0.28351349298714
C	-6.85851577947536	1.23816023654344	0.09010276217162
H	-6.52981719868935	1.39583684637335	-0.93863736599938
H	-7.00712041722449	2.21592683212171	0.55382365466353
H	-7.82112451180078	0.72149162834305	0.06432853337412
H	-5.66772636007944	-0.38880416598159	2.91239967730807
H	5.94980691673588	-2.53572272281683	-2.60391810556885
H	6.87391104934560	-1.60832919933419	-0.39450572047226
H	6.46190645822706	-1.02589945685860	-3.37281411218577
H	6.32199835789925	-0.03436150405677	0.19354362020307
H	7.30938491942541	-0.13528056214161	-1.26365211203346
H	4.79745499369328	-1.58657765024228	-3.55042375956707
H	-7.30644298762591	-0.36592510908086	2.25452594693930

Compound **3**:

Bi	2.63271002322113	17.08116591309696	21.06383512076501
N	1.30608382794584	18.40821211373277	19.89440092441340
N	0.16084049561432	17.83519005516839	19.42695423328800
N	-0.11484735837573	16.67718037193147	19.80204519891563
N	0.71786009394452	16.06631834454426	20.68642395356163
C	2.51318180557636	17.53396442807021	23.32305527876575
C	3.75970865180845	17.24035882035229	23.90877372993461
C	5.10992025055008	17.00436930074564	23.21946822456879
C	6.11673637182894	16.90469206787910	24.42257544856448
H	6.78856877537393	16.05702250626660	24.29716938888411
H	6.73062013950140	17.80357198176553	24.43844748997548
C	5.30645162618600	16.81105217385576	25.73611177781060
C	3.91758695194589	17.20413586925420	25.29299790068405
C	2.85844287916634	17.51161621178833	26.12687999867207
H	2.98145111724617	17.49867130911089	27.20304963184154
C	1.64869196710187	17.87858656925340	25.56451881587536
C	0.44647777174297	18.34721302330883	26.35007559707386
C	-0.50391559684141	18.83481976819172	25.23561438779621
H	-0.49718027717391	19.92301756451602	25.21287701023135
H	-1.53351905890469	18.52104542142388	25.40078513827907
C	0.02898770457202	18.30361690428715	23.85061127919511
C	1.45572283366990	17.87398561048742	24.17747637778318
C	5.29282049083003	15.38071889816666	26.30053668397837
H	6.30458195929332	15.07057087139236	26.57307710616011
H	4.90251203398780	14.66997405524044	25.57152049057054
H	4.66590242020072	15.32630825416246	27.19339171020655
C	5.87018524897322	17.76839841791967	26.79305274848426
H	5.85257392697908	18.79877654770638	26.43208347693110
H	6.90464433111187	17.50564891427418	27.02772992700384
H	5.29414170058817	17.71863177266409	27.71944587173926
C	-0.15566509930328	17.21238139925502	27.19284459575885
H	-1.03606997134625	17.57198164342593	27.73105307208454
H	0.56830203629260	16.85436637108880	27.92850019548541
H	-0.45848902762462	16.36601022032384	26.57846771906995
C	0.81964889030988	19.50985835354941	27.28294932488601
H	1.28725990180858	20.32126512256556	26.72160608063374
H	1.51426534280422	19.18494576629909	28.06053041604546
H	-0.07479267978083	19.90281022170505	27.77299541983056
C	5.24656505087702	15.79413475560699	22.29949950818522
C	4.94617227281919	14.46741783670305	22.55948165345768
H	4.49360460390668	14.20182225240568	23.50284887668803
C	5.23629451722432	13.48065231015508	21.61243226405962
C	5.80391088415863	13.88019329771390	20.39389532231508
H	6.02630590390460	13.13538134059164	19.64121027094920
C	6.10181546541202	15.20694992969577	20.11839187528095
H	6.54132197314111	15.48054820778674	19.16685966848419
C	5.83244466099828	16.16898236183645	21.08235965404642
C	6.01165207946752	17.61591873205499	21.07164377443796
C	6.53036304106975	18.47873646212956	20.11713662095863

H	6.88698530129899	18.10610853932547	19.16460214785563
C	6.60822041170810	19.83810460326421	20.40626501717096
H	7.02945267418710	20.49509736968661	19.65959498604528
C	6.16983886272600	20.36257959550875	21.62686939304876
C	5.61265860578777	19.47936855169220	22.56137795458035
H	5.26226887827004	19.84785492857443	23.51714612366334
C	5.54488001602498	18.12829175487572	22.28975028343058
C	4.97940886560955	11.99428534558321	21.86713060321387
C	6.30941961411656	11.22320662803285	21.75134617142812
H	6.14065017691866	10.16020564110215	21.94033727873696
H	7.03343969426411	11.58871269923715	22.48326803208524
H	6.75084167613579	11.32112092365470	20.75876660464118
C	3.98913370021149	11.45795769433266	20.81579138666347
H	3.04269525268150	11.99716443679747	20.86130964418657
H	3.78686570003782	10.39967842547967	20.99567997187146
H	4.39041844614535	11.55696983701557	19.80611809854708
C	4.41051161225551	11.73740095294404	23.26734521057182
H	4.23722672178840	10.66763013155321	23.39788488456148
H	3.46208201318583	12.25004781878548	23.42443661387753
H	5.10527750728906	12.06077700126017	24.04548420662418
C	6.32703965302022	21.83994411360824	21.99561406318063
C	6.92838001006024	22.67291485865880	20.85695629965117
H	7.00951723111010	23.71359630137846	21.17661252955565
H	6.30807018107981	22.65051542488581	19.95951205055695
H	7.92908335738781	22.32857506311540	20.58863057096254
C	4.95850254009014	22.43542263276830	22.36715768162361
H	5.07209120541081	23.48068018289873	22.66394304857957
H	4.50003600055395	21.90021389417713	23.19908763889514
H	4.27005757704288	22.39721956770781	21.52244259882530
C	7.26868154359563	21.94213470414407	23.21319835604510
H	6.86731260891014	21.40761956475505	24.07545551958070
H	7.40257095419099	22.98911203704497	23.49596559929155
H	8.24966887449711	21.52231237679535	22.97952812108623
C	-0.92032121497079	17.22996333463646	23.33079669992267
C	-1.19362642371340	15.98398085063376	23.86547315006942
H	-0.57899242572902	15.60551325636345	24.66736379787733
C	-2.28085498416665	15.23747076302332	23.40153879580015
C	-3.03633033619542	15.76345876438562	22.34460726312860
H	-3.88539591702225	15.20824141534120	21.96889638952999
C	-2.74376986668459	16.99426173526355	21.77256128198424
H	-3.34715412681044	17.37133799822153	20.95547076092466
C	-1.68679974553971	17.73846626875153	22.27846365833529
C	-1.21181196395930	19.07867988414878	21.95629543694386
C	-1.65666154311502	20.01791747315374	21.03293104681430
H	-2.45049223651948	19.77911206997135	20.33558688013342
C	-1.08636924407668	21.28143794107044	21.03137344278877
H	-1.45225658689598	22.01250751528104	20.32221464363366
C	-0.07726800741251	21.64555787969250	21.93650656199985
C	0.39517707412902	20.66906804960183	22.81621664854816
H	1.17094213073435	20.90373015122471	23.53172044212272
C	-0.17160880039514	19.40483054654810	22.82516737495471

C	-2.73060484871054	13.94151681544640	24.08406148403675
C	-4.00822580970775	14.24981185921667	24.89238462532101
H	-4.36370980275886	13.34727214973203	25.39688890090961
H	-4.80721673609738	14.61407070148653	24.24396610474938
H	-3.81284413689458	15.01101805681900	25.65129439406288
C	-1.67266030745550	13.39220927315345	25.05030905256525
H	-0.72803517397341	13.19547352105802	24.54253868686782
H	-2.02752662551311	12.45266567348037	25.47888987132681
H	-1.48194343114491	14.07885845690021	25.87678624571275
C	-3.04991464363469	12.85056147673033	23.04763249984031
H	-3.36070732087700	11.93519706018498	23.55695014881131
H	-2.17611222860284	12.62180257495476	22.44076411261131
H	-3.85932559270109	13.14643567311663	22.37945320503637
C	0.41088228984037	23.09712663166091	21.97583561130411
C	-0.79605161811044	24.02903473188276	22.20393426882298
H	-1.30622694740763	23.78421855620953	23.13861232004735
H	-1.52218007684766	23.95825625761424	21.39321091892998
H	-0.45860246027352	25.06700388929197	22.26171325332457
C	1.41139500214848	23.34002400039188	23.11193057744958
H	2.30263070040966	22.72298822550751	23.00618723611167
H	0.96742340483644	23.13480086463959	24.08849674061264
H	1.72772433835944	24.38512943191375	23.10021479549638
C	1.08574261093943	23.46260238136501	20.64297676557845
H	0.41214503184317	23.31680813599309	19.79766181914164
H	1.96760459048510	22.84682417473903	20.47519150593261
H	1.39611281513459	24.51061748260007	20.65239410296003
C	1.73829199190113	19.55902832782497	19.24927968743624
C	1.03361052497850	20.15384576048933	18.19076350874293
H	0.08902406034410	19.73467347422540	17.88076689515000
C	1.53827198917225	21.27159479925031	17.55138256236910
H	0.95403036986080	21.71855296280879	16.75661836533128
C	2.76085053532903	21.84618394305249	17.91753105109091
C	3.45474201349316	21.24423657425471	18.96986890590204
H	4.41200744727223	21.64008054890945	19.27536742178610
C	2.95361278153997	20.14037998906524	19.63344408295566
H	3.52303691341934	19.73221813574442	20.46033709673396
C	3.28143410959218	23.05443646721816	17.25134816271001
C	3.04551017185074	23.29434849120179	15.89166658279045
H	2.49161800549346	22.56904875903109	15.30912392717282
C	3.53175741310549	24.43790170800145	15.27090983807068
H	3.34077392639329	24.59677015309544	14.21650713740139
C	4.26963087783910	25.37058973849845	15.99365361055289
H	4.64983285763725	26.26133631951419	15.50944385053888
C	4.51275352767487	25.14618762552195	17.34532854183104
H	5.07830561784396	25.86799154227544	17.92215968849734
C	4.02276830564034	24.00465479515989	17.96629183905189
H	4.20034713304546	23.85924596624671	19.02368522926171
C	0.49485442144811	14.71028963325182	20.91738386563880
C	-0.23872972165751	13.90457084483944	20.03735461941902
H	-0.65286143104289	14.33772151994739	19.13886225272399
C	-0.41514682181798	12.55816908920797	20.30340962558603

H	-0.96841588392926	11.95930489382738	19.59109017597735
C	0.12389200070896	11.95479588789637	21.44542917409401
C	0.85755757756813	12.77095109051855	22.31119611009159
H	1.26344581689136	12.35410223625084	23.22204994997495
C	1.04356053698979	14.11680483859197	22.05838537230721
H	1.58662265688293	14.72044936166404	22.77451580401764
C	-0.08647256935223	10.52350522256303	21.73435788412972
C	-1.25030714699680	9.86605229118875	21.31463614775100
H	-2.01642566208469	10.41872353535454	20.78689999507374
C	-1.45195011026084	8.51960913136699	21.58828941693456
H	-2.36353449540915	8.03700320566365	21.25741716437522
C	-0.49629681428450	7.79497454526242	22.29387423825977
H	-0.65362189436095	6.74542298736174	22.50864457175594
C	0.66290034636790	8.43474498049509	22.72174878401918
H	1.41824578400491	7.88195299373578	23.26707115772777
C	0.86511963547235	9.78044632481928	22.44487267662826
H	1.78182397509558	10.25325910530168	22.76852357980397

7. References

[1] Y. Pang, N. Nöthling, M. Leutzsch, L. Kang, E. Bill, M. van Gastel, E. Reijerse, R. Goddard, L. Wagner, D. SantaLucia, S. DeBeer, F. Neese, J. Cornella, *Science* **2023**, *380*, 1043–1048.

[2] F. R. Bou-Hamdan, F. Lévesque, A. G. O'Brien, P. H. Seeberger, *Beilstein J. Org. Chem.* **2011**, *7*, 1124–1129.

[3] F. Sebest, L. Casarrubios, H. S. Rzepa, A. J. P. White, S. Díez-González, *Green Chemistry* **2018**, *20*, 4023–4035.

[4] M. Rajendiran, M. Papke, C. Müller, R. Murugavel, *Organometallics* **2025**, *44*, 502–519.

[5] D. Kratzert, I. Krossing, *J. Appl. Cryst.* **2018**, *51*, 928–934.

[6] D. Kratzert, J. J. Holstein, I. Krossing, *J. Appl. Cryst.* **2015**, *48*, 933–938.

[7] O. V. Dolomanov, L. J. Bourhis, R. J. Gildea, J. A. K. Howard, H. Puschmann, *J. Appl. Cryst.* **2009**, *42*, 339–341.

[8] S. Ito, F. J. White, E. Okunishi, Y. Aoyama, A. Yamano, H. Sato, J. D. Ferrara, M. Jasnowski, M. Meyer, *CrystEngComm* **2021**, *23*, 8622–8630.

[9] G. Sheldrick, *Acta Cryst.* **2015**, *71*, 3–8.

[10] F. L. Hirshfeld, *Acta Cryst.* **1976**, *32*, 239–244.

[11] A. Thorn, B. Dittrich, G. M. Sheldrick, *Acta Cryst.* **2012**, *68*, 448–451.

[12] G. T. Seidler, D. R. Mortensen, A. J. Remesnik, J. I. Pacold, N. A. Ball, N. Barry, M. Styczinski, O. R. Hoidn, *Rev. Sci. Instrum.* **2014**, *85*, 113906.

[13] E. P. Jahrman, W. M. Holden, A. S. Ditter, D. R. Mortensen, G. T. Seidler, T. T. Fister, S. A. Kozimor, L. F. J. Piper, J. Rana, N. C. Hyatt, M. C. Stennett, *Rev. Sci. Instrum.* **2019**, *90*, 024106.

[14] F. Neese, *WIREs Comput. Mol. Sci.* **2025**, *15*, e70019.

[15] F. Neese, *J. Comput. Chem.* **2023**, *44*, 381–396.

[16] C. Lee, W. Yang, R. G. Parr, *Phys. Rev. B* **1988**, *37*, 785–789.

[17] A. D. Becke, *Phys. Rev. A* **1988**, *38*, 3098–3100.

[18] A. D. Becke, *J. Chem. Phys.* **1993**, *98*, 5648–5652.

[19] P. J. Stephens, F. J. Devlin, C. F. Chabalowski, M. J. Frisch, *J. Phys. Chem.* **1994**, *98*, 11623–11627.

[20] D. Peng, N. Middendorf, F. Weigend, M. Reiher, *J. Chem. Phys.* **2013**, *138*, 184105.

[21] Y. J. Franzke, N. Middendorf, F. Weigend, *J. Chem. Phys.* **2018**, *148*, 104110.

[22] Y. J. Franzke, J. M. Yu, *J. Chem. Theory Comput.* **2022**, *18*, 323–343.

[23] Y. J. Franzke, F. Mack, F. Weigend, *J. Chem. Theory Comput.* **2021**, *17*, 3974–3994.

[24] Y. J. Franzke, F. Weigend, *J. Chem. Theory Comput.* **2019**, *15*, 1028–1043.

[25] L. Cheng, J. Gauss, *J. Chem. Phys.* **2011**, *135*, 084114.

[26] L. Cheng, J. Gauss, *J. Chem. Phys.* **2011**, *135*, 244104.

[27] L. Cheng, J. Gauss, J. F. Stanton, *J. Chem. Phys.* **2013**, *139*, 054105.

[28] D. Peng, M. Reiher, *J. Chem. Phys.* **2012**, *136*, 244108.

[29] E. Caldeweyher, S. Ehlert, A. Hansen, H. Neugebauer, S. Spicher, C. Bannwarth, S. Grimme, *J. Chem. Phys.* **2019**, *150*, 154122.

[30] E. Caldeweyher, C. Bannwarth, S. Grimme, *J. Chem. Phys.* **2017**, *147*, 034112.

[31] M. Garcia-Ratés, F. Neese, *J. Comput. Chem.* **2020**, *41*, 922–939.
[32] V. Barone, M. Cossi, *J. Phys. Chem. A* **1998**, *102*, 1995–2001.
[33] P. Pollak, F. Weigend, *J. Chem. Theory Comput.* **2017**, *13*, 3696–3705.
[34] B. Helmich-Paris, B. de Souza, F. Neese, R. Izsák, *J. Chem. Phys.* **2021**, *155*, 104109.
[35] F. Neese, *J. Comput. Chem.* **2003**, *24*, 1740–1747.
[36] F. Neese, F. Wennmohs, A. Hansen, U. Becker, *Chem. Phys.* **2009**, *356*, 98–109.
[37] T. Yanai, D. P. Tew, N. C. Handy, *Chem. Phys. Lett.* **2004**, *393*, 51–57.
[38] M. Mato, A. Stamoulis, P. Cleto Bruzzese, J. Cornella, *Angew. Chem. Int. Ed.* **2025**, *64*, e202418367.
[39] M. Mato, P. C. Bruzzese, F. Takahashi, M. Leutzsch, E. J. Reijerse, A. Schnegg, J. Cornella, *J. Am. Chem. Soc.* **2023**, *145*, 18742–18747.
[40] A. Stamoulis, M. Mato, P. C. Bruzzese, M. Leutzsch, A. Cadranel, M. Gil-Sepulcre, F. Neese, J. Cornella, *J. Am. Chem. Soc.* **2025**, *147*, 6037–6048.
[41] J. Pipek, P. G. Mezey, *J. Chem. Phys.* **1989**, *90*, 4916–4926.
[42] P. v. R. Schleyer, C. Maerker, A. Dransfeld, H. Jiao, N. J. R. van Eikema Hommes, *J. Am. Chem. Soc.* **1996**, *118*, 6317–6318.
[43] Z. Chen, C. S. Wannere, C. Corminboeuf, R. Puchta, P. v. R. Schleyer, *Chem. Rev.* **2005**, *105*, 3842–3888.
[44] K. Kitaura, K. Morokuma, *Int. J. Quantum Chem.* **1976**, *10*, 325–340.
[45] M. Mitoraj, A. Michalak, *Organometallics* **2007**, *26*, 6576–6580.
[46] M. Mitoraj, A. Michalak, *J. Mol. Model.* **2008**, *14*, 681–687.
[47] M. P. Mitoraj, A. Michalak, T. Ziegler, *J. Chem. Theory Comput.* **2009**, *5*, 962–975.
[48] R. C. Sabando, C. Riplinger, F. Wennmohs, F. Neese, G. Bistoni, *J. Chem. Theory Comput.* **2025**, *21*, 7920–7934.
[49] T. Ziegler, A. Rauk, *Inorg. Chem.* **1979**, *18*, 1755–1759.
[50] T. Ziegler, A. Rauk, *Inorg. Chem.* **1979**, *18*, 1558–1565.
